# T cell acute lymphoblastic leukemia exploits a neural proinflammatory pathway to colonize the meninges

**DOI:** 10.1172/JCI188888

**Published:** 2025-10-23

**Authors:** Nitesh D. Sharma, Esra’a Keewan, Wojciech Ornatowski, Silpita Paul, Monique Nysus, Christopher C. Barnett, Julie Wolfson, Quiteria Jacquez, Bianca L. Myers, Huining Kang, Katherine E. Zychowski, Stuart S. Winter, Mignon L. Loh, Stephen P. Hunger, Eliseo F. Castillo, Tom Taghon, Christina Halsey, Tou Yia Vue, Nicholas Jones, Panagiotis Ntziachristos, Ksenia Matlawska-Wasowska

**Affiliations:** 1 Department of Cell Developmental and Integrative Biology, University of Alabama at Birmingham, Birmingham, Alabama, USA.; 2 Department of Pediatrics, University of New Mexico, Albuquerque, New Mexico, USA.; 3 Department of Pediatrics, University of Alabama at Birmingham, Birmingham, Alabama, USA.; 4 College of Nursing,; 5 Department of Neurosciences, and; 6 Department of Internal Medicine, University of the New Mexico, Albuquerque, New Mexico, USA.; 7 Children’s Minnesota, Minneapolis, Minnesota, USA.; 8 Seattle Children’s Research Institute and the Department of Pediatrics, Seattle Children’s Hospital, University of Washington, Seattle, Washington, USA.; 9 Department of Pediatrics and the Center for Childhood Cancer Research, Children’s Hospital of Philadelphia, University of Pennsylvania, Philadelphia, Pennsylvania, USA.; 10 Department of Internal Medicine, Division of Gastroenterology and Hepatology, University of New Mexico, Albuquerque, New Mexico, USA.; 11 Cancer Research Institute Ghent, Ghent, Belgium.; 12 Department of Diagnostic Sciences, Ghent University, Ghent, Belgium.; 13 Wolfson Wohl Cancer Research Centre, College of Medical Veterinary and Life Sciences, University of Glasgow, Glasgow, United Kingdom.; 14 Institute of Life Science, Swansea University Medical School, Swansea, Wales, United Kingdom.; 15 Department of Biomolecular Medicine, Ghent University, Ghent, Belgium.

**Keywords:** Inflammation, Oncology, Cell migration/adhesion, Chemokines, Leukemias

## Abstract

Infiltration of T cell acute lymphoblastic leukemia (T-ALL) into the meninges worsens prognosis, underscoring the need to understand mechanisms driving meningeal involvement. Here, we show that T-ALL cells expressing CXCR3 exploit normal T cell function to infiltrate the inflamed meninges. CXCR3 deletion hampered disease progression and extramedullary dissemination by reducing leukemic cell proliferation and migration. Conversely, forced expression of CXCR3 facilitated T-ALL trafficking to the meninges. We identified the ubiquitin-specific protease 7 as a key regulator of CXCR3 protein stability in T-ALL. Furthermore, we discovered elevated levels of CXCL10, a CXCR3 ligand, in the cerebrospinal fluid from patients with T-ALL and leukemia-bearing mice. Our studies demonstrate that meningeal stromal cells, specifically pericytes and fibroblasts, induce CXCL10 expression in response to leukemia and that loss of CXCL10 attenuated T-ALL influx into the meninges. Moreover, we report that leukemia-derived proinflammatory cytokines, TNF-α, IL-27, and IFN-γ, induced CXCL10 in the meningeal stroma. Pharmacological inhibition or deletion of CXCR3 or CXCL10 reduced T-ALL cell migration and adhesion to meningeal stromal cells. Finally, we reveal that CXCR3 and CXCL10 upregulated VLA-4/VCAM-1 signaling, promoting cell-cell adhesion and thus T-ALL retention in the meninges. Our findings highlight the pivotal role of CXCR3-CXCL10 signaling in T-ALL progression and meningeal colonization.

## Abstract 2

T-cell leukemia exploits an inflammatory pathway to invade the brain’s protective layers, revealing a potential new target for therapies aimed at preventing disease progression

## Graphical abstract

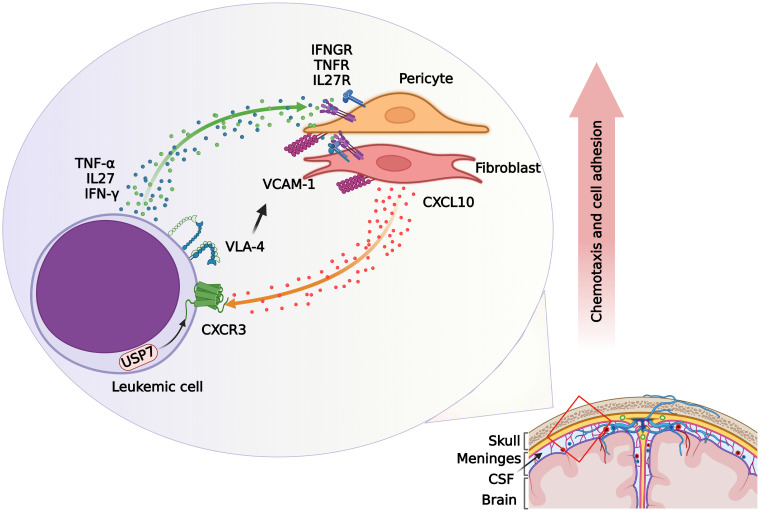

## Introduction

T cell acute lymphoblastic leukemia (T-ALL) is an aggressive hematologic cancer that arises from the malignant transformation of T cell progenitors (1). It comprises 15% of pediatric and 25% of adult cases with ALL. Patients with T-ALL present with hyperleukocytosis, infiltration of the BM and extramedullary sites, including the CNS (2). Intensified chemotherapy has improved cure rates in T-ALL (3–5). However, survival outcomes remain poor among refractory and relapsed patients, including those with CNS involvement (3, 4, 6–8). In CNS-involved ALL, leukemic blasts invade the meninges and circulate in the cerebrospinal fluid (CSF) (9). CNS infiltration is routinely detected using cytomorphology-based analyses of CSF (10–12). However, growing evidence questions the accuracy of cytospin-based cytology to detect individuals with occult CNS leukemia (9, 12–14).

The implementation of modern risk-adapted CNS-directed therapies combining systemic treatment with intrathecal chemotherapy has resulted in lower rates of CNS relapse (3, 5, 6). However, the optimal treatment strategies for treating CNS disease or CNS relapse while minimizing therapy toxicities have not been identified thus far. Therefore, identifying mechanisms underlying leukemic cell colonization and retention in the CNS is necessary for the development of future targeted therapies.

In CNS leukemia, leukemic cells infiltrate the meninges, which are a specialized set of membranes that enclose the brain and spinal cord, providing essential mechanical support and protection. The meningeal microenvironment is heterogeneous and exhibits a diverse repertoire of immune cell populations and stromal cells, including endothelial cells, pericytes, and fibroblasts (15, 16). Furthermore, the meningeal microenvironment tightly regulates immune cell recruitment and retention to maintain a balance between immune defense and prevention of neuroinflammation (17).

Several studies have identified specific receptors involved in the migration and infiltration of leukemic cells into the CNS (18–29). To date, CCR7 and its chemokine CCL21 were identified to be necessary to drive T-ALL migration to the meninges (30). Moreover, the chemokine receptor, CXCR4 has been shown to regulate T-ALL progression and homing to the BM and the meninges (31–36). It is becoming increasingly evident that the meningeal microenvironment impacts leukemic cell ability to invade and reside in the meninges (26, 28, 37–39). In turn, disseminated leukemic cells exploit the meninges to create a supportive niche for leukemic cell survival.

The hallmark of neuroinflammation is the influx of leukocytes across the blood-brain or blood-CSF barrier. The entry of leukocytes into the CNS/meninges is regulated by chemokines (17, 40). Inflammatory chemokine expression is typically low in the resting CNS/meninges but can be upregulated during inflammation (17). CXCL10, known as an inflammatory chemokine, controls the entry of various leukocyte subsets into the meninges and other tissues during inflammation (41–44). CXCL10 is expressed by neurons, glia, and stromal cells in multiple meningeal diseases (41–46). Elevated levels of CXCL10 have been associated with invasiveness and metastatic potential in solid tumors (47–49). Interestingly, increased levels of CXCL10 have been detected in the CSF of patients with T-ALL with CNS involvement (31). CXCL10 binds to CXCR3, which is predominantly expressed on activated T cells and regulates T cell trafficking into extramedullary sites such as the brain and meninges (43, 50–55). Interestingly, several lines of evidence suggest that CXCR3 contributes to the metastasis of various solid tumors (47, 48, 56–58).

Similar to normal T cells, T-ALL cells possess unique migratory and homing abilities (22, 59, 60). Given the implication of CXCR3-CXCL10 signaling in the migration of normal T cells across the blood–CSF barrier during inflammation (43, 50, 54), we hypothesized that T-ALL cells may exploit normal T cell function and adopt proinflammatory pathways to facilitate leukemic cell migration and dissemination into the meninges. In this study, we investigated the role of CXCR3-CXCL10 signaling in T-ALL infiltration and retention in the meninges. Our findings support the mechanism by which T-ALL hijacks the CXCR3-CXCL10 pathway to colonize the meningeal niche, underscoring the potential for targeting this pathway in T-ALL.

## Results

### CXCR3 is expressed in human T-ALL and murine ΔE-Notch1-driven T-ALL.

**Figure 1:**
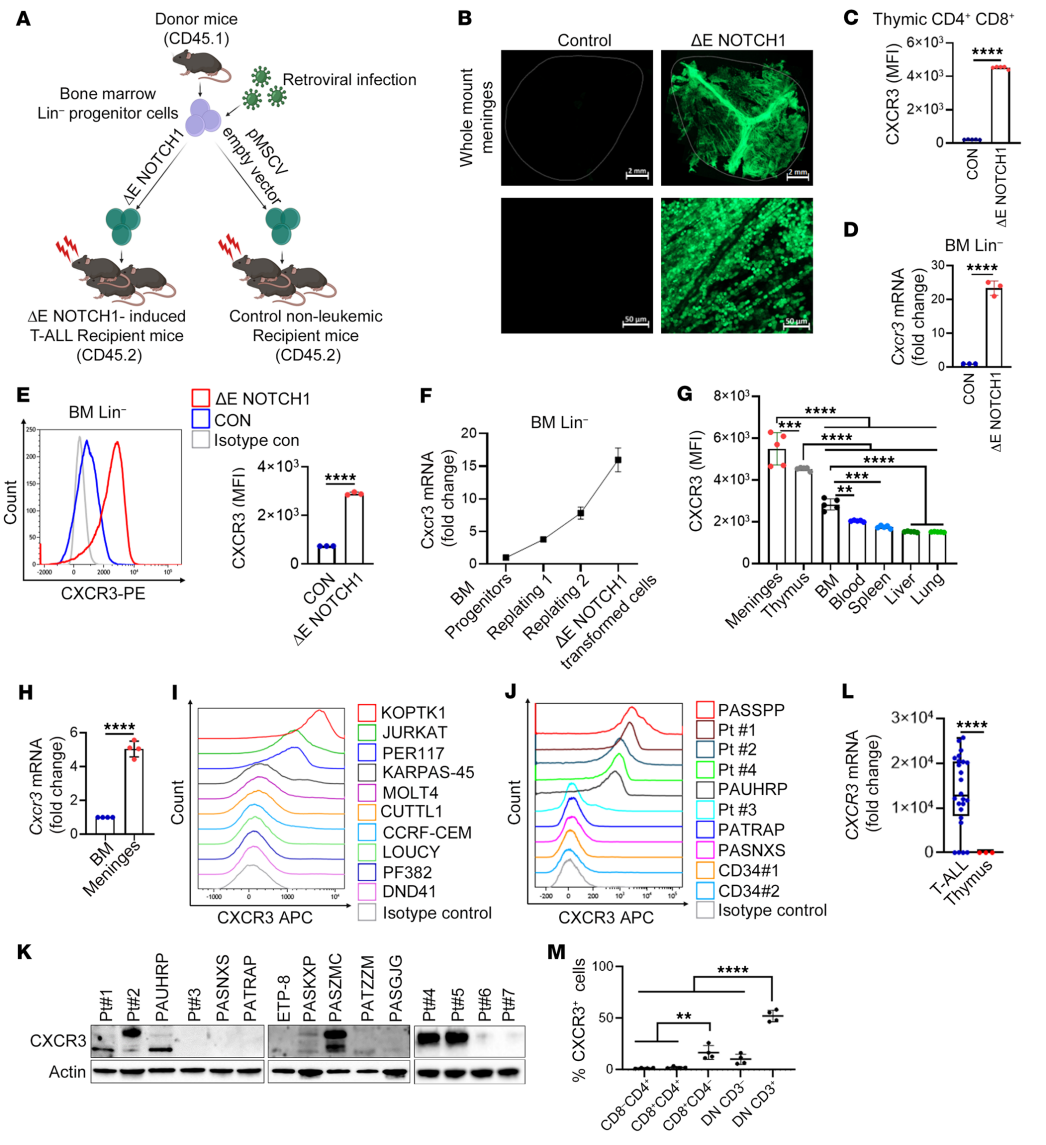
CXCR3 is expressed in T-ALL. (**A**) A schematic diagram for the generation of oncogenic *ΔE-NOTCH1*–driven T-ALL. (**B**) Confocal images of whole-mount meninges from *ΔE-NOTCH1* and control mice showing infiltration of GFP-expressing (green) leukemic cells. Representative images (3 mice/group). Scale bars: 2 mm (top row), 50 μm (bottom row). (**C**) Cell surface CXCR3 on CD4^+^CD8^+^ DP cells isolated from the thymus of leukemic (ΔE-NOTCH1) and control (CON) mice (*n* = 5/group). Data represent median fluorescent intensity (MFI) ± SD. (**D**) *Cxcr3* expression in *ΔE-NOTCH1*-transformed (ΔE-NOTCH1) and control (CON) Lin^–^ hematopoietic progenitors. Mean ± SD, 3 independent experiments. (**E**) CXCR3 levels in *ΔE-NOTCH1-*transformed and control hematopoietic progenitors; representative histograms (left); MFI ± SD, 3 separate experiments (right). (**F**) Expression of *Cxcr3* during *ΔE-NOTCH1*-driven transformation (mean ± SD, 3 independent experiments). (**G**) CXCR3 levels in T-ALL cells (CD4^+^CD8^+^ DP/GFP^+^) isolated from distinct organs of moribund *ΔE-NOTCH1* mice (*n* = 5); MFI ± SD. (**H**) *Cxcr3* expression in T-ALL (CD4^+^CD8^+^ DP/GFP^+^) cells isolated from the BM and meninges of moribund *ΔE-NOTCH1* mice (*n* = 4). Mean ± SD. Representative histograms for CXCR3 levels in (**I**) T-ALL cell lines (*n* = 10), (**J**) primary T-ALL cells (*n* = 8), and normal CD34^+^ cells (*n* = 2). (**K**) Immunoblotting for CXCR3 in primary T-ALL cells (*n* = 15). Representative blots from at least 2 separate experiments. (**L**), *CXCR3* expression in primary T-ALL samples (*n* = 24) and normal thymocytes (Thymus, *n* = 3). (**M**), CXCR3 expression in normal human thymic T cell subsets (*n* = 4 donors). The data show the percentage of receptor-positive cells in each subset. Mean ± SD. (**A**) Illustrations were created with BioRender.com. (**C**–**E**, **H**, and **L**) unpaired 2-tailed *t* test. (**G** and **M**) 1-way ANOVA with Tukey’s multiple comparison test; *P < 0.05; **P < 0.005; ***P < 0.0005; ****P < 0.0001.

We first studied *CXCR3* expression in T-ALL using a murine model of *ΔE-NOTCH1*-induced T-ALL (Figure 1A and Supplemental Figure 1, A–D; supplemental material available online with this article; https://doi.org/10.1172/JCI188888DS1) (61). This model leads to the development of T-ALL with meningeal infiltration (Figure 1B) (19). We evaluated CXCR3 expression in thymic CD4^+^CD8^+^ double positive (DP) cells of *ΔE-NOTCH1* T-ALL and control nonleukemic mice. The levels of CXCR3 were higher in CD4^+^CD8^+^ DP cells of leukemia-bearing mice compared with control animals (Figure 1C). We also observed an upregulation of *Cxcr3* mRNA and cell surface protein levels in murine BM lineage negative (Lin^–^) progenitors transduced with ΔE NOTCH1 (Figure 1, D and E). *Cxcr3* mRNA levels gradually increased at each time point, suggesting that NOTCH1 regulates *Cxcr3* (Figure 1F). Accordingly, targeting NOTCH1 signaling with DBZ, a γ-secretase inhibitor, reduced CXCR3 mRNA and protein levels in both human and murine T-ALL cells (Supplemental Figure 1, E–H). We next examined the expression of CXCR3 in T-ALL cells isolated from distinct sites of leukemic cell infiltration. The highest levels of cell surface CXCR3 were found in leukemic cells (GFP^+^/CD4^+^CD8^+^DP) colonizing the meninges, thymus, and BM, as opposed to the liver, spleen, lungs, and blood in *ΔE NOTCH1* T-ALL mice (Figure 1G and Supplemental Figure 1I). We confirmed that T-ALL cells colonizing the meninges had higher *Cxcr3* mRNA levels compared with leukemic cells in the BM (Figure 1H). We next investigated CXCR3 expression in human T-ALL cell lines (*n* = 10). TAL1-expressing KOPTK1 and Jurkat cells, and early T cell precursor (ETP) phenotype PER117 cell line had higher levels of CXCR3 compared with other tested cell lines (Figure 1I). Flow cytometry analyses and immunoblotting of primary T-ALL samples (*n* ≤ 15), further confirmed differential CXCR3 levels in T-ALL cells compared with human CD34^+^ progenitors (*n* = 2) and mature CD4^+^ (*n* = 5) and CD8^+^ cells (*n* = 5), which had lower CXCR3 expression (Figure 1, J and K, Supplemental Figure 1J, and Supplemental Table 1). Furthermore, the levels of *CXCR3* mRNA were higher in primary T-ALL samples (*n* = 24) compared with normal thymic cells (*n* = 3) (Figure 1L). Molecular interrogation of normal human thymic T cell subsets confirmed lower expression of CXCR3 on CD4^+^/CD^+^ DP cells, CD4^+^ cells, and CD4^–^/CD8^–^ double negative (DN) DN/CD3^–^ cells compared with DN/CD3^+^ (γδ) and CD8^+^ cells (Figure 1M and Supplemental Figure 1K). Of note, DN/CD3^+^ and CD8^+^ cells represent a small fraction (approximately 1% and 5%, respectively) of the total thymocytes in thymus. Analyses of published datasets for pediatric, adolescent, and young adult patients with T-ALL (62, 63) showed that increased expression of *CXCR3* was not associated with any T-ALL molecular subtype or genetic lesion (not shown). Collectively, these results demonstrate that CXCR3 is differentially expressed in T-ALL in a tissue-specific manner.

### CXCR3 regulates T-ALL cell proliferation and disease progression.

**Figure 2:**
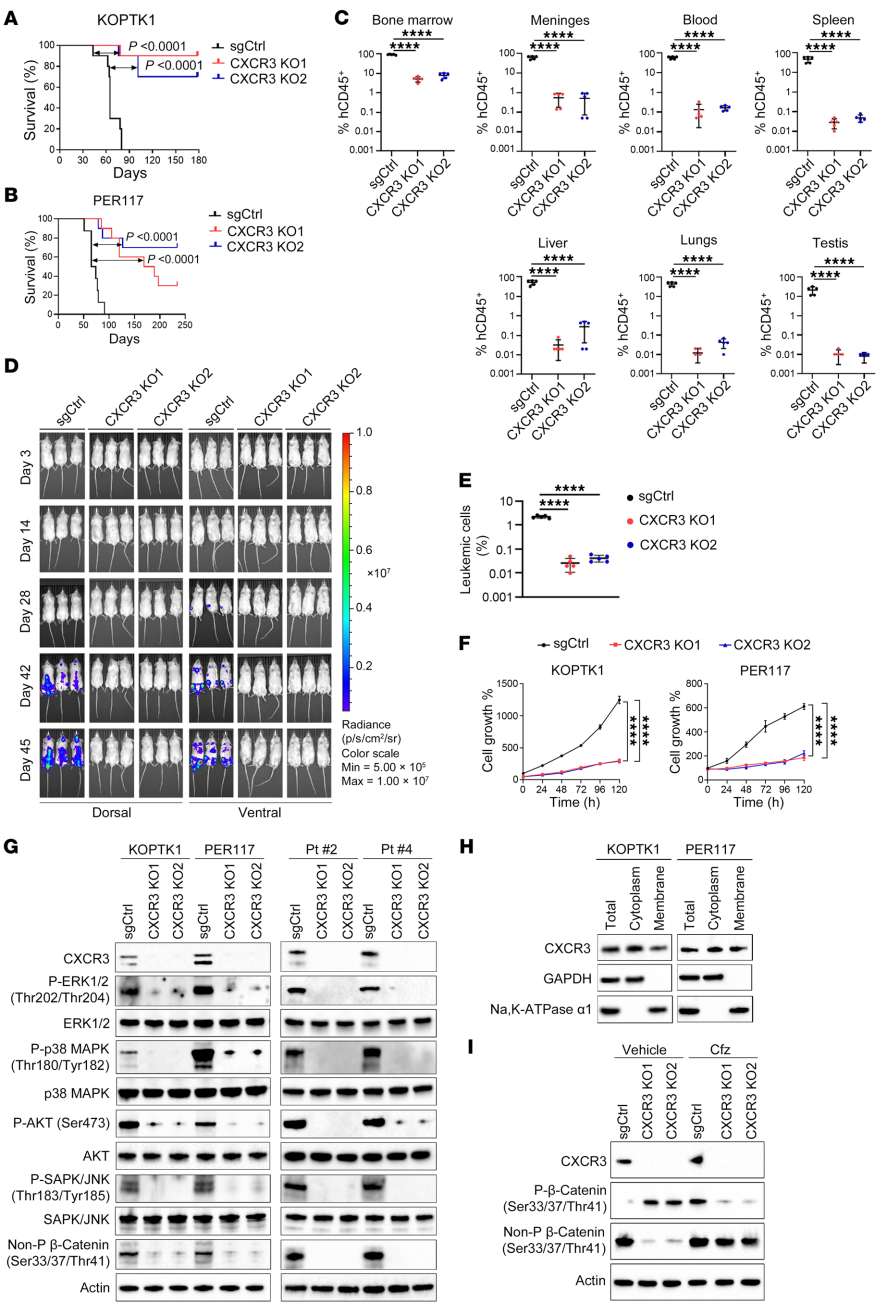
CXCR3 promotes T-ALL cell proliferation and disease progression. KOPTK1 and PER117 T-ALL cell lines were transduced with lentivirus expressing sgRNAs targeting *CXCR3* (CXCR3 KO1 and CXCR3 KO2) and a negative control sgRNA (sgCtrl). Kaplan-Meier survival curve analyses of NSG mice (*n* = 8/group) transplanted with 1 × 10^6^ transduced (**A**) KOPTK1 and (**B**) PER117 cells (log-rank test). (**C**) Quantification of human CD45^+^ cells in distinct organs of NSG mice (*n* = 5/group) euthanized 45 days after receiving intrafemoral implantation with 3 × 10^5^ transduced KOPTK1 cells. (**D**) Bioluminescence imaging of NSG mice (*n* = 3/group) inoculated intrafemorally with transduced KOPTK1 cells (3 × 10^5^) coexpressing firefly luciferase. (**E**) Homing of T-ALL cells in the BM at 24 hours. NSG mice (*n* = 5/group) received intravenously 1 × 10^7^ fluorescently labeled (DsRed) transduced KOPTK1 cells. (**F**) Cell growth of KOPTK1 and PER117 transduced with sgRNAs targeting *CXCR3* (CXCR3 KO1, CXCR3 KO2) and a negative control sgRNA (sgCtrl). Mean ± SD for 1 of 3 independent experiments performed in triplicate; repeated measure ANOVA with Tukey’s multiple comparisons test. (**G**) Immunoblotting of KOPTK1, PER117, and primary T-ALL cells (Pt #2, Pt #4) with the indicated antibodies. (**H**) Cytoplasmic and membrane-associated CXCR3 fractions in T-ALL cells. Cytoplasmic GAPDH and membrane Na, K-ATPase α1 served as controls. (**I**) KOPTK1 cells were treated with an irreversible proteasome inhibitor, carfilzomib (Cfz; 0.5 nM, 6 h) or vehicle control (Vehicle), followed by immunoblotting with the indicated antibodies. (**G**–**I**) Representative blots from at least 3 separate experiments. (**C** and **E**) Data are shown as mean ± SD. One-way ANOVA with Tukey’s multiple comparison test; ****P < 0.0001.

To examine the effect of CXCR3 on T-ALL progression, we performed CRISPR/Cas9–mediated knockout (KO) of *CXCR3* in KOPTK1 and PER117 cell lines, which exhibited higher levels of CXCR3 expression (Supplemental Figure 2, A–C). NOD.Cg-*Prkdc^scid^Il2rg^tm1Wjl^*/SzJ (NSG) mice were transplanted with KOPTK1 and PER117 cells transduced with control (sgCtrl) and sgRNAs targeting *CXCR3* (*CXCR3* KO1 and *CXCR3* KO2). We observed prolonged survival of mice injected intravenously with *CXCR3* knockout cells compared with animals inoculated with control cells (Figure 2, A and B). Tissue examination of animals injected intrafemorally, which were all euthanized 45 days after inoculation, revealed lower levels of KOPTK1 cells in the BM and limited infiltration of T-ALL cells into extramedullary sites such as the meninges and other organs, compared with the control group mice (Figure 2, C and D, and Supplemental Figure 2D). We further investigated whether CXCR3 regulates T-ALL cell homing into the BM. We recovered a lower number of T-ALL cells from the femurs of mice inoculated with *CXCR3* knockout cells compared with animals injected with control cells (Figure 2E). Functionally, *CXCR3* knockout in KOPTK1 and PER117 cells resulted in decreased cell proliferation and a delay in cell cycle progression in S and G2/M phases, accompanied by an increase in G0/G1 phases (Figure 2F and Supplemental Figure 2E). There was no effect on apoptotic cell death in the tested cells upon *CXCR3* deletion (Supplemental Figure 2F). Using T-ALL cell lines (KOPTK1, PER117) and primary samples (Pt #2, Pt #4), we demonstrated that *CXCR3* knockout reduced the activation of ERK1/2, p38, AKT, and SAPK/JNK, along with β-catenin pathways, which are known to regulate T-ALL cell proliferation and signal transduction (Figure 2G) (29). With evidence that CXCR3 regulates cell signaling pathways in the absence of chemokine stimulation (Figure 2G), we next examined its cellular localization. In line with prior observations (64, 65), we detected both membrane-bound and cytoplasmic fractions of CXCR3 in T-ALL cells under steady-state and ligand-stimulated conditions (Figure 2H and Supplemental Figure 2G). Interestingly, forced CXCR3 expression restored nonphosphorylated, active β-catenin, and, to a lesser degree, ERK1/2, p38, AKT, and SAPK/JNK activation, rescuing T-ALL cell proliferation (Supplemental Figure 2, H and I). To further determine whether CXCR3 stabilizes β-catenin, we treated *CXCR3* knockout cells with the proteasome inhibitor carfilzomib, which restored active β-catenin (Figure 2I), suggesting that CXCR3 prevents β-catenin proteasomal degradation under steady-state conditions. Consistently, expression of constitutively active β-catenin in CXCR3-knockdown cells partially rescued CXCR3 levels and T-ALL cell proliferation (Supplemental Figure 2, J and K). Finally, cell fractionation revealed restored cytoplasmic and nuclear β-catenin in CXCR3-rescued cells (Supplemental Figure 2L), further supporting a role of CXCR3 in stabilizing β-catenin and potentially regulating its transcriptional activity. Together, these findings suggest that CXCR3 promotes T-ALL cell proliferation and disease progression.

### CXCR3 mediates T-ALL cell migration and infiltration into the meninges.

**Figure 3:**
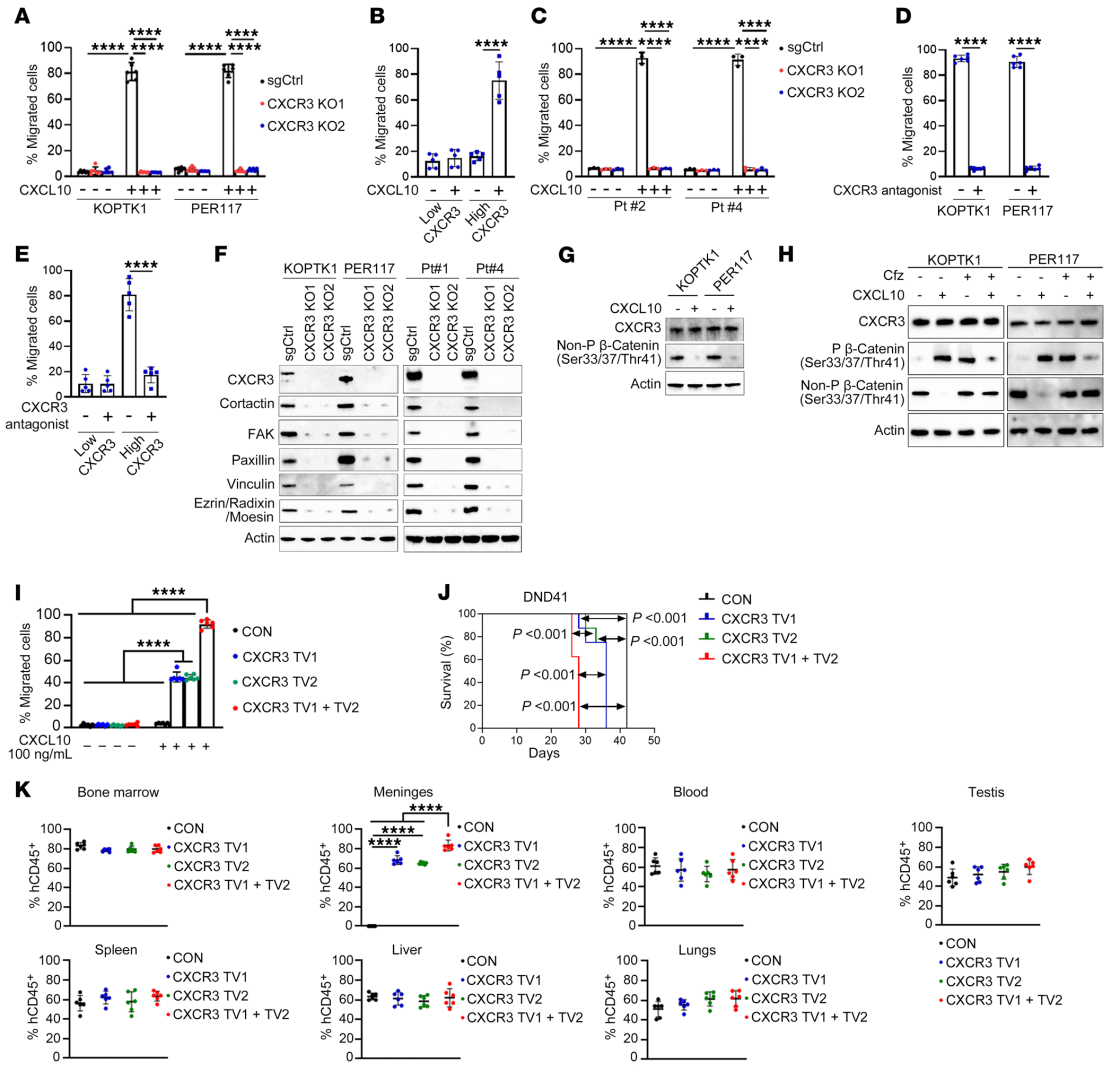
CXCR3 regulates T-ALL cell migration and infiltration into the meninges. (**A**) Migration of T-ALL cells upon CXCR3 knockout ± CXCL10 (100 ng/mL, 6 h, 3 μm transwell membrane) (**B**) Primary T-ALL cells were stratified based on CXCR3 cell surface expression as high (*n* = 5) and low (*n* = 5), followed by cell migration assay ± CXCL10 (100 ng/mL, 6 h). (**C**) Migration of primary T-ALL cells (Pt #2, Pt #4) upon CXCR3 deletion ± CXCL10 (100 ng/mL, 6 h). (**D**) T-ALL cell lines and (**E**) primary cells grouped as CXCR3 high (*n* = 5) and low (*n* = 5) were pretreated with a CXCR3 antagonist, AMG487 (1.5 μg, 30 min.). Cell migration ± CXCL10 (100 ng/mL; 6 h). (**F**–**H**) Immunoblotting for specified proteins (Cfz, carfilzomib, 0.5 nM, 6 h; CXCL10, 100 ng/mL, 1 h). Representative blots from at least 3 separate experiments. (**I**) DND41 cells were transduced to express either CXCR3 variants (CXCR3 TV1, CXCR3 TV2, CXCR3 TV1 + TV2) or a negative control plasmid (CON). Cell migration in the presence or absence of CXCL10 (100 ng/ml, 6 h). Mean ± SD, 3 separate experiments performed in duplicate. (**J**) Kaplan-Meier survival curve analysis of NSG mice (*n* = 8/group) intravenously inoculated with 1 × 10^6^ transduced DND41 cells (log-rank test). (**K**) Human CD45^+^ cells isolated from various organs of moribund NSG mice (*n* = 6/group). The percentage of T-ALL cells (% hCD45^+^) was calculated as [hCD45^+^/(hCD45^+^ + mCD45^+^)] × 100. (**A**, **C**, and **I**) Mean ± SD, 3 separate experiments performed in duplicate (**B**, **D**, and **E**) Mean ± SD, each sample was tested in duplicate. Unpaired 2-tailed *t* test with Holm-Šidák correction for multiple comparisons. (**A**, **C**, and **I**) Two-way ANOVA and (**K**) 1-way ANOVA with Tukey’s multiple comparison test; ****P < 0.0001.

Given the role of CXCR3 and its chemokines CXCL9, CXCL10 and CXCL11 in immune cell trafficking (66), we next studied how CXCR3 regulates leukemic cell migration. We observed enhanced migration of KOPTK1 and PER117 cells to CXCL10 compared with CXCL9 and CXCL11, and subsequently reduced T-ALL cell migration upon *CXCR3* silencing (Figure 3A and Supplemental Figure 3, A–C). As CXCL10 induced higher levels of T-ALL cell migration than CXCL9 and CXCL11, CXCL10 was selected for further validation studies. We tested a small set of primary T-ALL samples, stratified based on cell surface CXCR3 expression levels (CXCR3^high^, *n* = 5 or CXCR3^low^, *n* = 5). Samples with higher CXCR3 levels showed increased migration to CXCL10 compared with lower CXCR3 expressors (Figure 3B). *CXCR3* deletion in 2 primary T-ALL samples (Pt #2, Pt #4) resulted in reduced leukemic cell migration to CXCL10 (Figure 3C). Furthermore, pharmacological inhibition of CXCR3 with AMG487, a CXCR3 antagonist, reduced migratory capacity of both T-ALL cell lines and primary cells to CXCL10 (Figure 3, D and E) but had no effect on T-ALL cell proliferation and viability (Supplemental Figure 3, D–F). Consistently, murine *ΔE-NOTCH1*–transformed T-ALL cells exhibited migration toward a CXCL10 gradient (Supplemental Figure 3G). Although CXCL10 induced CXCR3 internalization, it was not expressed in T-ALL, and did not affect T-ALL cell proliferation (Supplemental Figure 3, H–K). As transendothelial migration is critical for leukemic cell dissemination, we further investigated the role of CXCR3 in T-ALL cell migration through a monolayer of human umbilical vein endothelial cells (HUVEC) in the presence or absence of CXCL10. Knockout of *CXCR3* in KOPTK1 and PER117, and in primary T-ALL cells (Pt #2, Pt #4), reduced leukemic cell migration through HUVEC (Supplemental Figure 3, L and M). Functionally, loss of *CXCR3* expression in T-ALL cells resulted in reduced expression of critical regulators of cell motility, including cortactin, vinculin, paxillin, focal adhesion kinase (FAK), and ezrin-radixin-moesin (ERM) (Figure 3F). In contrast, stimulation of T-ALL cells with CXCL10 led to increased levels of these migration-associated proteins (Supplemental Figure 3N). Notably, CXCL10 treatment selectively decreased the levels of active, nonphosphorylated (Ser33/Ser37/Thr41) β-catenin (Figure 3G) without affecting the activation of ERK1/2, p38, AKT, and SAPK/JNK signaling pathways (Supplemental Figure 3O), suggesting a specific role for β-catenin in mediating the cellular response to CXCL10. Treatment with the proteasomal inhibitor carfilzomib restored active β-catenin levels in CXCL10-treated cells, suggesting that CXCL10 engagement promotes β-catenin phosphorylation (Ser33/Ser37/Thr41) and its subsequent degradation in the proteasome (Figure 3H). These findings reveal the mechanistic link between CXCL10-mediated signaling and β-catenin turnover in T-ALL. We next induced ectopic expression of CXCR3 in the DND41 cell line, which has previously been reported to lack meningeal infiltration (30) and does not express CXCR3, CCR7, or CXCR4 (Supplemental Figure 3, P-T). Forced expression of CXCR3 in DND41 cells induced migration to CXCL10 compared with CXCL9 and CXCL11, without affecting cell proliferation, cell cycle, or apoptosis (Figure 3I and Supplemental Figure 3, U–Y). Upregulation of CXCR3 led to decreased survival in mice compared with the control group (Figure 3J). Strikingly, while control animals developed T-ALL with infiltration of multiple organs but no meningeal involvement, forced CXCR3 expression drove robust meningeal infiltration, underscoring a potential role for CXCR3 in directing T-ALL cell trafficking to the meninges (Figure 3K and Supplemental Figure 3Z). Collectively, these results suggest that CXCR3 regulates T-ALL cell migration and promotes meningeal infiltration.

### USP7 regulates CXCR3 stability by deubiquitylation.

**Figure 4:**
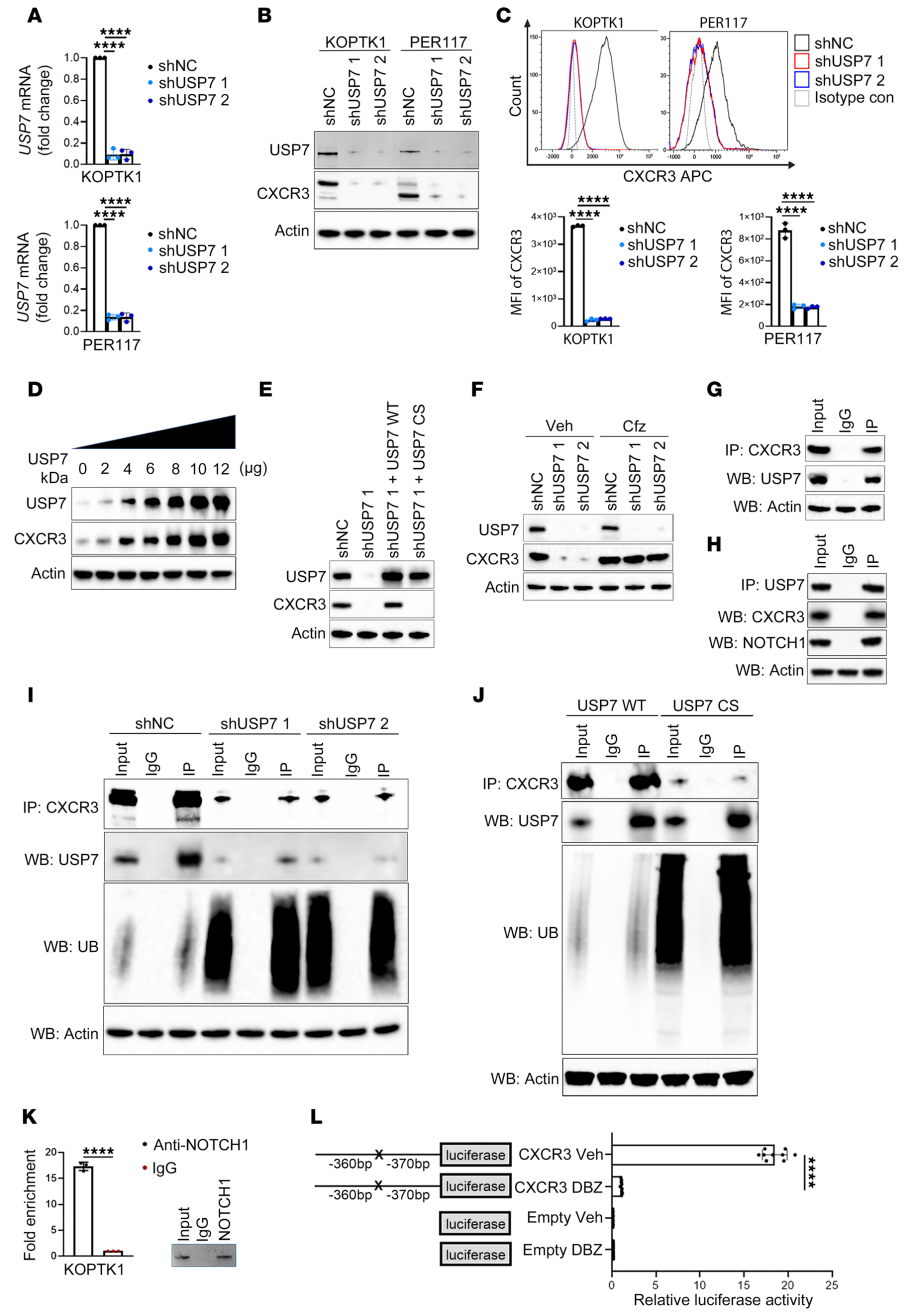
USP7 stabilizes CXCR3 in T-ALL. T-ALL cells were transduced with shRNA targeting *USP7* (shUSP7 1 and shUSP7 2) and scrambled control (shNC), and USP7 (**A**) transcript and (**B**) protein were confirmed. Means ± SD for 3 independent experiments. (**C**) CXCR3 cell surface levels; representative histograms (top); MFI ± SD, 3 separate experiments (bottom). (**D**) CUTTL1 T-ALL cells were transduced with increasing concentrations of *USP7*-expressing plasmid, followed by immunoblotting for CXCR3. (**E**) KOPTK1 cells expressing shRNA *USP7* (shUSP7 1) and scrambled control (shNC) were transduced with plasmids expressing USP7 WT and catalytically inactive *USP7*^C233S^ mutant (USP7 CS). Immunoblotting of the indicated proteins. (**F**) KOPTK1 cells with *USP7* knockdown (shUSP7 1, shUSP7 2) or control cells (shNC) were treated with an irreversible proteasome inhibitor, carfilzomib (Cfz; 0.5 nM, 6 h) or vehicle control (Veh), followed by immunoblotting with the indicated antibodies. Immunoprecipitation of endogenous (**G**) CXCR3 and (**H**) USP7 in KOPTK1 cells under denaturing conditions, followed by immunoblotting for the specified proteins. (**I**) Immunoprecipitation of CXCR3 in KOPTK1 carrying USP7 knockdown (shUSP7 1 or shUP7 2) or scrambled control (shNC), followed by Western blot for USP7 and ubiquitin (UB). (**J**) Immunoprecipitation of CXCR3 in KOPTK1 cells expressing USP7 WT and catalytically inactive *USP7*^C233S^ mutant USP7 CS. Immunoblotting analysis of the specified proteins. (**K**) Enrichment of NOTCH1 on the *CXCR3* promoter by ChIP-qPCR in KOPTK1 cells. (**L**) Luciferase reporter assay for *CXCR3* on KOPTK1 following treatment with a γ-secretase inhibitor (DBZ, 0.1 μM, 24 h). The NOTCH1 binding site is indicated as X (–360bp to –370bp upstream of the *CXCR3* coding start site). (**A**, **C**, and **L**) One-way ANOVA with Tukey’s multiple comparisons test. (**K**) Unpaired 2-tailed *t* test; ****P < 0.0001. (**B** and **D**–**J**) Representative blots of 1 of 3 independent experiments.

The ubiquitin-specific protease 7 (USP7) is expressed in T-ALL and transcriptionally regulated by NOTCH1 (67, 68). USP7 binding studies in NOTCH1-driven T-ALL revealed enrichment of CXCR3 signaling components, highlighting a potential regulatory link between USP7 and CXCR3 signaling (68). Based on these findings, we hypothesized that USP7 regulates CXCR3 expression in T-ALL cells. We knocked down *USP7* in KOPTK1 and PER117 cell lines and investigated its effect on CXCR3 expression. We found that USP7 silencing reduced the expression of CXCR3 at both protein and mRNA levels (Figure 4, A–C, and Supplemental Figure 4A). USP7 is known to stabilize its substrate proteins by removing their ubiquitin tags, thus preventing proteasomal degradation. To determine whether USP7 stabilizes CXCR3, we induced increasing concentrations of a USP7-expressing plasmid into the CUTTL1 T-ALL cell line, which expresses low/undetectable levels of endogenous CXCR3 protein. We observed a gradual increase in CXCR3 levels corresponding with increasing expression of USP7, suggesting that USP7 stabilizes CXCR3 (Figure 4D). To further delineate whether the catalytic function of USP7 plays a role in stabilizing CXCR3 protein, we silenced endogenous *USP7* followed by ectopic expression of USP7 WT and USP7 catalytic domain mutant (USP7 CS, C233S) in KOPTK1 cells. The expression of CXCR3 was absent in the cells expressing catalytically inactive USP7 (USP7 CS) as opposed to the cells expressing WT USP7, in which the expression of CXCR3 was present (Figure 4E). These results indicate that the catalytic activity of USP7 is required to regulate CXCR3. Importantly, treatment with the proteasomal inhibitor carfilzomib restored CXCR3 expression in USP7-deficient cells compared with vehicle-treated control cells, suggesting that USP7 maintains CXCR3 levels by preventing its proteasomal degradation (Figure 4F). We next investigated the interaction between USP7 and CXCR3. Endogenous USP7 was detected in immunoprecipitants with anti-CXCR3 but not IgG antibodies (Figure 4G). Conversely, CXCR3 was present in USP7 but not IgG immunoprecipitants (Figure 4H), indicating the presence of a specific interaction between USP7 and CXCR3. Finally, we sought to understand whether USP7 regulates CXCR3 stability by deubiquitylation. We observed that *USP7* silencing led to increased polyubiquitination of CXCR3 in coimmunoprecipitation experiments (Figure 4I). Furthermore, CXCR3 polyubiquitination was increased in T-ALL and HEK293 cells expressing USP7 CS compared to USP7 WT, underscoring the importance of the catalytic function of USP7 in maintaining CXCR3 protein levels (Figure 4J and Supplemental Figure 4B). Prior studies showed that USP7 interacts with NOTCH1 (Figure 4H) in T-ALL to regulate leukemic cell growth (67, 68). We found that targeting NOTCH1 reduced CXCR3 expression (Supplemental Figure 1, A–D), leading us to investigate its direct role. We observed enrichment for the *CXCR3* promoter region in NOTCH1 immunoprecipitants in KOPTK1 cells (Figure 4K). A luciferase reporter assay showed that NOTCH1 inhibition with DBZ decreased *CXCR3* promoter activity, which was abrogated when the NOTCH1-binding site was lost (Figure 4L). Together, these results suggest that USP7/NOTCH1 regulates and stabilizes CXCR3.

### T-ALL induces CXCL10 in the meninges.

**Figure 5:**
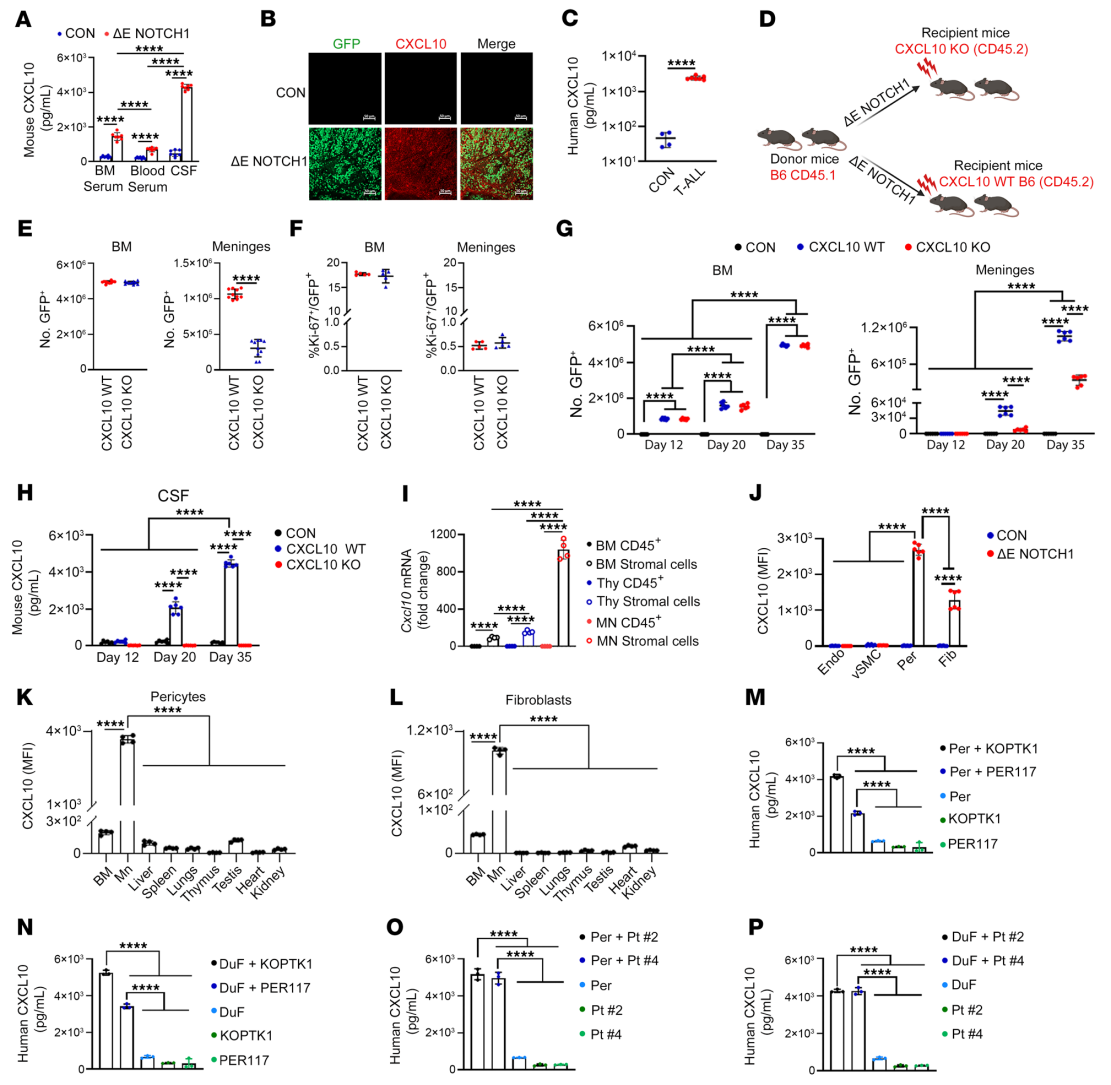
CXCL10 is upregulated in the meningeal microenvironment in T-ALL. (**A**) CXCL10 in body fluids of T-ALL (ΔE-NOTCH1) and control (CON) mice (*n* = 7/group). Scale bars: 50 μm. (**B**) CXCL10 immunolabeling (red) in the meninges of *ΔE-NOTCH1* and control mice. Leukemic cells express GFP (green) (*n* = 3/group). (**C**) CXCL10 in CSF from patients with T-ALL (*n* = 7) and normal CSF samples (*n* = 4). (**D**) Implantation of *ΔE-NOTCH1–*transformed cells into CXCL10 KO and CXCL10 WT. (**E**) CD45^+^/GFP^+^ and (**F**) Ki67^+^/GFP^+^ cell quantification in organs of moribund *ΔE-NOTCH1* T-ALL mice (*n* = 10/group and 5/group, respectively). (**G**) GFP^+^/CD45^+^ cells in the BM and meninges, and (**H**) CXCL10 in CSF of leukemic (CXCL10 WT and CXCL10 KO), and nonleukemic control mice (CON) (*n* = 6/group) (days 12, 20, and 35) (**I**) *Cxcl10* expression in stromal (CD45^–^) and hematopoietic (CD45^+^) cells from BM, meninges (Mn) and thymus (Thy) of *ΔE-NOTCH1* mice (*n* = 4/group). (**J**) CXCL10 in meningeal cell subsets of T-ALL (ΔE-NOTCH1) and control (CON) mice, including fibroblasts (PDGFRα^+^, NG2^–^, CD13^+^, CD31^–^, CD45^–^), pericytes (PDGFRα^+^, NG2^+^, CD13^+^, CD31^–^, CD45^–^), endothelial cells (CD31^+^, CD45^–^), vSMC (Desmin^+^, CD13^+^, CD31^+^, CD45^–^), and hematopoietic cells (CD45^+^) (MFI ± SD, *n* = 6/group). (**K** and **L**) CXCL10 in pericytes and fibroblasts from various organs of *ΔE-NOTCH1* T-ALL mice (MFI ± SD, *n* = 4/group). CXCL10 in the medium of T-ALL cell lines (**M** and **N**) and primary T-ALL (Pt #2, Pt #4) (**O** and **P**) cocultured with/without human primary meningeal stromal cells for 6 h (Per, pericytes; DuF, dural fibroblasts). Mean ± SD, 3 independent experiments. (**A** and **G**–**J**) Two-way ANOVA with Tukey’s multiple comparison test. (**C**) Unpaired 2-tailed *t* test. (**E** and **F**) Unpaired *t* test with Holm-Šidák correction for multiple testing. (**K**–**P**) One-way ANOVA with Tukey’s multiple comparison test; ****P < 0.0001.

Given the role of CXCR3 in regulating T-ALL chemotaxis, we investigated the levels of CXCL9, CXCL10, and CXCL11 in the blood, bone marrow serum, and CSF of *ΔE-NOTCH1* T-ALL and control mice. Leukemic mice had elevated levels of CXCL10 in the blood serum, bone marrow serum, and CSF, compared with control animals (Figure 5A). Interestingly, the levels of CXCL10 were higher in the CSF than in the blood and bone marrow serum of T-ALL–bearing mice, suggesting an enhanced immune response in the CSF microenvironment (Figure 5A). Immunostaining of a whole-mount meninges revealed increased expression of CXCL10 in *ΔE-NOTCH1* mice compared with control animals, where CXCL10 expression was not evident (Figure 5B). In contrast, the levels of CXCL9 in the CSF and meninges of control and ΔE-NOTCH1 mice were low or undetectable (Supplemental Figure 5, A and B). CXCL11 was not detected in murine tissue (not shown), consistent with previous reports indicating that C57BL/6J background mice do not express *Cxcl11* (69). Consistently, we observed increased levels of CXCL10, but not CXCL9, in CSF and meningeal lysates compared with blood and BM samples from moribund NSG mice inoculated with human T-ALL cell lines (Supplemental Figure 5, C and D). Additionally, we observed an increase in CXCL10, but not CXCL9, in the CSF of mice inoculated with DND41 cells overexpressing CXCR3 (Supplemental Figure 5, E and F). Importantly, our findings were further validated by detecting elevated CXCL10 levels in CSF samples from patients with T-ALL (*n* = 7) compared with normal human CSF (*n* = 4) (Figure 5C and Supplemental Table 2). Next, we sought to understand whether CXCL10 mediates leukemic cell infiltration into the meninges. CD45.1 hematopoietic progenitors were transduced with *ΔE-NOTCH1*-GFP followed by transplantation into recipient *CXCL10* knockout (*CXCL10* KO; B6.129S4-*Cxcl10^tm1Adl^*/J) and relevant control mice (CD45.2) (Figure 5D). Postnecropsy tissue analyses performed on terminal mice revealed a decrease in the number of T-ALL cells infiltrating the meninges but not the BM or other organs in *CXCL10*-KO mice compared with the control animals, which presented with high levels of T-ALL cells in the BM and extramedullary tissues (Figure 5E and Supplemental Figure 5G). Loss of CXCL10 did not affect T-ALL cells homing to the BM, suggesting that other factors drive T-ALL to this niche (Supplemental Figure 5H). Notably, we did not observe leukemic cell homing into the meninges of the tested mice (Supplemental Figure 5H), consistent with CXCL10’s role as an inducible inflammatory chemokine, which is absent in noninflamed tissues. Additionally, we did not observe an increase in apoptotic cell levels in T-ALL cells recovered from meninges and BM of *CXCL10*-KO mice compared with control group (Supplemental FIgure 5I). While T-ALL cells exhibited reduced proliferative activity in the meninges compared with BM, the loss of CXCL10 had no effect (Figure 5F). To further investigate how CXCL10 regulates T-ALL infiltration into the meninges, we analyzed leukemia burden and CXCL10 levels in the BM, meninges, and other organs at various time points during *ΔE-NOTCH1-*driven T-ALL development. T-ALL cells gradually increased in the BM of leukemia-bearing mice, independent of CXCL10 knockout (Figure 5G). In contrast, leukemic cells were detected in the meninges at Day 20, with an increase by Day 35 (Figure 5G). T-ALL infiltration into the meninges was delayed compared with the BM. Although CXCL10 knockout did not affect T-ALL infiltration into other organs or the BM, its loss reduced T-ALL infiltration into the meninges (Figure 5G and Supplemental Figure 5J). In line with this, an increase in CXCL10 was observed in the CSF of *ΔE-NOTCH1* mice at Days 20 and 35, while its levels in blood serum samples remained low (Figure 5H and Supplemental Figure 5K). To determine whether the meningeal microenvironment induces CXCL10 in response to T-ALL, we evaluated CXCL10 expression in stromal (CD45^–^) and hematopoietic (CD45^+^) cells isolated from the meninges of control and T-ALL–bearing mice. We observed an increased expression of *Cxcl10* in the meningeal stromal cells (CD45^–^) as opposed to CD45+ cells (Figure 5I). As expected, *Cxcl9* was expressed at low/undetectable levels in the tested cells (Supplemental Figure 5L). Next, we sought to identify specific meningeal stromal cells that induce CXCL10 in response to T-ALL. We found that fibroblasts and pericytes, but not endothelial cells or vSMC, expressed intracellular CXCL10 in *ΔE-NOTCH1* T-ALL mice but not control animals (Figure 5J and Supplemental Figure 5M). Furthermore, CXCL10 levels were elevated in meningeal pericytes and fibroblasts in T-ALL mice relative to their counterparts in other tissues and organs, further supporting a unique role for the meningeal microenvironment in driving CXCL10 induction in response to T-ALL (Figure 5, K and L, and Supplemental Figure 5, N and O). In line, coculturing human T-ALL cell lines or primary cells with primary human leptomeningeal cells (LeC), leptomeningeal pericytes (Per), and dural fibroblasts (DuF), but not dural meningeal endothelial (DuEC), and HUVEC cells, induced secretion of CXCL10 via ELISA (Figure 5, M–P, and Supplemental Figure 5, P–U). Our observations were further supported by the evidence that *CXCL10* was upregulated in Per, DuF, and LeC cocultured with T-ALL cell lines and primary cells, as opposed to stromal cells or leukemic cells cocultured alone (Supplemental Figure 5, V–AA). Collectively, these results demonstrate that T-ALL induces CXCL10 expression by meningeal stromal cells.

### Fibroblast- and pericyte-derived CXCL10 regulate CXCR3-mediated T-ALL cell migration.

**Figure 6:**
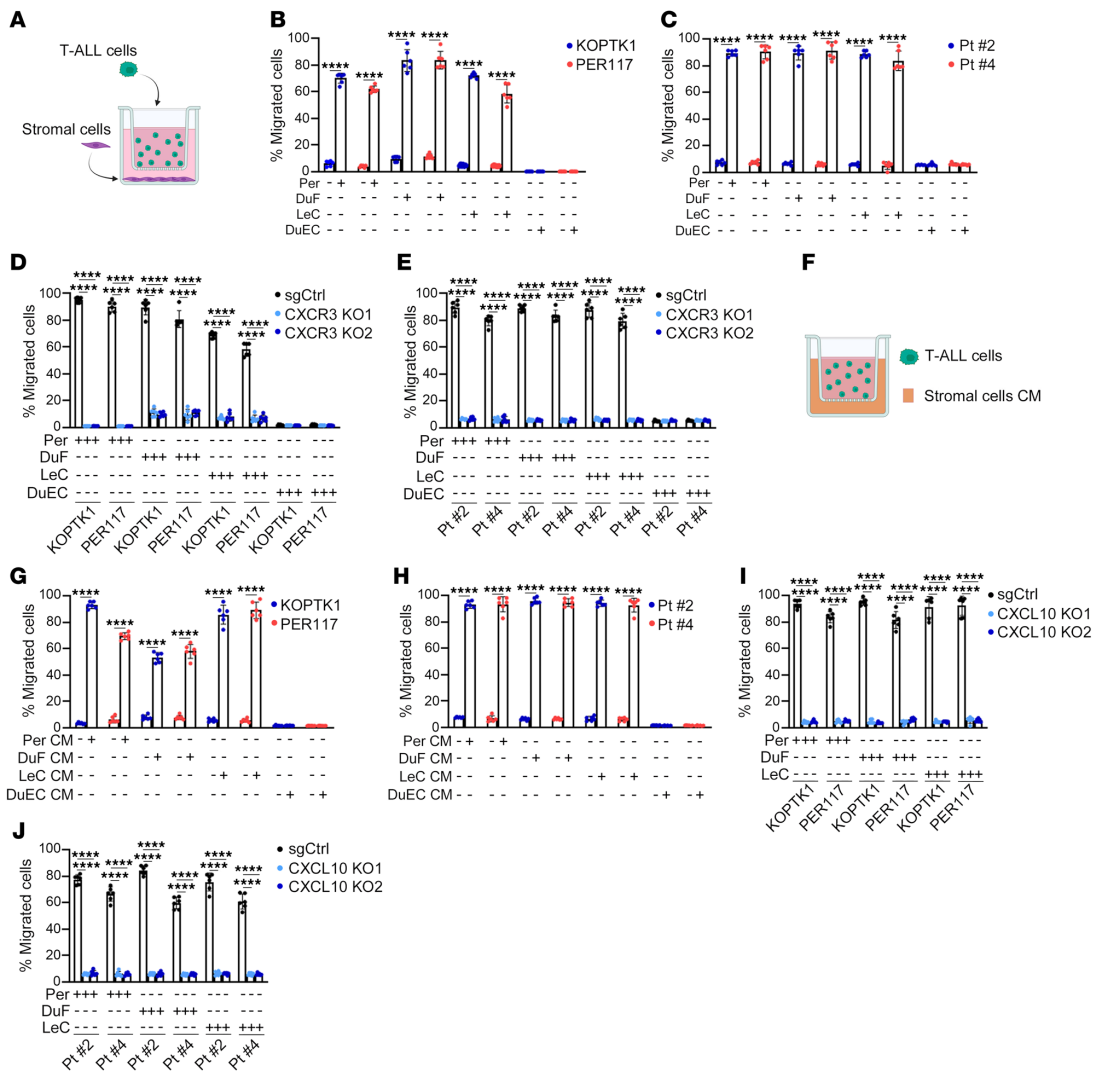
CXCL10 from fibroblasts and pericytes enhances migration of T-ALL cells. (**A**) A representation of T-ALL: T-ALL cell migration to the meningeal stroma. (**B**) Migration of T-ALL cell lines (KOPTK1, PER117) and (**C**) primary T-ALL samples (Pt #2, Pt #4) in the presence or absence of human primary meningeal stromal cells (Per, pericytes; DuF, dural fibroblasts; LeC, leptomeningeal cells, DuEC, dural endothelial cells) (6 h, 3 μm). The effect of CRISPR/Cas9-mediated knockout of *CXCR3* (CXCR3 KO1, CXCR3 KO2, sgRNAs targeting CXCR3; SgCtrl, negative control) in (**D**) T-ALL cell lines and (**E**) primary T-ALL cells on migration of leukemic cells towards meningeal stromal cells (6 h, 3 μm). (**F**) A scheme: T-ALL cell migration to conditioned medium (CM, 48 h) from meningeal stroma. The migration of (**G**) T-ALL cell lines and (**H**) primary T-ALL cells towards meningeal stromal cells CM (6 h, 3 μm). Fresh medium for meningeal stromal cells was used as a control. The migration of (**I**) T-ALL cell lines and (**J**) primary T-ALL cells upon *CXCL10* knockout (CXCL10 KO1, CXCL10 KO2, sgRNAs targeting CXCL10; SgCtrl, negative control) in human primary meningeal stromal cells (6 h, 3 μm). (**A**–**J**) Mean ± SD from 3 independent experiments performed in duplicate. (**B**, **C**, **G**, and **H**) Unpaired t test with Holm-Šidák correction for multiple testing. **(D**, **E**, **I**, and **J)** One-way ANOVA with Tukey’s multiple comparison correction; ****P < 0.0001.

We next studied the effect of meningeal stromal cells on CXCR3-mediated T-ALL cell migration. We found that T-ALL cell lines (KOPTK1, PER117) and primary cells (Pt #2, Pt #4) migrated to primary human LeC, Per, and DuF (Figure 6, A–C). On the contrary, there was no evidence for migration of leukemic cells to DuEC (Figure 6, B and C). Treatment of T-ALL cells with a CXCR3 antagonist or CRISPR/Cas9-mediated knockout of *CXCR3* in T-ALL cells reduced leukemic cell migration to the meningeal stromal cells compared with controls (Figure 6, D and E, and Supplemental Figure 6, A and B). We next investigated if T-ALL cell migration is driven by factors secreted by the meningeal stroma. T-ALL cells were tested for their migratory activity toward conditioned medium (CM) derived from meningeal stromal cells, in comparison with fresh culture medium. We observed an increase in migration of T-ALL cell lines and primary samples in the presence of CM from LeC, Per, and DuF compared with fresh culture media (Figure 6, F–H). As expected, DuEC CM did not induce leukemic cell migration. Notably, a CXCL10 neutralizing antibody inhibited T-ALL cell migration to both meningeal stroma cells and stromal CM (Supplemental Figure 6, C–E). To further delineate the effect of stroma-derived CXCL10 on T-ALL cell migration, we knocked out *CXCL10* in primary human LeC, Per, and DuF (Supplemental Figure 6, F and G). We observed reduced migration of T-ALL cell lines and patient samples to the tested stromal cells upon *CXCL10* deletion compared with control cells (Figure 6, I and J). Moreover, T-ALL cells did not migrate to CM from LeC, Per, and DuF carrying *CXCL10* knockout compared with CM from control stromal cells, further supporting the role of stroma-derived CXCL10 in T-ALL migration (Supplemental Figure 6H). Lastly, to model the meningeal microenvironment in vitro, we established tertiary and quaternary coculture systems to test whether T-ALL cells transmigrate to DuF and LeC across either a DuEC monolayer or a DuEC/pericyte bilayer. We confirmed that T-ALL cells can migrate across both endothelial and endothelial/pericyte-enriched barriers in these engineered coculture systems (Supplemental Figure 6, I–N). Together, these results show that CXCL10 secreted from meningeal stromal cells regulates migration of CXCR3-expressing T-ALL cells.

### CXCL10-CXCR3 signaling promotes T-ALL cell adhesion to meningeal stromal cells.

**Figure 7:**
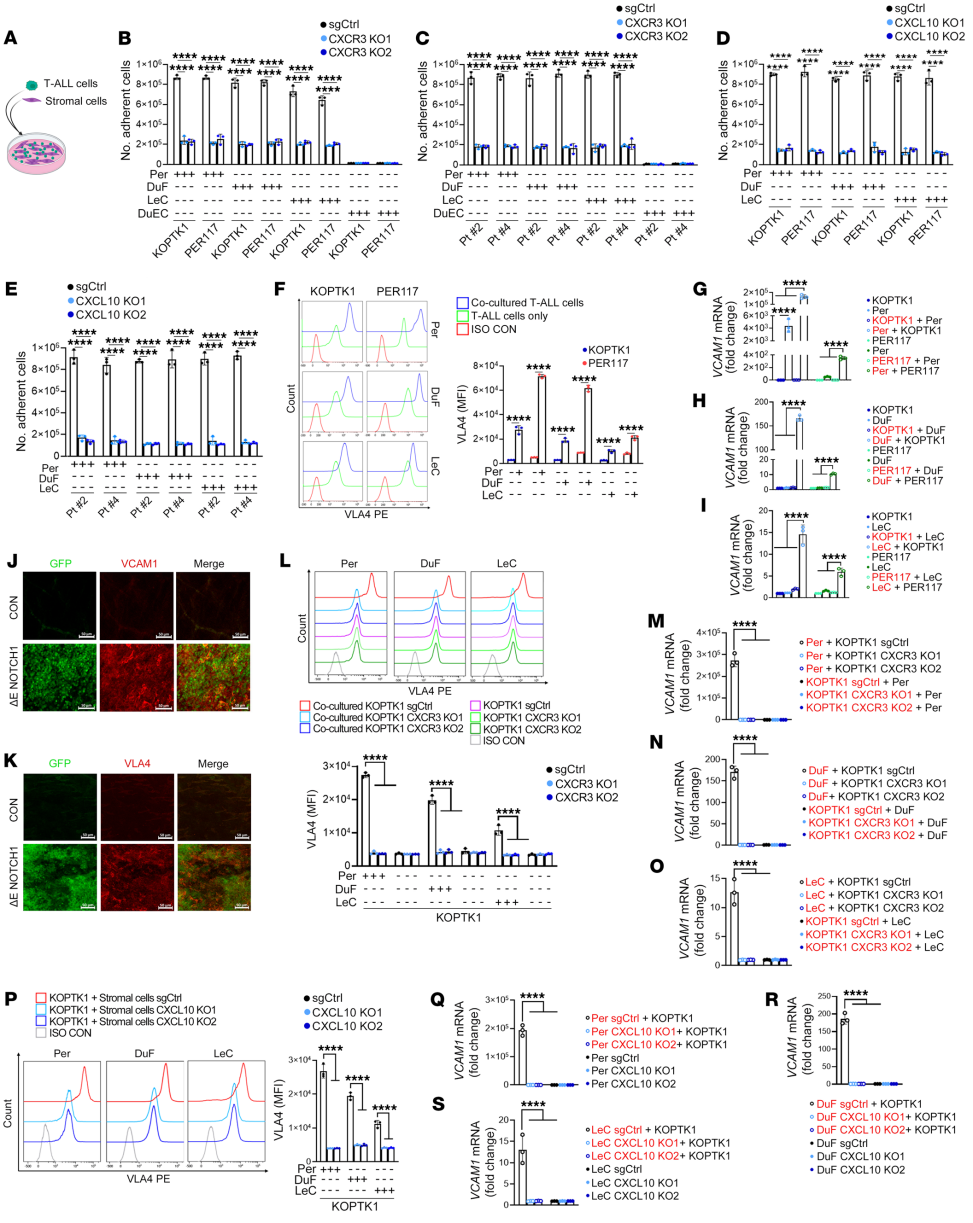
CXCL10-CXCR3 regulates T-ALL-meningeal stroma cell-cell adhesion. (**A**) A graphic of T-ALL and meningeal stromal cell coculture. The effect of CRISPR/Cas9-mediated knockout of *CXCR3* (CXCR3 KO1, CXCR3 KO2, sgRNAs targeting CXCR3; SgCtrl, negative control) in (**B**) T-ALL cell lines (KOPTK1, PER117), (**C**) primary T-ALL cells (Pt #2, Pt #4), and (**D** and **E**) *CXCL10* knockout (CXCL10 KO1, CXCL10 KO2, sgRNAs targeting CXCL10; SgCtrl, negative control) in primary human meningeal stromal cells (Per, pericytes; DuF, dural fibroblasts; LeC, leptomeningeal cells, DuEC, dural endothelial cells) on leukemic-stromal cell-cell adhesion (6h). (**F**) VLA-4 on KOPTK1 and PER117 cocultured with meningeal stroma (Per, DuF, LeC). Representative histograms (left); MFI ± SD, 3 separate experiments (right). (**G**–**I**), *VCAM1* mRNA in T-ALL cells cultured alone (KOPTK1, PER117), stromal cells cultured alone (Per, DuF, LeC), cocultured T-ALL cells (red font), or cocultured stromal cells (red font) (6h). Immunolabeling of whole meninges from T-ALL (ΔE-NOTCH1) and negative control (CON) mice; (**J**) VCAM1 (red), (**K**) VLA4 (red), GFP-expressing T-ALL cells (green) (*n* = 3/group). Scale bars: 50 μm. (**L**) VLA-4 in KOPTK1 carrying *CXCR3* knockout (CXCR3 KO1, CXCR3 KO2,) and control cells (SgCtrl) cultured with/without meningeal stromal cells. Representative histograms (top); MFI ± SD, 3 separate experiments (bottom). (**M**–**O**) *VCAM1* mRNA in mKOPTK1 (with/without CXCR3 knockout) cocultured with meningeal stromal cells. The cells were sorted after 6 hours of coincubation, followed by *VCAM1* expression in the specified cells (red font). (**P**) VLA-4 in KOPTK1 cells cocultured with stromal cells (Per, DuF, LeC) (6h) upon CXCL10 knockout. Representative histograms (left); MFI ± SD, 3 separate experiments (right). (**Q**–**S**) *VCAM1* mRNA in meningeal stromal cells (with/without CXCL10 knockout) cultured alone or cocultured (red font) with KOPTK1 cells. **(B**–**I** and **L**–**S**) Mean ± SD, 3 separate experiments. Two-way ANOVA with Tukey’s multiple comparison test; ****P < 0.0001.

We next hypothesized that the CXCR3-CXCL10 signaling axis contributes to leukemic cell retention in the meningeal microenvironment. First, we confirmed the adhesion of T-ALL cells to LeC, Per, and DuF in both standard and multicell coculture systems, whereas no adhesion was observed to DuEC (Supplemental Figure 7, A–D). To investigate the role of CXCR3 in leukemic-meningeal cell-cell adhesion, we performed *CXCR3* knockout in T-ALL cell lines (KOPTK1 and PER117) and primary samples (Pt #2 and Pt #4) followed by cell-cell adhesion analyses. *CXCR3* deletion reduced the adhesion of T-ALL cell lines and primary samples to LeC, Per, and DuF (Figure 7, A–C). Conversely, knockout of *CXCL10* in LeC, Per, and DuF led to decreased adhesion of T-ALL cells to the tested stromal cells (Figure 7, D and E). Accordingly, the adhesion of T-ALL cells was reduced upon treatment with a CXCR3 antagonist or CXCL10 neutralizing antibody compared with untreated or control leukemic cells (Supplemental Figure 7, E and F). Functionally, coculturing T-ALL cells with meningeal stromal cells resulted in increased expression of VLA-4 on KOPTK1 and PER117 cells (Figure 7F) concomitant with elevated levels of *VCAM-1* in LeC, Per, and DuF (Figure 7, G–I). Additionally, the expression of both *VCAM-1* and VLA-4 was increased in the meninges of *ΔE NOTCH1* T-ALL mice compared with control animals (Figure 7, J and K). Knockout or pharmacological inhibition of CXCR3 in T-ALL cells (Figure 7, L–O, and Supplemental Figure 7, G–M) or CXCL10 in LeC, Per, and DuF (Figure 7, P–S, and Supplemental Figure 7, N–T) reduced the expression of VLA-4 and *VCAM-1* in T-ALL and stromal cells, respectively, further supporting the role of CXCL10-CXCR3 in regulating T-ALL adhesion to meningeal stroma. Taken together, our data demonstrate that CXCL10-CXCR3 signaling enhances cell-cell adhesion between T-ALL and meningeal stromal cells through VLA-4/VCAM-1.

### Leukemia-derived proinflammatory cytokines induce CXCL10 in meningeal stromal cells.

**Figure 8:**
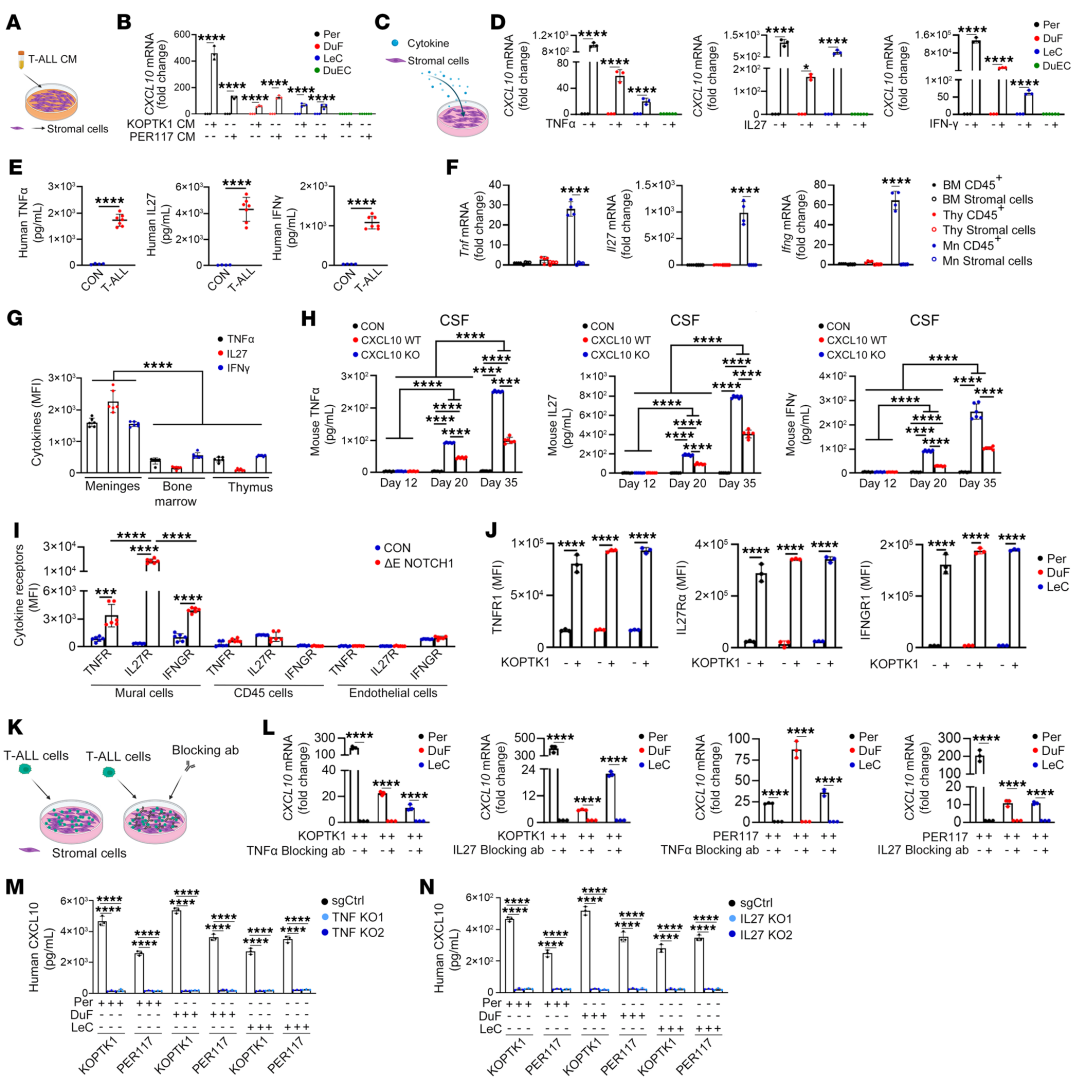
Leukemia-derived cytokines induce CXCL10 in meningeal stromal cells. (**A**) Stromal cells incubated with T-ALL conditioned medium (CM). (**B**) *CXCL10* mRNA in human meningeal stroma (Per, pericytes; DuF, dural fibroblasts; LeC, leptomeningeal cells, DuEC, dural endothelial cells) exposed to T-ALL CM (6h). (**C**) Cytokine-stimulated stroma. (**D**) *CXCL10* mRNA in meningeal stroma after TNF-α (10 ng/mL), IL-27 (100 ng/mL), and IFN-γ (10 ng/mL) stimulation (1h). (**E**) TNF-α, IL-27, and IFN-γ in CSF from patients with T-ALL (*n* = 7) and healthy controls (*n* = 4). (**F**) Cytokine mRNA expression in hematopoietic (CD45^+^) and stromal (CD45^–^) cells from the BM, thymus (Thy), and meninges (Mn) of *ΔE-NOTCH1* T-ALL mice (*n* = 4). (**G**) Intracellular TNF-α, IL-27, and IFN-γ in *ΔE-NOTCH1* T-ALL cells in the meninges, BM, and thymus (MFI ± SD, *n* = 6/group). (**H**) TNF-α, IL-27, and IFN-γ in CSF of leukemic mice (CXCL10 WT and CXCL10 KO) and nonleukemic controls (CON) (*n* = 6/group). (**I**) TNFR1, IL-27R-α, and IFNGR1 on mural (fibroblasts and pericytes; CD45^–^, CD31^–^, CD13^+^), hematopoietic (CD45^+^), and endothelial cells (CD45^–^, CD31^+^) in the meninges of T-ALL (ΔE NOTCH1) and control (CON) mice (*n* = 6/group). (**J**) TNFR1, IL-27R-α, and IFNGR1 on meningeal stroma cocultured with KOPTK1. MFI ± SD, 3 separate experiments. (**K**) Coculture with blocking antibodies. (**L**) *CXCL10* mRNA in meningeal stroma pretreated with TNF-α (0.5 μg) or IL-27 (0.5 μg) blocking antibody (1h) and cocultured with T-ALL cells (6h). (**M** and **N**) CRISPR/Cas9-deletion of *TNF* and *IL-27* in KOPTK1 and PER117 (TNF-α KO1/KO2, sgRNAs targeting *TNF*; IL-27 KO1/KO2, sgRNAs targeting *IL-27*; SgCtrl, negative control). CXCL10 in the medium after 6 hours of T-ALL-stromal cell coculture. (**B**, **D**, **L**–**N**) Mean ± SD, 3 separate experiments. (**E**) Unpaired *t* test with Holm-Šidák and (**B**, **D**, **F**–**J** and **L**–**N**) two-way ANOVA with Tukey’s multiple comparison tests; ****P < 0.0001.

We aimed to understand how CXCL10 is induced during leukemic colonization of the meninges. Given that T-ALL cells secrete proinflammatory cytokines (70), we hypothesized that factors derived from T-ALL induce CXCL10 in the meningeal microenvironment. To test this hypothesis, we incubated meningeal stromal cells in CM from T-ALL cell lines, KOPTK1, and PER117. The CM from T-ALL cells induced an increase in *CXCL10* expression in Per, LeC, and DuF, which corresponded with increased CXCL10 secretion, while DuEC showed no response (Figure 8, A and B, and Supplemental Figure 8A). This suggests that leukemic cells deliver specific factors that induce CXCL10 in subsets of meningeal stromal cells. Next, we analyzed the CM from T-ALL cells incubated with or without LeC, Per, DuF, and DuEC. We observed an increase in the levels of IFN-γ, TNF-α, IL-7, IL-27, and PDGF-α in the CM from the tested cocultures, except for DuEC cocultures and compared with CM from T-ALL and meningeal cells cultured alone (Supplemental Figure 8B). Subsequently, we demonstrated that stimulation with recombinant IFN-γ, TNF-α, and IL-27 induced *CXCL10* expression in human meningeal LeC, Per, and DuF compared with IL7- and PDGF-α–treated cells (Figure 8, C and D, and Supplemental Figure 8C). Interestingly, the levels of TNF-α, IFN-γ, and IL-27 were elevated in CSF samples from T-ALL patients (*n* = 7) compared with normal CSF samples (*n* = 4) (Figure 8E, Supplemental Table 2). Consistently, levels of *Ifng*, *Tnf*, and *IL-27* mRNA were increased in immune (CD45+) cells infiltrating the meninges, but not in meningeal stromal cells (CD45–) of *ΔE-NOTCH1* mice (Figure 8F). Furthermore, IFN-γ, TNF-α, and IL-27 expression was elevated in T-ALL cells (GFP^+^/CD45^+^) infiltrating the meninges, but not in leukemic cells from the the BM or thymus of T-ALL mice, suggesting a unique inflammatory crosstalk between leukemic cells and the meningeal microenvironment (Figure 8G). Analysis of CSF and blood serum samples from control and *ΔE-NOTCH1* mice revealed a gradual increase in cytokine levels during T-ALL progression (Figure 8H and Supplemental Figure 8D). To investigate how the meningeal microenvironment responds to T-ALL–derived cytokines, we evaluated the expression of TNFR1, IL-27R-α, and IFNGR1 on meningeal stromal and immune cells, and confirmed elevated receptor expression on mural (fibroblasts and pericytes) meningeal cells in *ΔE-NOTCH1* mice (Figure 8I). Accordingly, coincubation of T-ALL cell lines with human LeC, Per and DuF induced TNFR, IL-27R, IFNGR on the tested stromal cells (Figure 8J and Supplemental Figure 8E). To further dissect the mechanism by which T-ALL–derived cytokines regulate CXCL10 expression in the meningeal stroma, we performed coculture assays in the presence or absence of cytokine-specific blocking antibodies. Since IFN-γ is a well-known inducer of CXCL10 (71, 72), we focused on the roles played by TNF-α and IL-27, which are less characterized regulators of CXCL10 signaling. Pharmacological inhibition of TNF-α and IL-27 with neutralizing antibodies reduced *CXCL10* expression in Per, DuF, and LeC, concomitant with a decrease in T-ALL migration to the meningeal stromal cells (Figure 8, K and L, and Supplemental Figure 8, F–I). These observations were accompanied by a decrease in CXCL10 secretion, as measured by ELISA (Supplemental Figure 8, J and K). In line with this, CRISPR/Cas9-mediated knockout of *TNF* and *IL-27* in KOPTK1 and PER117 cells resulted in reduced CXCL10 secretion concomitant with a decrease in *CXCL10* expression in stromal cells (Figure 8, M and N, and Supplemental Figure 8, L–O). Consequently, the migration of T-ALL cells towards Per, DuF, and LeC was also inhibited (Supplemental Figure 8, P and Q). Collectively, these results indicate that T-ALL cells secrete proinflammatory cytokines, which in turn, activate CXCL10 expression.

## Discussion

It has been long understood that CNS disease negatively impacts T-ALL treatment outcomes (6, 8). Here, we identify the CXCR3-CXCL10 signaling axis as a critical regulator of T-ALL dissemination and retention within the meningeal niche.

We demonstrated that CXCR3 plays dual and context-dependent roles in T-ALL biology. In the absence of ligand, CXCR3 stabilizes active β-catenin, promoting leukemic proliferation, whereas CXCL10 engagement triggers β-catenin degradation and facilitates T-ALL cell migration. These findings support a model in which β-catenin acts as a molecular switch between proliferative and migratory programs in T-ALL. Additionally, CXCR3 isoforms may engage in ligand-independent or atypical signaling, influenced by receptor localization, dimerization, or crosstalk, suggesting broader, context-dependent functions beyond canonical ligand engagement (64, 65, 73). This signaling versatility may enable leukemic cells to dynamically adapt to changing microenvironmental cues during disease progression.

Moreover, our findings demonstrate that CXCR3 promotes T-ALL cell migration, with a marked preference for CXCL10 over CXCL9 and CXCL11, suggesting selective responsiveness to a CXCL10 gradient. Notably, pharmacological inhibition with the CXCR3 antagonist AMG487 recapitulated the effects of genetic loss of CXCR3, reducing T-ALL cell migration to CXCL10, while forced CXCR3 expression was sufficient to drive leukemic cells to the meninges. These results underscore CXCR3’s potential role in mediating T-ALL infiltration of the meningeal niche. This is consistent with CXCR3’s established role in guiding T cell trafficking (50, 53, 55) and with CXCR3^+^ T cells being recruited to CXCL10-rich tumor sites to augment antitumor immunity (74–77). Our observations support the notion that T-ALL exploits normal T cell function to accelerate disease progression and dissemination. CXCR3 upregulation has also been linked to IL15-mediated B-ALL cell migration (21) and has been observed in ALL patient samples with CNS disease or relapse (21, 31). While no association between CXCR3 expression and CNS status was found in our study, this likely reflects the limited sensitivity of current cytospin-based diagnostic methods (78), underscoring the need for robust tools and improved biomarkers to accurately capture CNS involvement.

Our findings further highlight CXCR3 as a potential therapeutic target in T-ALL, consistent with its established role in solid tumor progression and metastasis (47, 48, 56–58). Although CXCR3 has been linked to tumor dissemination in multiple cancers, its impact appears to be context- and tumor-dependent. The spatial distribution of CXCR3 expression provides additional insight into CXCR3 function. We found the highest levels of CXCR3 in leukemic cells infiltrating the meninges, thymus, and BM, suggesting a role in both migration and adaptation to specific microenvironments. Similar compartmentalized expression patterns have been reported in solid tumors, where CXCR3 is enriched in metastatic foci compared with primary sites (57, 58). Elevated CXCR3 expression in primary T-ALL samples compared with normal thymic cells further strengthens its therapeutic potential, and strategies targeting CXCR3 have already been explored in several cancers and inflammatory diseases (58, 79–82). While additional studies are needed to elucidate the mechanistic basis of CXCR3-mediated T-ALL cell migration and meningeal infiltration, our findings underscore CXCR3’s role in T-ALL dissemination and highlight it as a promising therapeutic target, warranting further evaluation of CXCR3-directed therapies in both preclinical and clinical settings.

The increased CXCR3 levels observed in ΔE-NOTCH1–driven T-ALL point to a role for NOTCH1 signaling in CXCR3 regulation. Consistent with prior genome-wide studies linking CXCR3 signaling to NOTCH1-driven T-ALL (68), we identified a role for USP7 in stabilizing CXCR3 and demonstrated a specific USP7-NOTCH1 interaction that contributes to its transcriptional regulation (67, 68). Importantly, USP7 interacts with the NOTCH1 ankyrin domain, which remains intact in both WT and mutant proteins (68), suggesting that USP7 binding and regulation of CXCR3 occur irrespective of NOTCH1 mutational status. This implies that the USP7-NOTCH1-CXCR3 axis may be broadly relevant across molecular subtypes of T-ALL, warranting further investigation.

CXCL10 was elevated in BM, blood, and CSF of ΔE-*NOTCH1* T-ALL mice compared with controls, suggesting a localized inflammatory response within distinct microenvironments. Reduced meningeal infiltration in CXCL10-knockout mice points to a specific role of CXCL10 in leukemic colonization of this niche. Elevated CXCL10 levels have been reported in the CSF of ALL patients (21, 31) and have also been linked to advanced disease stage, metastasis, and poor prognosis in metastatic solid tumors (47, 49, 58, 83–86). Although CXCL10, CXCL9, and CXCL11 share roles in immune cell recruitment, they often display distinct, nonredundant and context-specific functions across various tumors and inflammatory conditions (41, 71). Consistent with this, our findings support a unique role for CXCL10 in guiding T-ALL to the meninges, highlighting CXCL10 as a selective and potentially actionable therapeutic target in CNS disease.

Furthermore, we observed an enhanced inflammatory response in the CSF of leukemia-bearing mice characterized by elevated CXCL10 levels and reduced proliferation of leukemic cells in the meninges compared with the BM. Moreover, CXCL10 loss did not increase T-ALL cell death, supporting its role as a migratory cue rather than a survival factor. We propose that high levels of CXCL10 in the CSF establish a chemotactic gradient that attracts T-ALL cells to the meninges. Concurrently, the inflammatory response within the CSF may modulate the meningeal microenvironment, creating a sanctuary site for T-ALL cell survival. A limitation of our study is that CXCL10-KO mice may have altered immune cell trafficking, potentially influencing disease burden. While our findings underscore the importance of the CXCR3-CXCL10 axis in T-ALL, further work is needed to define how immune cells contribute to leukemic progression within the meningeal niche.

Our findings also identify fibroblasts and pericytes as the primary sources of CXCL10 in T-ALL–infiltrated meninges, suggesting that meningeal stromal cells respond to leukemic cues and guide leukemic migration. Fibroblasts and pericytes are key players in solid tumor development and growth at metastatic sites (58, 87, 88). For instance, CXCR3-expressing breast cancer cells induced CXCL9/10 in lung metastasis-associated fibroblasts (58). Elegant studies by DeSisto et al. (15) showed that meningeal fibroblasts constitutively express CXCL12, supporting previous reports that CXCL12 promotes homing of T-ALL cells to the CNS and BM (33, 35). Subsequent studies found that dural stromal cells expressed an abundance of CXCL12, which mediated homeostatic T cell recruitment to dural sinuses (16). This raises the intriguing question of the interplay between constitutively expressed CXCL12 and inflammation-induced CXCL10 in facilitating T-ALL colonization of the meningeal niche. Strikingly, we also found that meningeal pericytes increase CXCL10 production in response to T-ALL. Pericytes control leukocyte extravasation into the brain and meninges upon activation by proinflammatory cytokines (72, 89–91) and produce several proinflammatory chemokines, including CXCL10 (90, 92). Interestingly, the CNS has the highest pericyte coverage of any tissue (93, 94), and abnormal pericyte coverage of tumor blood vessels has been linked to increased metastatic potential across various cancer types (87). These observations reveal a reciprocal interplay between T-ALL cells and the meningeal stromal cells, highlighting the impact of T-ALL cells on CXCL10 production by meningeal fibroblasts and pericytes.

Building on this, we showed that T-ALL–derived IFN-γ, TNF-α, and IL-27 induce CXCL10 expression in the meningeal microenvironment, resulting in increased permissiveness of the meninges to T-ALL. While the role of IFN-γ in inducing CXCL10 during inflammation is well documented (42, 71), the roles of IL-27 and TNF-α are less well understood (95–97). T-ALL cells produce several autocrine and paracrine cytokines that differentially regulate leukemia survival and proliferation (70). Interestingly, elevated levels of TNF-α were associated with leukemia progression and extramedullary infiltration in AML and ALL (98, 99). Intriguingly, IL-27, which displays pleiotropic functions in cancer (100), was shown to inhibit AML and B-ALL progression in preclinical models (101, 102). Although we did not directly investigate the upstream mechanisms regulating CXCL10 induction in this study, our findings of reduced IFN-γ, TNF-α, and IL-27 expression in leukemia-bearing CXCL10-deficient mice underscore the need for further mechanistic studies. In normal T cells, CXCR3 signaling regulates cytokine expression, particularly IFN-γ and TNF-α, and indirectly induces CXCL10 in stromal cells through these cytokines (103). It is plausible to speculate that similar mechanisms may operate in T-ALL, whereby CXCR3-positive leukemic cells amplify cytokine production and promote stromal CXCL10 expression, thereby reinforcing leukemic cell recruitment and retention within the meninges. While this has not been investigated in T-ALL, such studies are warranted to define upstream regulators and to determine whether IFN-γ, TNF-α, and IL-27 act synergistically or independently to drive CXCL10 expression and leukemic cell recruitment.

In this study, we also identified a functional link between CXCR3-CXCL10 and enhanced T-ALL cell adhesion to meningeal stroma. Specifically, *CXCR3* upregulation increased VLA-4 integrin expression, augmenting T-ALL cell adhesion to VCAM1-expressing fibroblasts and pericytes. Moreover, treatment with a CXCR3 antagonist or a CXCL10-neutralizing antibody reduced T-ALL adhesion, underscoring the role of CXCL10-CXCR3 signaling in mediating cell-cell adhesion. These observations could inform strategies aimed at disrupting leukemic cell retention within the meningeal microenvironment. Evidence that leukemic cells require stromal cell contact for survival (38, 39, 104) further justifies targeting CXCL10-mediated signaling in the CSF. Approaches to modulate CXCL10 levels within the CNS have already been explored in the context of neuroinflammatory diseases (42, 105). Although targeting CXCL10 in the CSF for leukemia treatment constitutes an ongoing research focus, it potentially opens new avenues for future therapeutic interventions.

In summary, this study uncovers the reciprocal role of CXCR3-CXCL10 signaling that orchestrates T-ALL progression and meningeal colonization. Our results underscore the significance of meningeal stromal cells and stroma-derived CXCL10 in regulating the neurotropism and retention of CXCR3-expressing T-ALL. We highlight the impact of T-ALL–secreted proinflammatory cytokines in inducing CXCL10 in the meningeal fibroblasts and pericytes, thereby facilitating leukemic cell meningeal colonization. These insights illuminate mechanisms of T-ALL neurotropism and identify multiple potential therapeutic targets, including CXCR3, CXCL10, and downstream cytokine pathways, that could be exploited to disrupt leukemic trafficking and retention. Our ongoing studies aimed at pharmacologically targeting this axis, including the use of CXCR3 antagonists such as AMG487 or CXCL10-neutralizing antibodies like eldelumab, may open new avenues for systemic and CNS-directed therapies beyond conventional cytotoxic approaches.

## Methods

### Sex as a biological variable.

Our study exclusively examined male mice. It is unknown whether the findings are relevant for female mice.

For further methods, see Supplemental Methods.

### Study approval.

Deidentified primary patient samples were obtained from the Children’s Oncology Group study ALL0434 and the University of New Mexico (IRB #16-246 and #03-183), and the University of Alabama at Birmingham (IRB-300009609, IRB-160422003). All patients or their parents or guardians provided written, informed consent in accordance with the Declaration of Helsinki and local institutional guidelines. Peripheral blood was collected from healthy donors with informed consent and ethical approval from the Swansea University Medical School Research Ethics Committee (SUMSRESC; 2022-0029). The animal experiments were approved by the ethical committees on animal welfare at the University of New Mexico (19-30020-HSC) and the University of Alabama at Birmingham (IACUC-22544, IACUC-22519).

### Statistics.

The statistical analyses were carried out using GraphPad Prism 10. Data are presented as mean ± SD unless otherwise indicated. Comparisons between 2 groups were performed using 2-tailed unpaired Student’s *t* test. Comparisons among multiple groups were performed using 1-way ANOVA or 2-way ANOVA, as appropriate, followed by Tukey’s or Šidák’s multiple-comparison post hoc tests, as specified in the figure legends. For experiments involving 2 independent variables, 2-way ANOVA was used. Survival curves were analyzed using the log-rank (Mantel-Cox) test. The number of biological replicates (*n*) and the specific statistical tests used are indicated in the corresponding figure legends. Statistical significance was determined at *P* < 0.05. 

### Data availability.

The data generated in this study are provided in the Supporting Data Values file accompanying this paper.

## Author contributions

NDS and KMW designed the studies. NDS, EK, WO, SP, MN, QJ, BLM, HK, KEZ and NJ and KMW gathered and compiled data. PN provided critical reagents. TT analyzed normal thymocytes by flow cytometry. CCB, JW, CH, SSW, MLL, and SPH provided patient samples and clinical data. The data was analyzed and interpreted by NDS, WO, HK, EFC, TYV, NJ, PN, and KMW. NDS and KMW wrote the manuscript. KMW originated and supervised the project. All authors reviewed and approved the manuscript.

## Funding support

This work is the result of NIH funding, in whole or in part, and is subject to the NIH Public Access Policy. Through acceptance of this federal funding, the NIH has been given a right to make the work publicly available in PubMed Central.

- The National Cancer Institute (NCI) grant R01 CA237165 and R01 CA282701 (to KMW).
- O’Neal Invest (to KMW).
- Children’s Oncology Group Specimen Banking (U24 CA114766), (AALL15B1-Q, to KMW).
- NCI Cancer Center Support Grant P30 CA013148 (UAB) and P30 CA118100 (UNM).
- Dixon Foundation (to CCB).
- Research Foundation Flanders Ghent University and Flanders interuniversity consortium grant (to PN)..
- Cancer Research Institute Ghent (CRIG) partnership grant (FWO G0F4721N and BOF.IBO.2023.0006.02, to PN).
- Little Princess Trust in partnership with Children’s Cancer and Leukaemia Group (CCLG) grant (CCLGA) (2020 24, to CH; 2021 05 and 2024 08, to NJ).
- Cancer Research UK Programme Foundation Award (DRCPFA-Nov21\100001, to CH).
- American Cancer Society Research Scholar (to KMW).

## Supplementary Material

Supplemental data

Unedited blot and gel images

Supporting data values

## Version 1 10/23/2025

In-Press Preview

## Version 2 01/16/2026

Electronic publication
