# Supplementary material for: T cell acute lymphoblastic leukemia exploits a neural proinflammatory pathway to colonize the meninges
Source: J Clin Invest. 2025 Oct 23;136(2):e188888. doi: 10.1172/JCI188888 (PMC12807478; doi:10.1172/JCI188888)
Supplement: Supplemental data [file jci-136-188888-s231.pdf]

## Supplemental Material and Methods

**Cell lines and Primary Cells*.*** Unless stated otherwise, all cells were cultured in a complete medium comprising RPMI-1640 medium supplemented with 10% or 20% FBS, 2mM L-glutamine, and 100 U/ml penicillin G-streptomycin at 5% CO2 at 37°C. Peripheral blood mononuclear cells (PBMCs) were isolated via density gradient centrifugation using Lymphoprep™ (STEMCELL Technologies). Primary T-ALL cells were cultured as described previously [1]. Human T-ALL cell lines were obtained from ATCC or DSMZ. The manufacturer's instructions were followed to grow human umbilical cord vascular endothelial cells (HUVEC) (ATCC, Manassas, VA), primary human leptomeningeal pericytes (Per), primary human dural microvascular endothelial cells (DuEC), primary human meningeal cells (LeC), and primary human dural fibroblasts (DuF) (ScienCell Research Laboratories, Carlsbad, CA, USA).

**Lentivirus/Retrovirus production and transduction.** For the *CXCR3, CXCL10, TNF* and *IL27* CRISPR/Cas9 knockout, two independent sgRNAs that target *CXCR3* (CXCR3 KO1 and CXCR3 KO2), *CXCL10* (CXCR10 KO1 and CXCR10 KO2), *TNF* (TNFα KO1 and TNFα KO2) or *IL27* (IL27 KO1 and IL27 KO2) were used in a lentiviral vector along with an appropriate scrambled control (sgCtrl). 293FT cells were transfected with lentivirus recombinant expression plasmids and packaging vectors using Lipofectamine 2000 (Life Sciences, Carlsbad, CA, USA). After 48 h, the culture supernatants were collected and titrated. At a multiplicity of infection (MOI) of 10 viral particles per cell, lentivirus particles were mixed with 8 µg/ml polybrene (Sigma-Aldrich, St. Louis, MO, USA) and added to T-ALL cell lines or primary samples, followed by centrifugation at 1150 x g for 90 minutes at 32°C. Following infection, transduced cells were maintained under puromycin selection for 3 weeks.

CXCR3 TV1 (GFP) (pLenti-C-mGFP-P2A-Puro), CXCR2 TV2 (RFP) (pLenti-mRFP-P2A-Puro), and CXCR3 TV1 + TV2 along with appropriate controls, were transduced into DND41 cells with

lentivirus particles. Following infection, the transduced cells were selected and cultured under puromycin selection for approx. 3 weeks. Lentivirus-infected cells were analyzed for GFP+/RFP+ using FACSymphony (BD Bioscience), followed by FACS-sorting.

The pQflag-USP7 WT puroR and pQflag-USP7 CS puroR plasmids expressing wild-type and catalytically inactive USP7 proteins were provided by Addgene. Each plasmid (10 µg) was transfected into the Phoenix packaging cell line using Lipofectamine 2000. KOPTK1 cells were spinoculated in retroviral supernatants supplemented with 4 µg/ml polybrene and 20% RPMI 1640, followed by selection with puromycin. KOPTK1 cells expressing shRNA USP7 (shUSP7 1 and shUSP7 2) and scrambled negative control shRNA (shNC), were retrovirally transduced with (USP7 WT and USP7 CS), if applicable.

For bioluminescence in vivo imaging, transduced KOPTK1 cells expressing CXCR3 KO1, CXCR3 KO2, and sgCtrl were co-transduced with pLenti-EF1a (Red-Luciferase)-Rsv (RFP-Blasticidin) lentiviral particles (GenTarget Inc, San Diego, CA, USA). The transduced cells were cultured for 3 weeks under blasticidin (3 µg/ml) selection (Gibco). Lentivirus-infected cells were examined for $RFP^+$ using the FACSAria flow cytometer, followed by FACS-sorting. All the vectors used are listed in Supplementary Table S2.

**RNA extraction.** RNA was extracted from T-ALL cells or meningeal stromal cells using the Direct-Zol™ RNA MiniPrep Kit (Zymo Research, Irvine, CA, USA) according to the manufacturer's guidelines.

**Real-time Quantitative PCR (RT-qPCR).** Reverse Transcription Reagents were used to synthesize cDNA from 1µg of DNase I treated total RNA. RT-qPCR was performed using TaqMan® on a StepOnePlus™ RealTime PCR System under standard conditions. The housekeeping gene glyceraldehyde-3-phosphate dehydrogenase (GAPDH) was used as an internal reference. TaqMan RT-qPCR was performed according to the manufacturer's

specifications. The assay identification numbers of the probes (Applied Biosystems, USA) are listed in Supplementary Table S3.

**ELISA.** The levels of mouse or human CXCL10, CXCL9, CXCL11, TNFα, IL27 and IFNγ were assessed in blood serum, BM serum and cerebrospinal fluid (CSF) of T-ALL (ΔE-NOTCH1) and control (CON) mice and/or NSG mice inoculated with transduced human T-ALL cell lines (PER117, KOPTK1, DND41), and/or CSF samples from T-ALL patients or healthy donors, using ELISA kit (R&D Systems) according to the manufacturer's instructions. For experiments involving human cells, KOPTK1 or PER117 cells were co-cultured with primary human meningeal stromal cells (Per, DuF, LeC, DuEC) for 6h, followed by cell culture medium collection. ELISA for human CXCL10 and CXCL9 (R&D Systems) was performed according to the manufacturer's instructions.

**Co-culture model.** Briefly, KOPTK1 or PER117 cells ($10^6$) were seeded on an 85% confluent primary human meningeal stromal cell layer (Per, DuF, LeC, DuEC) and maintained in 5% CO2 at 37°C for 6 h. Following incubation, the cells were collected, leukemic cells were labelled with anti-human APC-CD45+ antibody (Biosciences, San Jose, CA, USA) and FACS-sorted using the FACSAria flow cytometer. Both meningeal stromal cells (alone or co-cultured) and leukemic cells were used for RT-qPCR or flow cytometry analysis.

**Extracellular and intracellular flow staining.** Standard staining techniques were applied using specified antibodies listed in the supplementary Table S4. Intracellular staining of suspension of meningeal cells from mice was performed following permeabilization and fixation with the Fixation/Permeabilization Concentrate Kit (eBioscience). All flow cytometry analyzes were performed using the LSR Fortessa instrument (BD Biosciences). Data were analyzed using Kaluza Analysis Software (Beckmann Coulter, Brea, CA, USA) and/or BD's FlowJo. Schematic representation of the gating strategy used to identify dural meningeal stromal and immune cell subsets is shown in Supplemental Figure 9.

**Animal experiments.** NOD.Cg-$Prkdc^{scid}Il2rg^{tm1Wjl}$/SzJ (NSG) mice (6 – 8 weeks old; both sexes) were obtained from the UNM Cancer Center Animal Models Shared Resource or purchased from the Jackson Laboratory, and maintained in a pathogen-free, AAALAC-accredited facility. The remaining strains were purchased from the Jackson Laboratory. T-ALL cell lines, KOPTK1 and PER117cells were transduced with CXCR3 KO1, CXCR3 KO2, or with scrambled control sgRNA. $10^6$ transduced T-ALL cells were injected into the tail vein of each mouse. For survival analyzes, mice (n = 10 mice/group) were euthanized after showing signs of moribund disease or weight loss of 15%. For leukemia burden analyses, transduced KOPTK1 cells were injected intrafemorally into mice (3 x $10^5$ cells per mouse; n = 5 mice/group). All the mice were sacrificed at day 45. Leukemic cells were isolated from the BM of femurs, blood, meninges, spleen, liver, lungs, and testis by centrifugation in a Percoll (GE Healthcare) and Ficoll-Paque density gradient. Anti-human APC-$CD45^+$ and anti-mouse BV-421-$CD45^+$ antibodies (BD Biosciences) were used to stain and evaluate cells by flow cytometry. Data were analyzed using Beckmann Coulter's Kaluza and BD’s FlowJo.

To generate ∆E NOTCH1-induced T-ALL, bone marrow cells were isolated from femurs and tibia of B6 CD45.1 mice (males, 8 – 10 weeks old), followed by enrichment in $CD117^+$ positive cells using magnetic beads (CD117 Microbeads, mouse, Miltenyi Biotec, according to the manufacturer's guidelines). The cells were transduced with an activated form of the NOTCH1 oncogene (ΔE-NOTCH1-pMSCV-IRES-GFP) or control vector (pMSCV-IRES-GFP empty vector), which were a generous gift from Dr. Ferrando at the Columbia University, USA. A day prior the transplantation, lineage (Lin)-depleted helper cells were isolated from the bone marrow of C57BL/6J mice following the manufacturer's instruction (Lineage Cell Depletion Kit, mouse, Miltenyi Biotec). The frequency of transduced $GFP^+$ Sca-$1^+$ cells was determined using flow cytometry. Next, we pooled transduced $GFP^+$ Sca-$1^+$ cells (1 X $10^5$) and helper bone marrow cells

($2.5 \times 10^5$) were pooled together and transplanted intravenously via tail vein into lethally irradiated recipient mice (C57BL/6J, males) as described previously [1].

For the experiment with the CXCL10 knockout mice**,** bone marrow $CD117^+$ positive cells were isolated from donor mice (B6 CD45.1, males, 8 – 10 weeks old) using magnetic beads. The cells were transduced by spinoculation with retrovirus encoding activated form of the NOTCH1 oncogene (ΔE-NOTCH1-pMSCV-IRES-GFP). Transduced Sca-1+ GFP+ cells ($1 \times 10^5$) and helper bone marrow cells ($2 \times 10^5$) were combined and transplanted via intravenous injection into lethally irradiated CXCL10 KO (B6.129S4-*Cxcl10*$^{tm1Adl}$/J) and wild type B6 (C57BL/6J) recipients (8-10 weeks, males, n = 4 mice/group). Mice were perfused when moribund with PBS containing 1 USP unit/L heparin and leukemic cells were isolated from the BM of the femurs and other extramedullary organs through Ficoll-Paque density gradient separation. Anti-mouse APC-$CD4^+$ and anti-mouse BV-421-$CD8A^+$ antibodies (BD Biosciences) were used to stain and identify GFP+/CD4+CD8+ DP cells by flow cytometry. Data were analyzed using Beckmann Coulter's Kaluza and BD's FlowJo.

For the multiple timepoint experiment, BM from the femur and tibia of donor mice (B6 CD45.1 mice) was enriched for $CD117^+$ cells, which were then transduced with ΔE-NOTCH1 or CON vector. Transduced Sca-1+ GFP+ cells ($1.5 \times 10^5$) and helper bone marrow cells ($3 \times 10^5$) were intravenously injected into lethally irradiated wild type B6 (C57BL/6J) recipients (Control and ΔE-NOTCH1 group) and CXCL10 KO (B6.129S4-*Cxcl10*$^{tm1Adl}$/J) mice (18 recipient mice for each group). Leukemic cells were extracted from BM and various organs at multiple timepoints (Day 12, Day 20, and Day 35 – 6 mice per timepoint) followed by staining for CD4+CD8+, and detecting GFP+/CD4+CD8+ DP cells via flow cytometry. Data were analyzed using Beckmann Coulter's Kaluza and BD's FlowJo. Additionally, we extracted the blood serum and CSF followed by CXCL10, CXCL9, TNFα, IL27, and IFNγ expression using an ELISA kit according to the manufacturer's instructions.

For whole mount meninges isolation, mice were perfused with ice-cold PBS containing heparin (1 USP unit/L). Harvested skullcaps were further fixed in 4% PFA/PBS at 4˚C for 24 h, followed by dissection of intact meninges for downstream analyzes.

For leukemia burden analysis in CXCR3 overexpressing model (CXCT3 TV1, CXCR3 TV2 and CXCR3 TV1+TV2), NSG mice (8 mice per group) received 1 x $10^6$ transduced DND41 cells via tail vein injection. Mice were sacrificed when moribund. Leukemic cells were extracted from BM from the femur, meninges, blood, spleen, liver, lungs, and testis using Percoll (GE Healthcare) and Ficoll-Paque density gradient. Cells were stained with Zombie-Aqua, followed by staining with anti-human APC-CD45+ and anti-mouse BV-421-CD45+ antibody (BD Biosciences), and analyzed by flow cytometry.

To measure CXCL10 expression in NSG mice injected with 1 x $10^6$ KOPTK1 or PER117 cells, and BM serum, blood serum, meningeal extract, and CSF were collected on Day 1 pos-injection and when the mice became moribund. CXCL10 and CXCL9 levels were measured according to the manufacturer's instructions.

**Non-competitive homing.** For the non-competitive homing assay, CXCR3 knockout (CXCR3 KO1 or CXCR3 KO2) or scrambled control sgRNA (sgCtrl) KOPTK1 cells were labeled for 30 minutes with CellTracker™ Red CMTPX (4 μM, Invitrogen) and injected i.v. via tail vein ($10^7$ cells/mouse) into 5 NSG mice/group. All mice were sacrificed 24 h post injection. Mice were perfused and leukemic cells were isolated from the BM of femurs. Anti-human FITC-CD45+ (BD Biosciences) was used to stain and assess the levels of recovered human DsRed/FITC$^+$ cells from the BM by flow cytometry.

For the non-competitive homing assay, donor mice (B6 CD45.1, males, 8 – 10 weeks old) had their BM extracted from the femur and tibia, followed by enrichement of CD117+ were using magnetic beads. CD117+ cells were transduced with ΔE-NOTCH1 vector. Transduced Sca-1+ GFP+ cells (1 x $10^6$) and helper bone marrow cells (3 x $10^5$) were intravenously injected into

lethally irradiated wild type B6 (C57BL/6J) recipient mice 5 mice/group. All mice were sacrificed 24 h post-injection, and leukemic cells were isolated from the BM of femur and meninges. The presence of GFP+CD45+ leukemic cells was enumerated by flow cytometry.

**Bioluminescence Imaging.** Leukemic cells expressing Red Luciferase$^+$ were injected intrafemorally into NSG mice (3 x $10^5$ cells per mouse, n = 3 mice/group). Bioluminescence imaging was performed using the IVIS Spectrum in vivo Imaging System (Perkin Elmer). Mice were injected intraperitoneally with 150 mg/kg D-Luciferin (Perkin Elmer) and kept conscious for 3 minutes to enable optimal D-Luciferin circulation. Mice were anesthetized with isoflurane and placed on a heated imaging platform. 10 minutes post-injection, mice were scanned supine and prone with autoexposure to maximize signal acquisition and medium binning to optimize resolution and sensitivity. The Living Image program (Perkin Elmer) evaluated the bioluminescent signal. All photos were imported as a group and analyzed using the same parameters to compare signal intensity longitudinally at several time points. Bioluminescent signal was represented as radiance (photons/sec/cm2/steradian).

**Cell viability assay.** Cell viability was evaluated using the CellTiter 96 AQueous One Solution Cell Proliferation Assay (MTS) (Promega, Madison, WI, USA). Absorbance was measured at 490 nm with a Bio-Rad iMark Microplate Absorbance Reader.

**Apoptosis and cell cycle assays.** T-ALL cells ($10^6$) were grown in 6-well plates for 48 h. For apoptosis testing, cells were washed in binding buffer and stained with 5 µl of Annexin V conjugated to BV421 and 5 µl of 7-ADD (BD Pharmingen, San Jose, CA, USA) for 15 minutes at RT in the dark. For cell cycle analysis, cells were fixed and permeabilized in 70% ethanol for 2 hrs at -20°C, then treated for 15 minutes at RT with propidium iodide/RNase Staining Buffer (BD Pharmingen). Stained cells were examined using flow cytometry.

**Immunoblotting analysis and Antibodies.** Cells ($10^7$) were washed with ice cold PBS before and lysed in M-PER® Mammalian Protein Extraction Reagent supplemented with a HaltTM

Protease and Phosphatase Inhibitor cocktail. Cell lysates were incubated for 10 min at RT and centrifuged for 15 min at 14,000 x g (4ºC). Equal amounts of total protein were separated on a precast polyacrylamide gradient gel (5-15%) using SDS-PAGE (Bio-Rad Laboratories, Hercules, CA, USA). Proteins were transferred to the nitrocellulose membrane using the Bio-Rad Trans-Blot Turbo Transfer System. After blocking, the membrane was incubated with the primary antibody at 4°C overnight, followed by the secondary antibody at RT for 1 h. Blots were developed using a chemiluminescence (ECL) reagent, and the bands were visualized using the Bio-Rad ChemiDoc™ XRS equipped with Image Lab™ 5.0 Software. All the antibodies used for immunoblotting are listed in Supplementary Table S5.

**Subcellular fractions.** Subcellular fractions were isolated using the Cell Fractionation Kit (Cell Signaling Technologies) according to the manufacturer's protocol. Enrichment of cytoplasmic, membrane, and nuclear proteins was confirmed by western blotting.

**Migration Assay.** Cell migration was tested using the QCM Chemotaxis Cell Migration Assay kit (3 µm) (Millipore Sigma, St. Louis, MO, USA). T-ALL cells were suspended in serum-free RPMI-1640 and loaded into the upper chambers in the transwell system (0.3 x $10^6$ cells per insert). Serum-free meningeal stromal cells medium alone or conditioned medium of meningeal stromal cells (supernatant medium of meningeal stromal cells after 48 hrs of culture) or serum-free medium supplemented with CXCL10 (PeproTech) (50 ng/µl or 100 ng/µl) was added into the lower chambers. For cell-cell migration experiments, primary human meningeal stromal cells (Per, DuF, LeC, or DuEC) (0.1 X $10^6$ cells) were plated into the poly-lysine coated lower chambers 24 h prior the migration assay to achieve 85% confluency. T-ALL cells were allowed to migrate for 6 h and the levels of migrated cells were determined by trypan blue staining. In cell-cell migration experiments, migrated T-ALL cells were labelled with anti-human-CD45 antibody, followed by flow cytometry-based cell enumeration.

For inhibitor studies, T-ALL cells were pre-treated with a CXCR3 antagonist (1.5 μg/ml, for 30 minutes), TNF-α blocking antibody (anti-hTNF-α, 0.5 μg/ml, for 1 hrs; R&D Systems), and IL27 blocking antibody (anti-hIL-27, 0.5 μg/ml, for 1 hrs; R&D Systems) prior to loading into the transwell inserts. This was followed by a migration assay as described above. Cells were allowed to migrate for 6 h in presence or absence of serum-free medium supplemented with CXCL10 (100 ng/μl) was added into the lower chambers. The number of migrated cells was enumerated by trypan blue staining. For studies with stromal cells, meningeal stromal cells were pretreated with CXCL10 blocking antibody (anti-hCXCL10, 1.5 μg for 30 min), followed by a migration assay as described above. For studies involving conditioned medium, meningeal stromal cell-derived conditioned medium with or without CXCL10 blocking antibody (anti-hCXCL10, 1.5 μg), was added to the lower chamber of the transwell plate. This was followed by a migration assay for 6 h as described above.

**Transendothelial cell migration assay.** HUVEC cells or DuEC cells (1.5 x $10^5$) were seeded into the transwell inserts for 24 h (5 μm, QCM Chemotaxis Cell Migration Assay kit, Millipore Sigma). The HUVEC or DuEC cells were washed in serum free medium. T-ALL cells were suspended in serum-free RPMI-1640 (0.3 x $10^6$ cells per insert) and were loaded onto either an HUVEC or DuMEC cells monolayer. The migration assay was conducted for 6 hrs in the presence or absence of CXCL10 (100 ng/μl) in the lower chambers. The levels of migrated cells were determined by trypan blue staining.

**Adhesion assay.** The day before the assay, primary human meningeal stromal cells (Per, DuF, LeC, and DuEC) were seeded in 12-well plates at a density of 0.25 × $10^6$ cells per well in complete medium to establish semi-confluent monolayers. On the following day, 1 x $10^6$ T-ALL cells were added to each well in 1 ml of stromal medium without supplements, and co-cultures were incubated for 6 h at 37 °C with 5% $CO_2$. After incubation, non-adherent cells were removed by gentle aspiration of the medium. Adherent cells (stromal and leukemic) were detached by

treatment with trypsin (5 min, 37 °C), followed by neutralization with complete stromal medium. Collected cells were washed with FACS buffer, stained with anti-human CD45 antibody for 30 min on ice in the dark, and analyzed on a FACSAria flow cytometer to quantify the number of adherent T-ALL cells.

**Tertiary and quaternary co-cultures.** For the quaternary co-cultures, pericytes (Per, $1.5 \times 10^5$) were seeded into transwell inserts 48 h prior to the experiment. Twenty-four hours later, dural endothelial cells (DuEC, $1.5 \times 10^5$) were layered on top of the pericytes within the same inserts. For the tertiary cell co-cultures, endothelial cells (DuEC) ($1.5 \times 10^5$) were seeded into transwell inserts 24 h prior to the experiment. In parallel, primary meningeal fibroblasts or leptomeningeal cells (DuF or LeC) ($0.25 \times 10^6$) were plated in poly-lysine–coated lower chambers. T-ALL cells ($1.5 \times 10^5$) were then added to the inserts and allowed to migrate for 6 h. Following migration, the inserts were removed, and all cells from the lower chambers were collected, stained with anti-human CD45 antibody, and enumerated by flow cytometry. For the subsequent cell-to-cell adhesion assay, non-adherent cells were aspirated from the lower chamber, and adherent cells were detached by trypsinization, stained with anti-human CD45 antibody, and quantified by flow cytometry.

**Coimmunoprecipitations.** T-ALL cells ($1.5 \text{ X } 10^7$) were washed with ice-cold PBS and lysed in M-PER® Mammalian Protein Extraction Reagent supplemented with HaltTM Protease and Phosphatase Inhibitor Cocktail. Cell lysates were centrifuged at 14,000 x g (4 °C) for 15 min. Supernatant from the cell lysates was collected and incubated overnight with gentle rocking (4°C) with anti-CXCR3 or anti-USP7 or normal rabbit IgG, followed by incubation for 2 hrs at 4°C with 50 µl TrueBlot anti-Rabbit Ig IP Beads (Rockland, Gilbertsville, PA, USA, Cat. No. 88-1688-31). Bead slurry-captured immune complexes were sequentially washed with ice-cold lysis buffer by centrifugation at 2,500 x g for 30 seconds at 4°C. Immunoprecipitants were eluted by heating at 95°C for 10 min in 50 µl of 1X Sample Buffer (Bio-Rad, Hercules, CA) containing 2-

mercaptoethanol (Sigma-Aldrich). Western blot analysis was performed according to standard protocols and primary antibodies are listed in the Supplementary Table S5.

**Immunohistochemistry and imaging.** Meningeal whole-mounts were washed 3 times in PBS for 5 min, followed by incubation in PBS 0.3% Triton-X-100 containing 2% of goat serum for 1 hour at RT in 12-well plates with constant agitation. Sections were then incubated with primary antibodies: anti-CXCL10 (Bioss, bs-1502R, 1:200), anti-CXCL9 (Proteintech, 22355-1-AP, 1:200), anti-VLA-4 (Invitrogen, 14-0499-82, 5 μg/ml), and anti-VCAM-1 (R&D Systems, AF643-SP, 10 ug/ml) in PBS 0.3% Triton-X-100 containing 2% of goat serum overnight at 4°C. Meningeal whole-mounts or tissue sections were then washed 3 times for 10 min at RT in PBS followed by incubation with PE-goat, -rabbit or -mouse IgG antibodies (1:500) in PBS 0.3% Triton-X-100 containing 2% of goat serum for 1 at RT with gentle agitation. Sections were washed 2 times in PBS for 10 min, mounted on Superfrost Plus slides (Fisher Scientific), and coversliped with Fluoromount-G (SouthernBiotech). Preparations were stored at 4°C for no more than 1 week until images were acquired. Images (20x and 40x) were acquired with a Zeiss LSM 800 AiryScan confocal system using the ZEN 2.5 software (Carl Zeiss). In addition, the whole-mounts were generated at 10x magnification using GFP channel. Magnification images (10X) were stitched to whole-slice montages (Figure 1b) using the Zeiss LSM 800 AiryScan inverted microscope. The applied antibodies are listed for each experiment in the Supplementary Table S6.

**Chromatin Immunoprecipitation (ChIP).** Chromatin immunoprecipitation was performed on KOPTK1 cells as described in[2]. DNA was immunoprecipitated with anti-NOTCH1 (D1E11) and non-specific IgG antibodies (SC-2027 X) (Santa Cruz Biotechnology, TX, USA), and amplified by qRT-PCR using SYBR Green PCR Master Mix on a StepOnePlus™ Real-Time PCR System under standard conditions. Results were quantified by SYBR Green Real-Time PCR analysis. The fold enrichment of immunoprecipitated samples was normalized on INPUT and expressed

relative to the mock-treated control (IgG). PCR products were visualized after separation on 3% agarose gel stained with GelGreen Nucleic Acid Staining (Biotium, Fremont, CA, USA).

**Luciferase assay.** KOPTK1 cells were pretreated with vehicle or 0.1 μM of NOTCH1 pathway inhibitor (DBZ) for 24 hrs. The pretreated KOPTK1 cells were co-transfected with pRL and pGL3/CXCR3 promoter constructs and luciferase activity was determined after 72 h using the DualLuciferase Reporter Assay (Promega) and measured with the Synery Neo2 Plate Reader (BioTek).

### Uncategorized References


1. Wendorff, A.A. and A.A. Ferrando, *Modeling NOTCH1 driven T-cell Acute Lymphoblastic Leukemia in Mice.* Bio Protoc, 2020. **10**(10): p. e3620.
2. Sharma, N.D., et al., *Epigenetic silencing of SOCS5 potentiates JAK-STAT signaling and progression of T-cell acute lymphoblastic leukemia.* Cancer Sci, 2019. **110**(6): p. 1931-1946.

## Supplementary Table S1

| Sample | Source | Karyotype | Lesion | ETP status | CNS status | References |
|---|---|---|---|---|---|---|
| PARXPK | COG | 45,X,add(X)(q28),-9,der(12)t(9;12)(q12;p11.2)[9]/44,idem,-add(X),-5,der(9)add(9)(p21)add(9)(q21),+mar[3]/46,XX[9] | | ND | CNS1 | |
| PASFKA | COG | 46,XY[20] | TAL1_ins | ND | CNS1 | *62* |
| PASFLK | COG | ND | wt | ETP | CNS1 | *62* |
| PASGJG | COG | 46,Y,t(X;10)(p10;p10)[18]/46,XY[2] | CASK-DDX3X-MLLT10 | ND | CNS1 | *63* |
| PASJIY | COG | 46,XY,der(11)t(11;14)(p13;q11.2)t(11;15)(q21;q22), der(14)t(11;14)(p13;q11.2),der(15)t(11;15)(q21;q22)[4]/46,XY[16] | | ND | CNS1 | |
| PASKRN | COG | 46mXY,del(9)(p12),t(11;14)(p13;q11.2)[2]/46,XY[1] | wt | Not ETP | CNS1 | *62* |
| PASKXP | COG | 46,XX,t(6;11)(q25;q23),add(12)(p11.2)[6]/46,XX[14] | KMT2A-MLLT4 | ETP | CNS2 | *63* |
| PASNXS | COG | 46,XX,del(2)(q33),del(12)(p12)[20] | ND | Near ETP | CNS1 | *63* |
| PASSPP | COG | 46,XY,t(11;19)(q23;p13.3)[20]/46,XY[3] | KMT2A-MLLT1 | Not ETP | CNS2 | *62* |
| PASJLN | COG | 46,XY,add(1)(p36.3[12]/46,XY[8] | wt | ND | CNS1 | *62* |
| PASTLI | COG | 46,XY,add(9)(p13)[3]/92,idemx2[2]/46,XY,i(9)(q10)[10]/46,XY[4] | | Not ETP | CNS1 | |
| PASYHW | COG | 46,XY,t(8;14)(q24;q11)[7]/46,XY,del(6)(q13q23)[6]/46,XY[8] | | Not ETP | CNS2 | |
| PASZMC | COG | ND | KMT2A-MLLT4 | ETP | CNS1 | *63* |
| PATNIE | COG | 46,XY[20] | | Near ETP | CNS1 | |
| PATBTX | COG | 46,XY[20] | TAL1_ins | | CNS1 | *62* |
| PATCGT | COG | 45,XYY?c,dic(7;12)(p11.2;p11.2),del(9)(p13p24),t(10;11)(p12;q14),-21[3]/47,XYY?c[17] | | Not ETP | CNS1 | |
| PATRAP | COG | 46,X,add(X)(q26),del(5)(q31),t(10;11)(p12;q21)[13]/46,XX[7] | PICALM-MLLT10 | ETP | CNS2 | *63* |
| PATTTE | COG | 48,XY,add(9)(p22),add(12)(p11.2),+16,+17[3]/52,XY,+6,+9,+16,+17,+18,+21[3]/46,XY[16] | | Near ETP | CNS1 | |
| PATZZM | COG | 46,XY,t(5;14)(q35;q32)[3]/47,idem,+mar[3]/46,XY[19] | | Not ETP | CNS2 | |
| PAUATI | COG | 51,XY,+Y,+8,+10,+13,del(13)(q12q14),+19[14]/46,XY[6] | | Near ETP | CNS1 | |
| PAUHRP | COG | ND | PICALM-MLLT10 | Not ETP | CNS1 | *63* |
| Pt#1 | UNM | 46,XY,t(?;11)(?;q23). Ish (MLL x 2),(5'MLL sep 3'MLL x 1) | ND | ND | ND | |
| Pt#2 | UNM | 46,XY[4]/47,XY,+mar[1]/43,Y,-X,t(7;10)(q34;q24),-8,-19,-21,-22,+2mar[1] | ND | Near ETP | CNS2 | |
| Pt#3 | UNM | 46,XY[20] | ND | Not ETP | CNS1 | |
| Pt#4 | UNM | ND | ND | ND | ND | |
| Pt#5 | UNM | ND | ND | ND | ND | |
| Pt#6 | UNM | ND | ND | ND | ND | |
| Pt#7 | UNM | ND | ND | ND | ND | |
| ETP8 | UNM | ND | ND | ETP | ND | |

Clinical and molecular features of T-ALL samples. References indicate previously characterized and reported samples. ND, not determined; wt, wild type.

**Supplementary Table S2.**

| Sample | Specimen | Condition | Source | CNS status |
|---|---|---|---|---|
| S1 | CSF | T-ALL | UAB | CNS2 |
| S2 | CSF | T-ALL | UAB | CNS3 |
| S3 | CSF | T-ALL | UAB | CNS1 |
| S4 | CSF | T-ALL | UAB | CNS3 |
| S5 | CSF | T-ALL | UAB | CNS3 |
| S6 | CSF | T-ALL | UAB | CNS1 |
| S7 | CSF | T-ALL | UAB | CNS1 |
| S8 | CSF | normal | BioIVT | N/A |
| S9 | CSF | normal | BioIVT | N/A |
| S10 | CSF | normal | BioIVT | N/A |
| S11 | CSF | normal | BioIVT | N/A |

CSF samples from T-ALL patients and normal controls. The Children's Oncology Group (COG) uses the following definitions to classify central nervous system (CNS) status in patients with leukemia: CNS1, no blasts in the CSF and a white blood cell (WBC) count in the CSF of less than 5/μL; CNS2, blasts in the CSF and a WBC count in the CSF of less than 5/μL; CNS3, blasts in the CSF and a WBC count in the CSF of 5/μL or greater, or clinical signs of CNS leukemia. N/A, not applicable.

**Supplementary Table S2**

| Plasmids | | | |
|---|---|---|---|
| **Name** | **Cat#** | **sequence/specfication** | **source** |
| **Lentiviral plasmids** | | | |
| CXCR3 KO1 | VSGH11936-247535319 | AGTTCAGGCTGAAGTCCTGT | Dharmacon |
| CXCR3 KO2 | VSGH11936-247493203 | CCACCTGTGGGAAGTTGTAT | Dharmacon |
| sgCtrl | VSGC11954 | GATACGTCGGTACCGGACCG | Dharmacon |
| sgCtrl | VB010000-9354ztt | GTGTAGTTCGACCATTCGTG | VectorBuilder Inc |
| CXCL10 KO1 | VB900132-5065aku | CGTGGACAAAATTGGCTTGC | VectorBuilder Inc |
| CXCL10 KO2 | VB900132-5066hwq | ACTCACATGATCTCAACACG | VectorBuilder Inc |
| TNF KO1 | VB900136-2270cuw | TGGTGGCGCCTGCCACGATC | VectorBuilder Inc |
| TNF KO2 | VB900136-2271qmj | TGGCCCGGCGGTTCAGCCAC | VectorBuilder Inc |
| IL27 KO1 | VB220915-1001vba | ACTCACAGAGAGGCGGCGCC | VectorBuilder Inc |
| Il27 KO2 | VB220915-1004fgt | GACTTACAAAGCGGTGGGCC | VectorBuilder Inc |
| CXCR3 - TV1 | CwW307854 | NM_001504 | Origene |
| pLenti-mRFP-P2A-Puro | PS100094 | Empty vector | Origene |
| pLenti-C-mGFP-P2A-Puro | PS100093 | Empty vector | Origene |
| CXCR3 - TV2 | Cw307855 | NM-001142797 | Origene |
| | Source | | |
| USP7 WT | N/A | pQflag-USP7WT puro R | Panagiotis Ntziachristos |
| USP7 CS | N/A | pQflag-USP7 CS puro R | Panagiotis Ntziachristos |
| shUSP7-1 | VB900068-4184xrs | GTGTCCTATATCCAGTGTAAA | VectorBuilder Inc |
| shUSP7-2 | VB900068-4191rjg | CGTGGTGTCAAGGTGTACTAA | VectorBuilder Inc |
| shNC | VB010000-0009mxc | CCTAAGGTTAAGTCGCCCTCG | VectorBuilder Inc |
| shCXCR3 -1 | VB900200-3415dhn | CCTTCTCATTTGGAAACTAAA | VectorBuilder Inc |
| shCXCR3 -2 | VB900202-6649jrm | GCCCTTCTCATTTGGAAACTA | VectorBuilder Inc |
| Active β-catenin plasmid | p114281 | pCWXPGR-pTF-betaCatenin- | Addgene |

| | | | |
|---|---|---|---|
| Red Luciferase | Q#11641 | pLenti-EF1a (Red-Luciferase)-Rsv (RFP-Blast) | GenTarget Inc |
| **Retroviral plasmids** | | | |
| ΔE-NOTCH1-pMSCV | | Phoenix cell line packaging | Adolfo Ferrando |
| pMSCV empty vector control | | Phoenix cell line packaging | Adolfo Ferrando |

**Supplementary Table S3**

| **TaqMan assay probes** | |
|---|---|
| human CXCR3 | Hs01847760_s1 |
| human CXCL10 | Hs00171042_m1 |
| human CXCL9 | Hs00171065_m1 |
| human CXCL11 | Hs00171138_m1 |
| human VCAM1 | Hs01003372_m1 |
| human USP7 | Hs00931763_m1 |
| Human TNF alpha | Hs00174128_m1 |
| Human IL27 | Hs00377366_m1 |
| Human IFNG | Hs00989291_m1 |
| human GAPDH | Hs99999905_m1 |
| mouse Cxcr3 | Mm99999054_s1 |
| mouse Cxcl10 | Mm00445235_m1 |
| Mouse Cxcl9 | Mm00434946_m1 |
| mouse Tnf alpha | Mm00443258_m1 |
| mouse IL27 | Mm00461162_m1 |
| mouse Ifng | Mm01168134_m1 |
| Mouse Gapdh | Mm99999915_g1 |

**Supplementary Table S4**

| **Antibodies used for flow cytometry** | | | | |
|---|---|---|---|---|
| **Mouse stromal cell panel** | | | | |
| **Dye/target/ Intracellular signaling** | **Fluorophore** | **Catalog#** | **Channel used with LSR Fortessa** | **Company** |
| CXCL10 | | BS-1502R | | ThermoFisher |
| Desmin | BV421 | NBP2-54503AF350 | BV421 | Novus Biologicals |
| AN2 | PE | 130-123-730 | PE | Miltenyi Biotec |
| CD13 | BV711 | 740695 | BV711 | BD Biosciences |
| CD31 | PE/Dazzle 594 | 563616 | PE/Dazzle 594 | BD Biosciences |
| PDGFR-a | BUV661 | 741563 | BUV661 | BD Biosciences |
| Goat anti-Rabbit IgG (H+L) Cross-Adsorbed Secondary Antibody, DyLight™ 800 | PE-Cy7 | A10542 | PE-Cy7 | Invitrogen |
| | | | | |
| **Mouse targets** | | | | |
| CD4 | APC | 553051 | APC | BD Biosciences |
| CD8A | BV421 | 563898 | Pacific Blue | BD Biosciences |
| CXCR3 | PE | 12-1831-82 | PE | Invitrogen |
| Armenian Hamster IgG Isotype Control | PE | 12-4888-81 | PE | BD Biosciences |
| CD45 | BV421 | 563890 | Pacific Blue | BD Biosciences |
| Ly-6A/E (Sca-1) | APC | 17598182 | APC | Invitrogen |
| Ki-67 | PE | 567719 | PE | BD Biosciences |
| CD45 | PE-CF594 | 562420 | PE-CF594 | BD Biosciences |
| IFN-γ | PE | 554412 | PE | BD Biosciences |
| TNF | BV421 | 563387 | BV421 | BD Biosciences |
| IL-27 | R718 | 568410 | R718 | BD Biosciences |
| INFGR1 | BV711 | 740706 | BV711 | BD Biosciences |
| IL-27Rα | PE | 564337 | PE | BD Biosciences |
| TNFRI | APC | 113006 | APC | BD Biosciences |
| CD31 | BV421 | 562939 | BV421 | BD Biosciences |
| CD13 | RB780 | 755512 | RB780 | BD Biosciences |
| CD45.1 | BV650 | 563754 | APC | BD Biosciences |
| CD45.2 | PE-Cy7 | 560696 | PE-Cy7 | BD Biosciences |

| | | | | |
|---|---|---|---|---|
| **Human targets** | | | | |
| CXCR3 | APC | 17-1839-42 | APC | Invitrogen |
| CXCR3 | PE | 12-1839-42 | PE | Invitrogen |
| Mouse IgG1 kappa Isotype Control | PE | 12-4714-81 | PE | Invitrogen |
| Mouse IgG1 kappa Isotype Control | APC | 17-4714-82 | APC | Invitrogen |
| CD49d (VLA-4) | PE | 555503 | PE | BD Biosciences |
| PE Mouse IgG1, κ Isotype Control | PE | 555749 | PE | BD Biosciences |
| CD45 | APC | 555485 | APC | BD Biosciences |
| CCR7 | BV421 | 740052 | BV421 | BD Biosciences |
| CXCR4 | APC | 555976 | APC | BD Biosciences |
| CD3 | APC-CY7 | 557832 | APC-CY7 | BD Biosciences |
| CD8 | FITC | 555366 | FITC | BD Biosciences |
| CD4 | PE | 555347 | PE | BD Biosciences |

**Supplementary Table S5**

| **Antibodies used for Western blot** | | |
|---|---|---|
| **Name** | **Catalog#** | **Company** |
| CXCR3 | ab154033 | Abcam |
| Actin | E4D9Z | Cell Signalling Technology |
| Phospho-Akt (Ser473) | D9E | Cell Signalling Technology |
| Akt | 40D4 | Cell Signalling Technology |
| Phospho-Erk1/2 (Thr202/Tyr204) | 9101S | Cell Signalling Technology |
| Erk1/2 | L34F12 | Cell Signalling Technology |
| Phospho-SAPK/JNK (Thr183/Tyr185) | 4688T | Cell Signalling Technology |
| SAPK/JNK | 9252T | Cell Signalling Technology |
| Non-phospho β-Catenin (Ser33/37/Thr41) | D13A1 | Cell Signalling Technology |
| Cortactin | 3503S | Cell Signalling Technology |
| FAK | 13009S | Cell Signalling Technology |
| Paxillin | D9G12 | Cell Signalling Technology |
| Ezrin/Radixin/Moesin | 3142T | Cell Signalling Technology |
| Vinculin | 4650T | Cell Signalling Technology |
| CXCL10 | D5L5L | Cell Signalling Technology |
| USP7 | SAB4200042 | Sigma-Aldrich |
| HRP-linked Anti-rat IgG | 7077 | Cell Signalling Technology |
| Ubiquitin | P37 | Cell Signalling Technology |
| CXCL10 | D5L5L | Cell Signalling Technology |
| CXCL9 | E6Z5W | Cell Signalling Technology |
| CXCL11 | PA5-104147 | Invitrogen |
| GAPDH | D16H11 | Cell Signalling Technology |
| Na,K-ATPase α1 | 23565 | Cell Signalling Technology |
| IL-27 | AF2526 | R&D Systems |
| Phospho-β-Catenin (Ser33/37/Thr41) | 9561 | Cell Signalling Technology |
| Lamin A/C | 10298-1-AP | Proteintech |
| TNF-alpha | AF-410-NA | R&D Systems |
| HRP-linked anti-rabbit IgG | 7074 | Cell Signalling Technology |
| HRP-linked anti-mouse IgG | 7076 | Cell Signalling Technology |
| **Antibodies used for IP** | | |
| USP7 | PA5-34911 | Invitrogen |
| Normal Rabbit IgG | 2729 | Cell Signalling Technology |
| 3X DYKDDDDK Tag | E7C5T | Cell Signalling Technology |
| Notch1 | D1E11 | Cell Signalling Technology |
| **Antibodies used for CHIP** | | |
| Notch1 | D1E11 | Cell Signalling Technology |
| non-specific IgG antibodies | SC-2027 X | Santa Cruz Biotechnology |

**Supplementary Table S6**

| Antibodies used for Immunohistochemistry | | |
|---|---|---|
| **Name** | **Catalog#** | **Company** |
| CXCL10 | bs-1502R | Proteintech |
| CXCL9 | 22355-1-AP | Bioss |
| VCAM-1 | AF643-SP | R&D Systems |
| VLA-4 | 14-0499-82 | Invitrogen |
| PE F(ab')2 fragment goat anti rabbit | A10542 | Invitrogen |
| PE F(ab')2 fragment goat anti mouse | 115-116-146 | Jackson ImmunoResearch Labs |

## Supplemental Figure 1

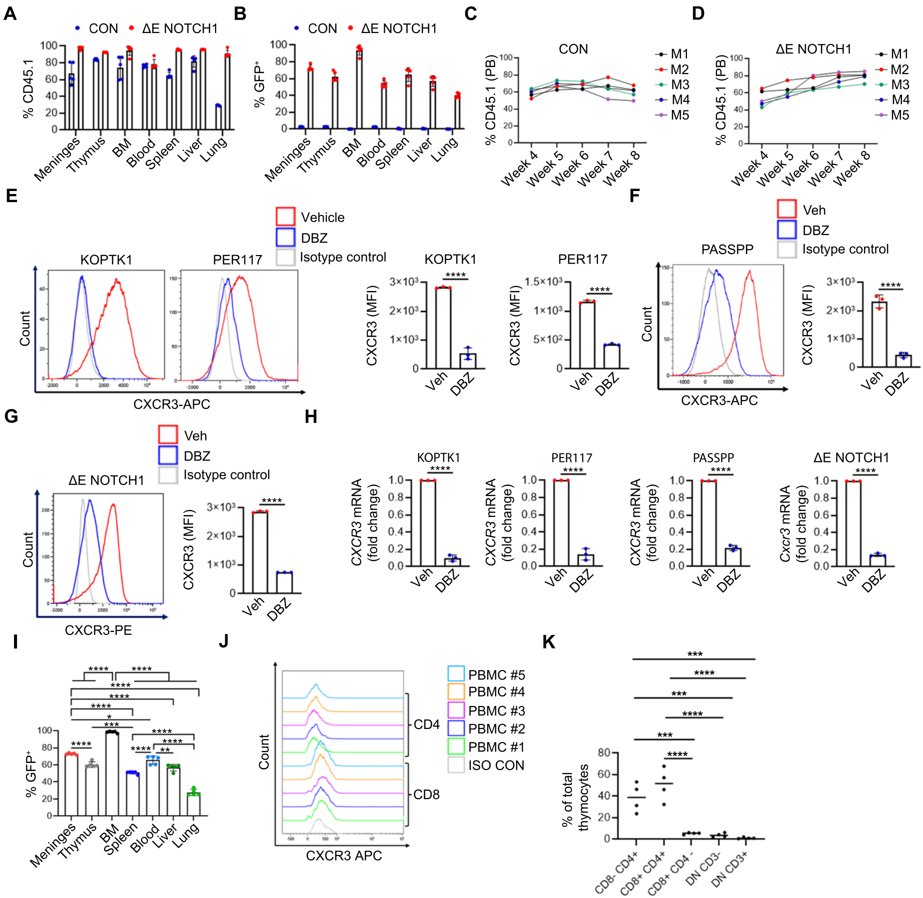

## Supplemental Figure 2 Part 1

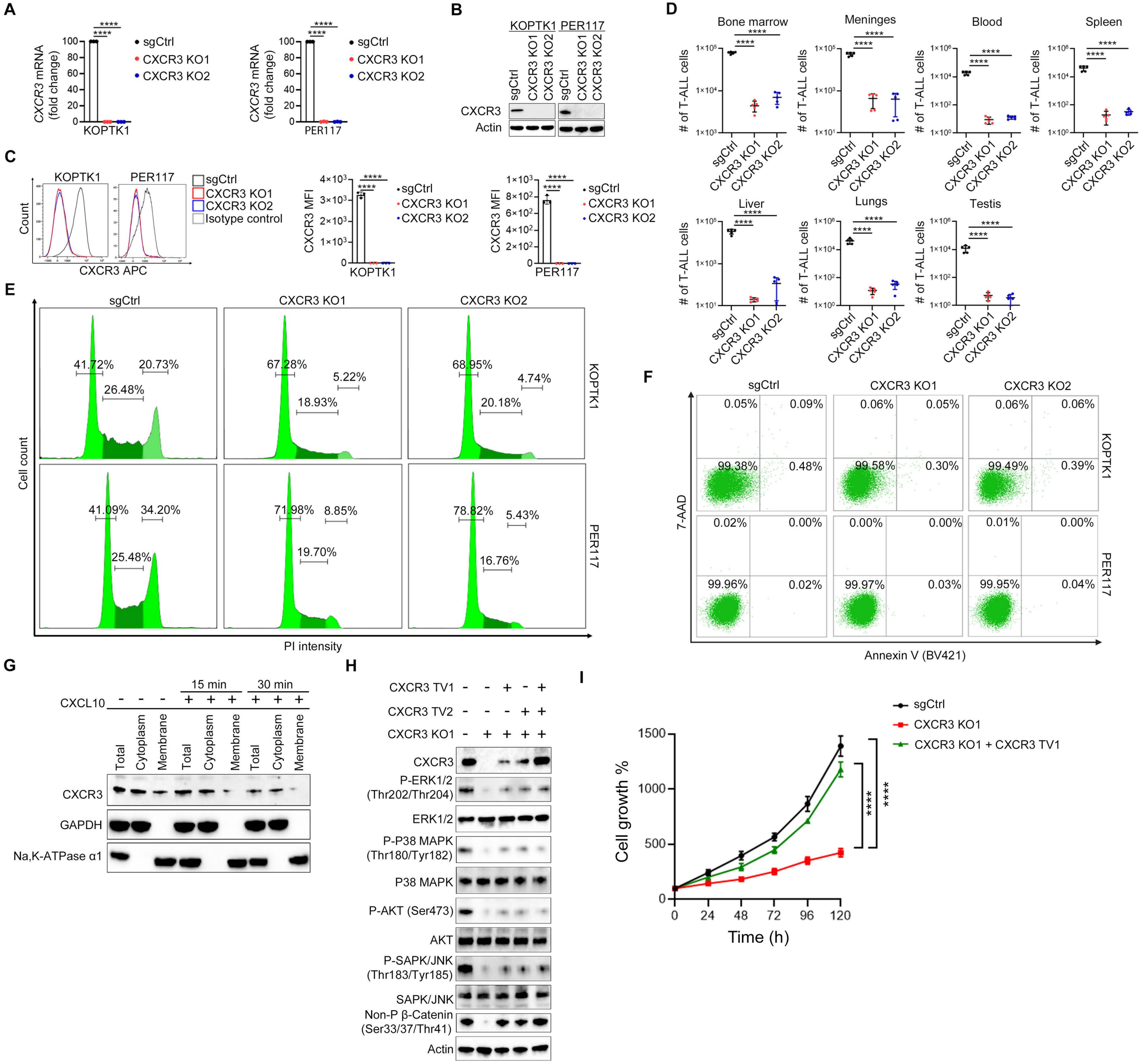

# Supplemental Figure 2 Part 2

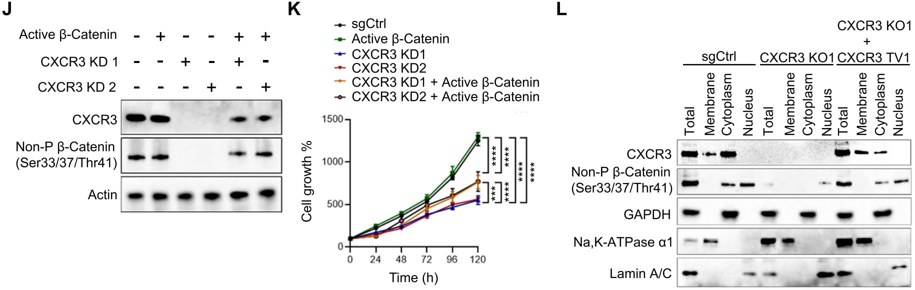

# Supplemental Figure 3 Part 1

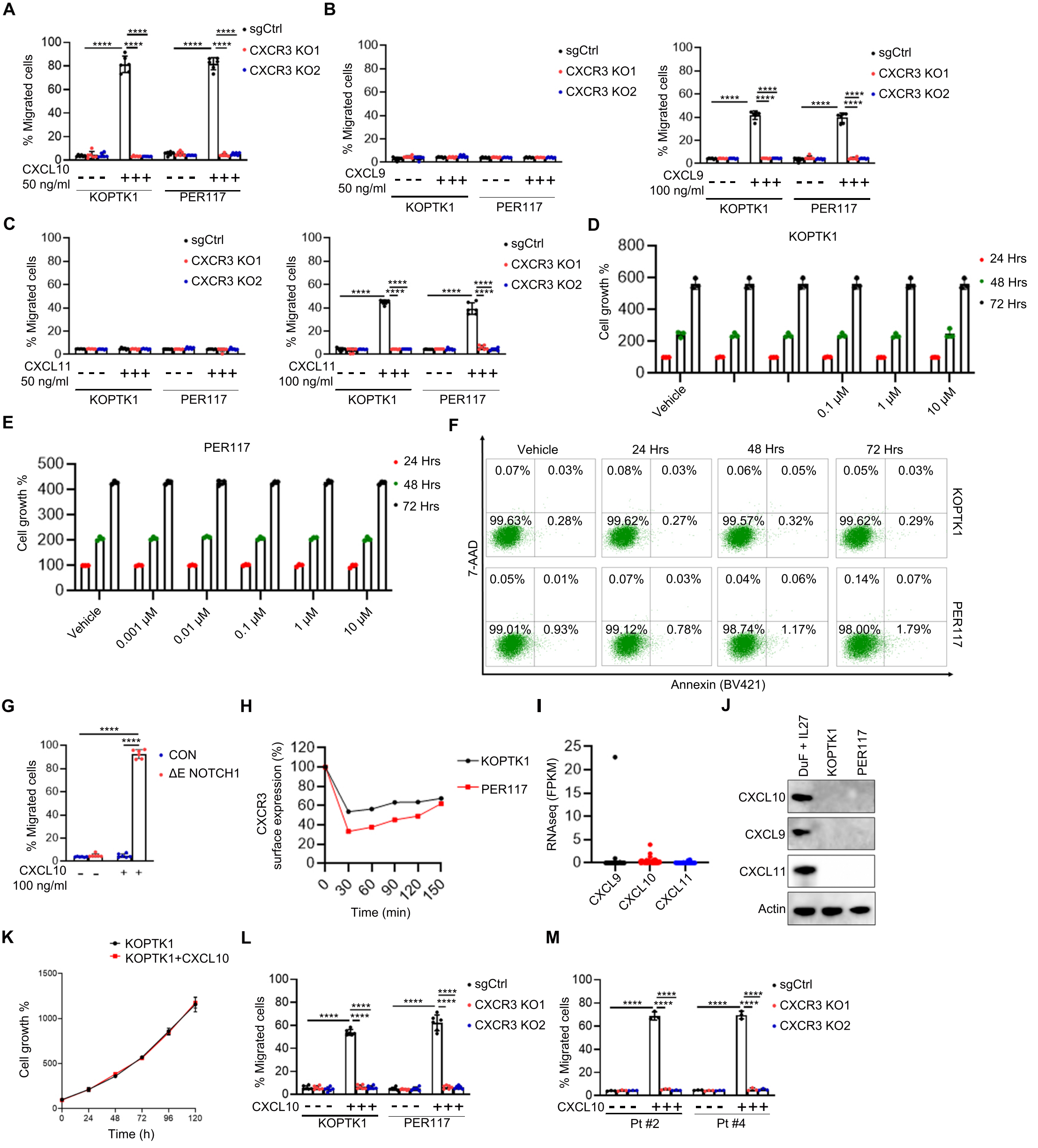

## Supplemental Figure 3 Part 2

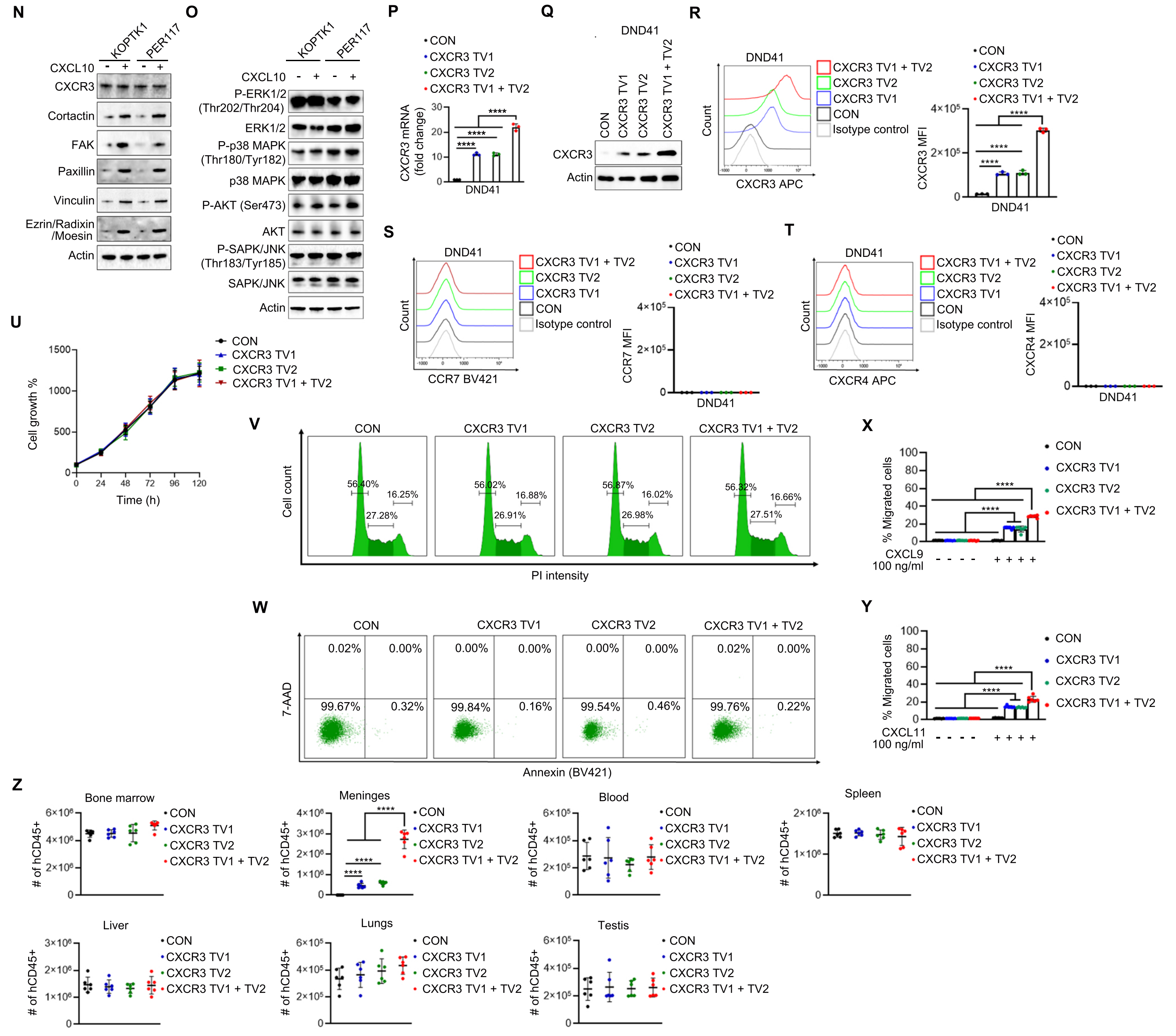

## Supplemental Figure 4

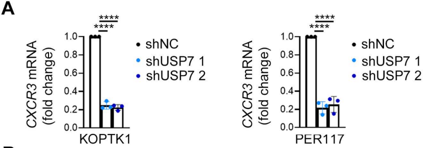


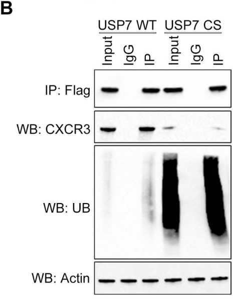

**Supplemental Figure 5 part 1**

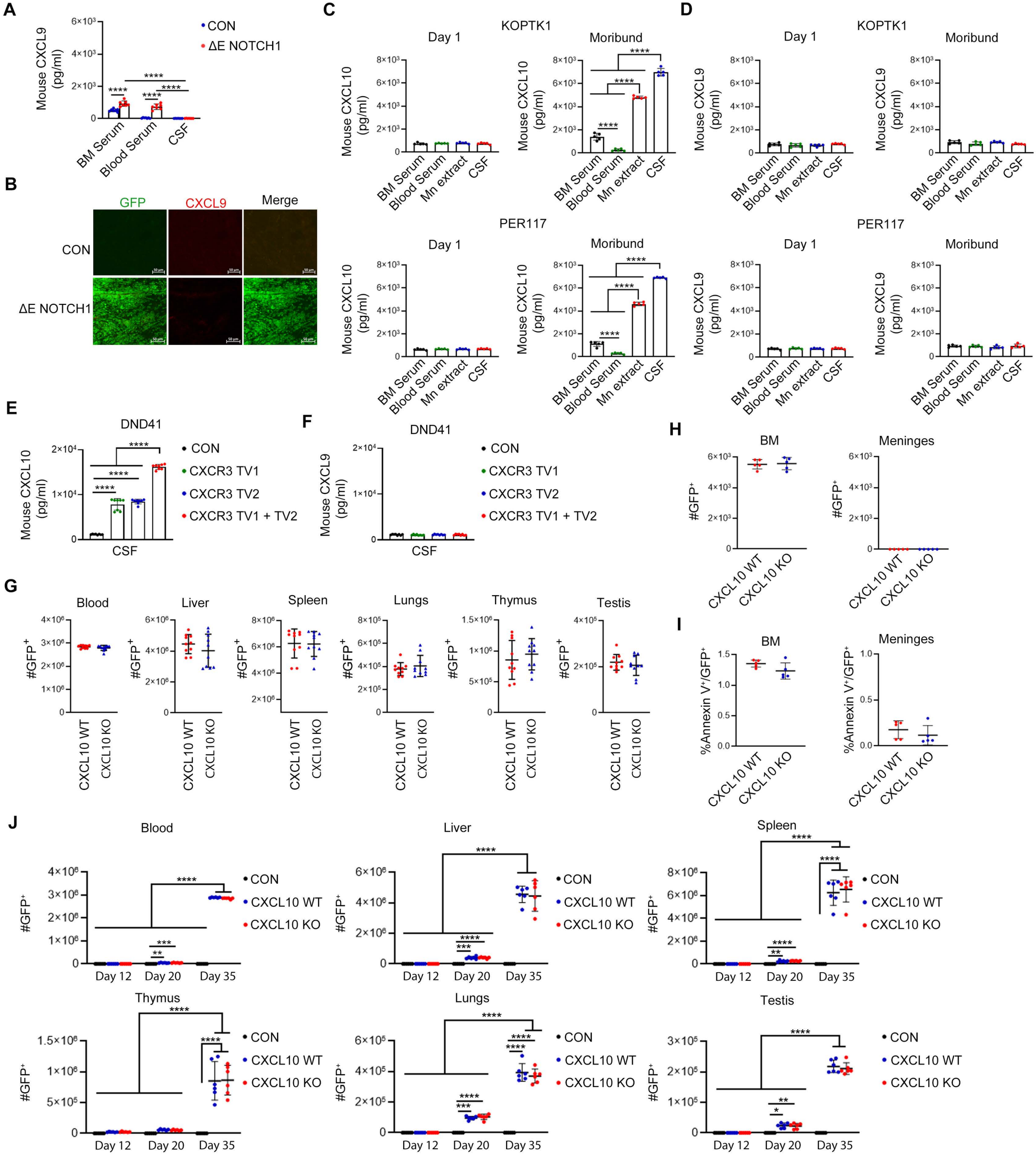

Supplemental Figure 5 part 2

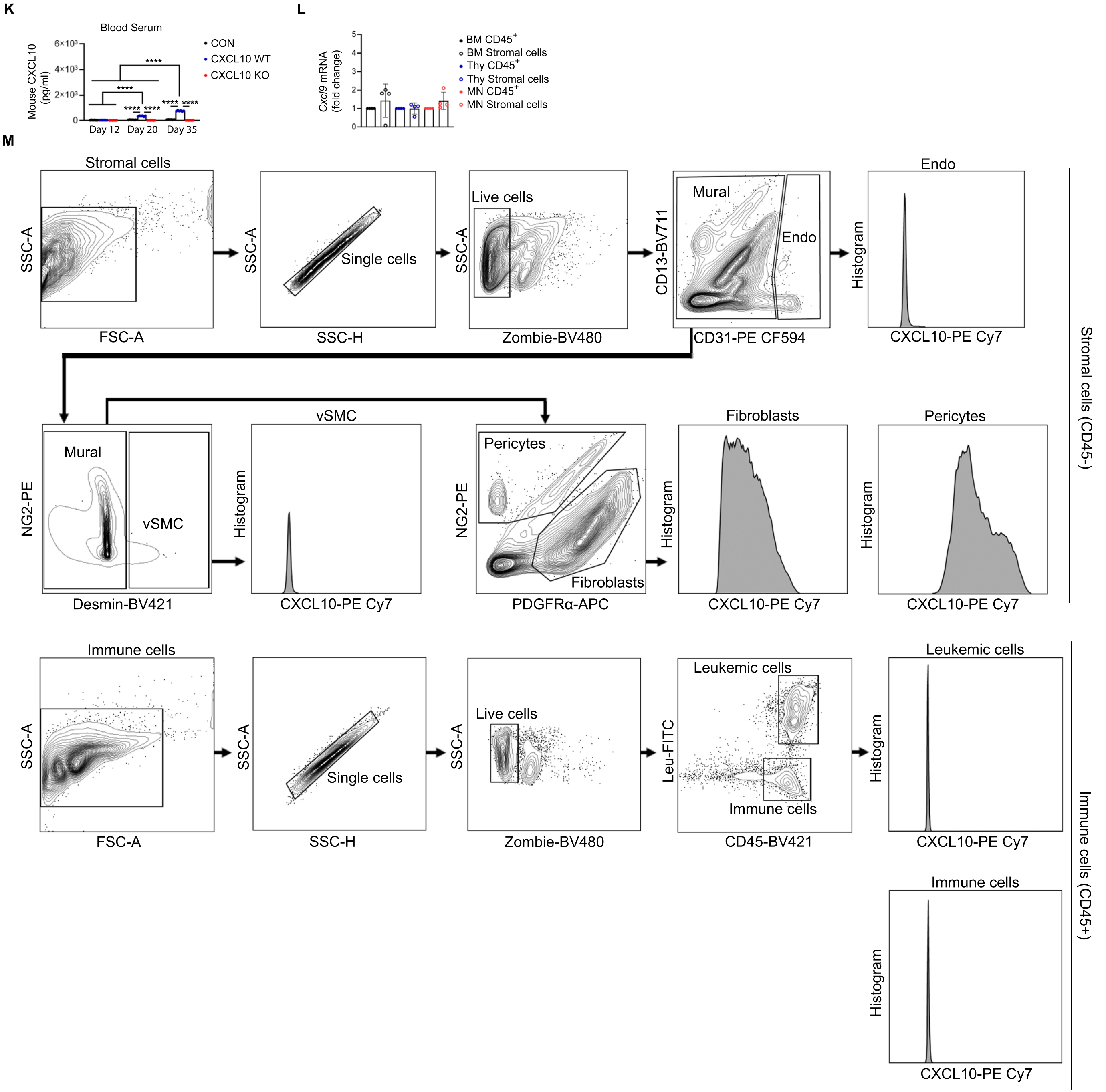

**Supplemental Figure 5 part 3**

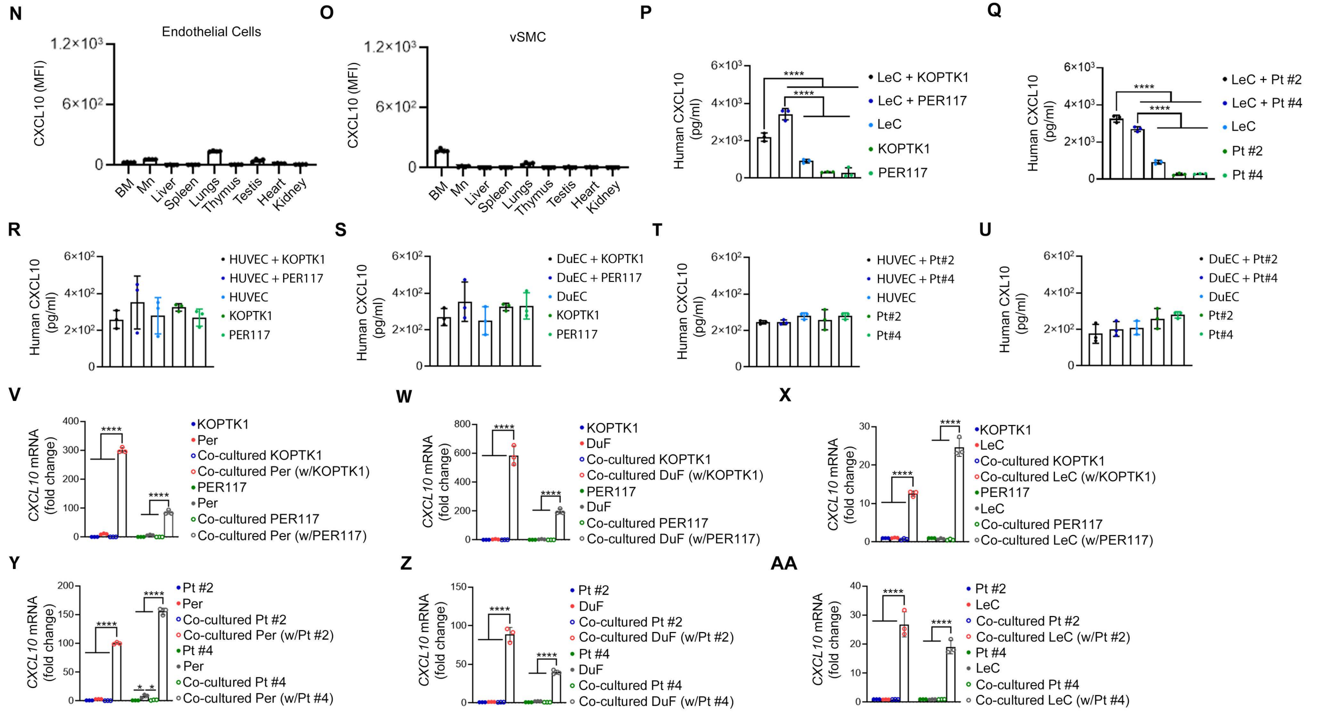

**Supplemental Figure 6**

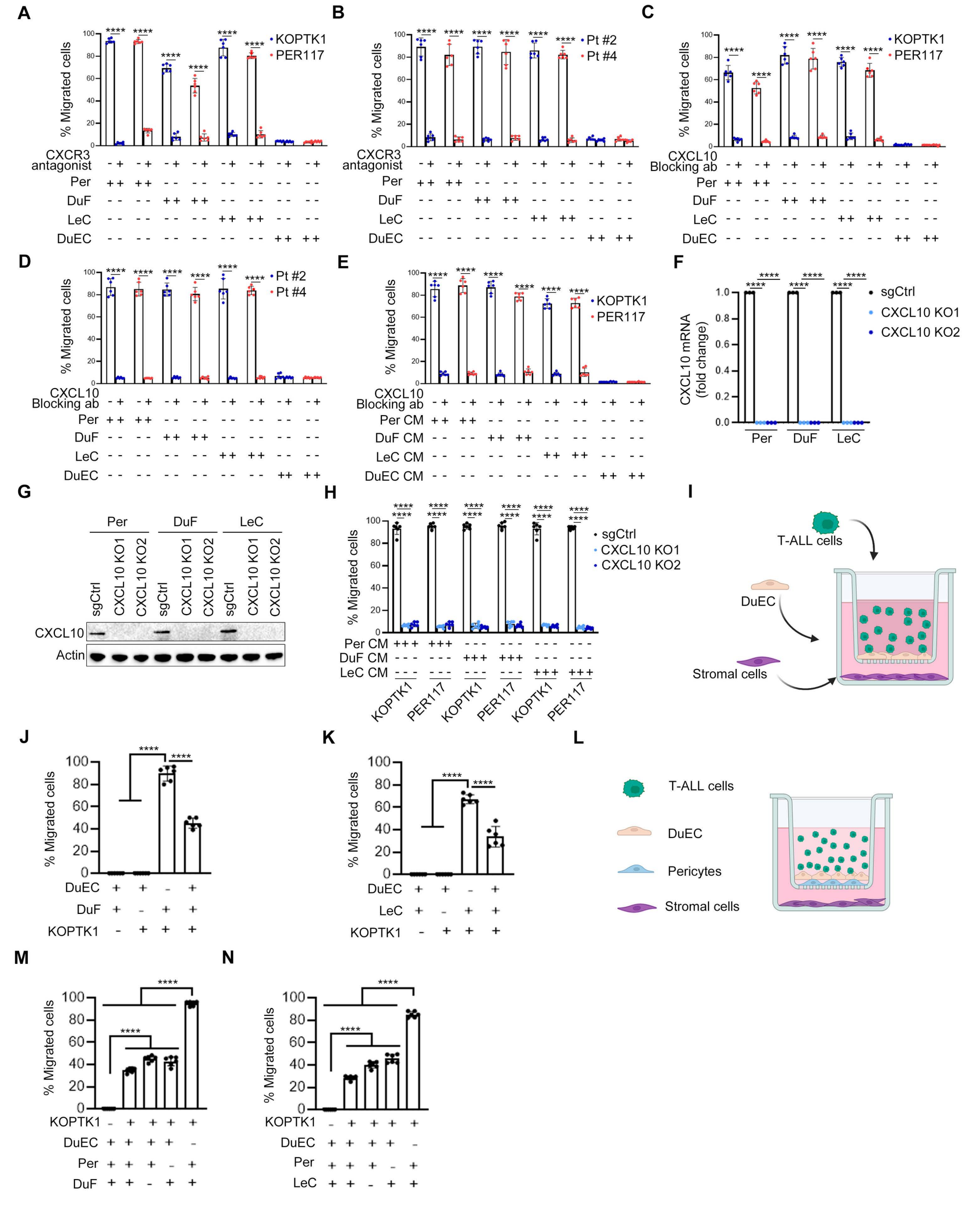

## Supplemental Figure 7 Part 1

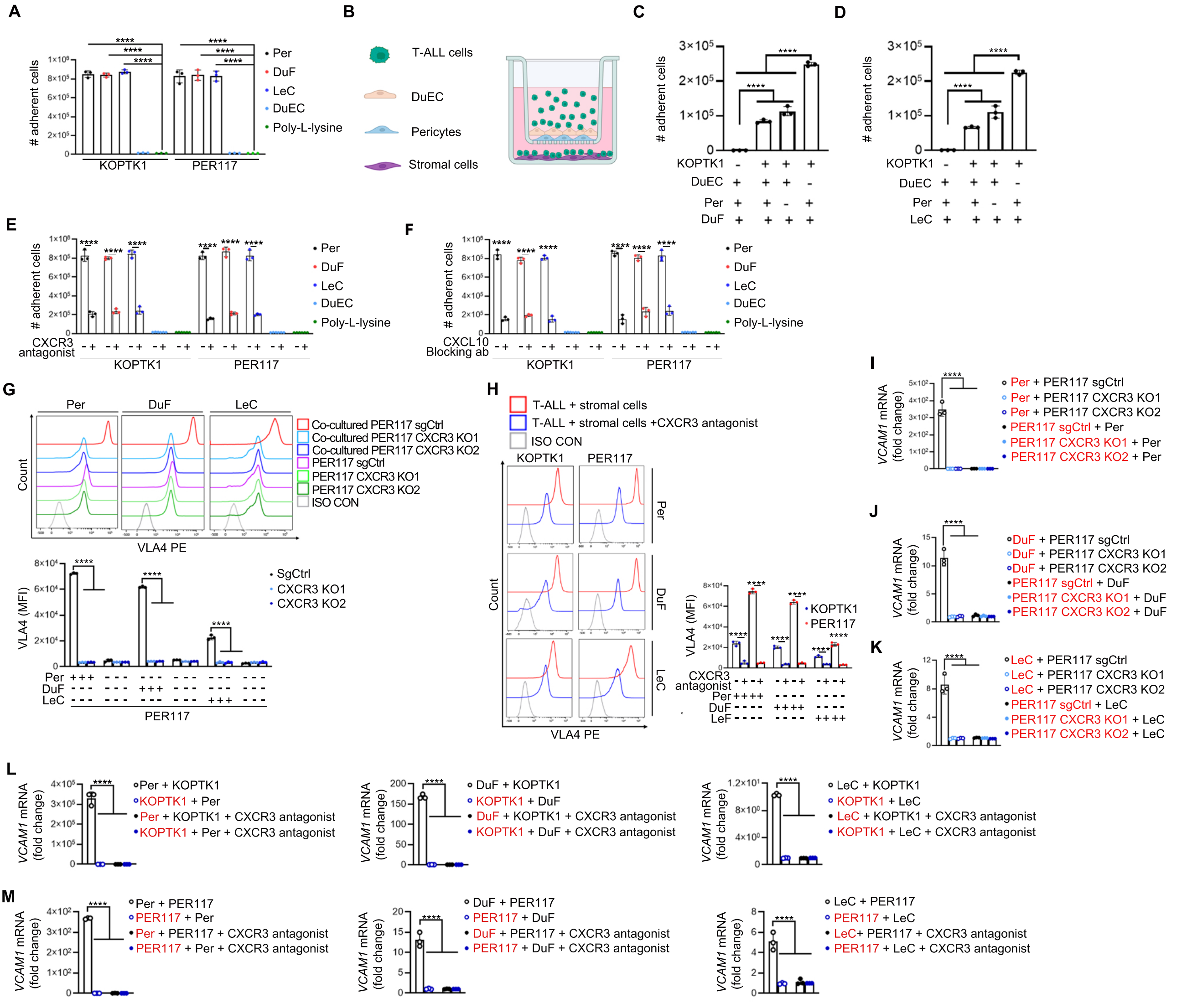

## Supplemental Figure 7 Part 2

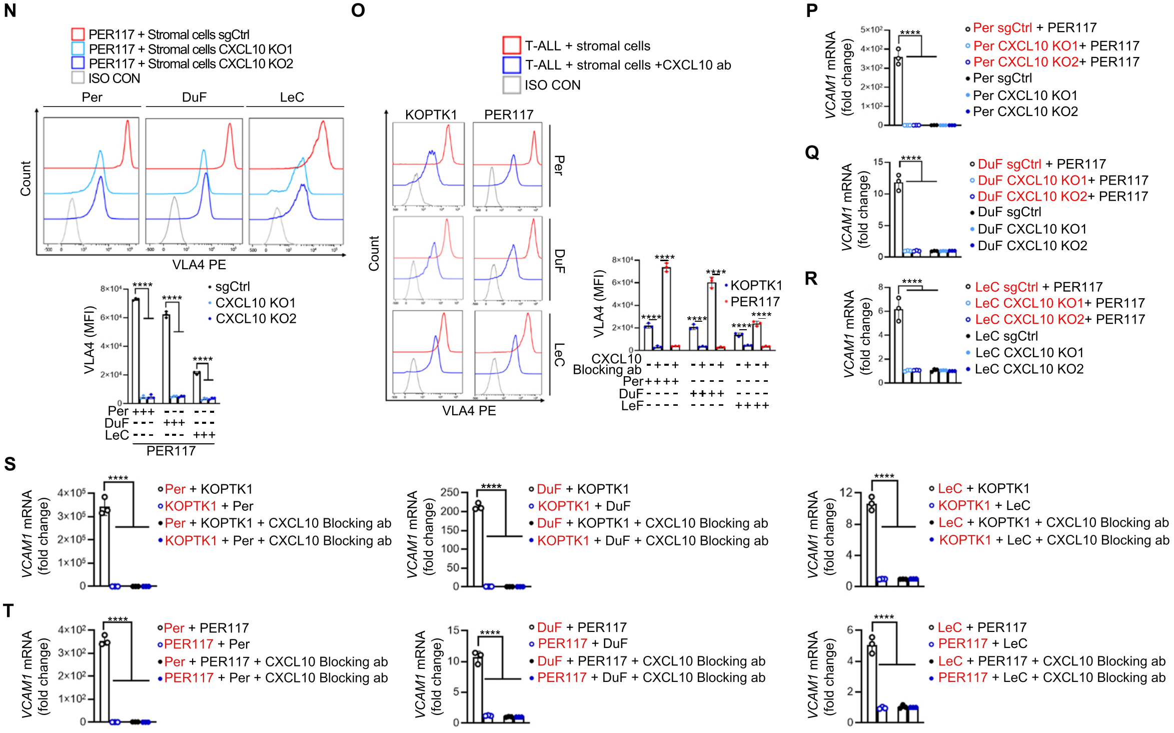

# Supplemental Figure 8 part 1

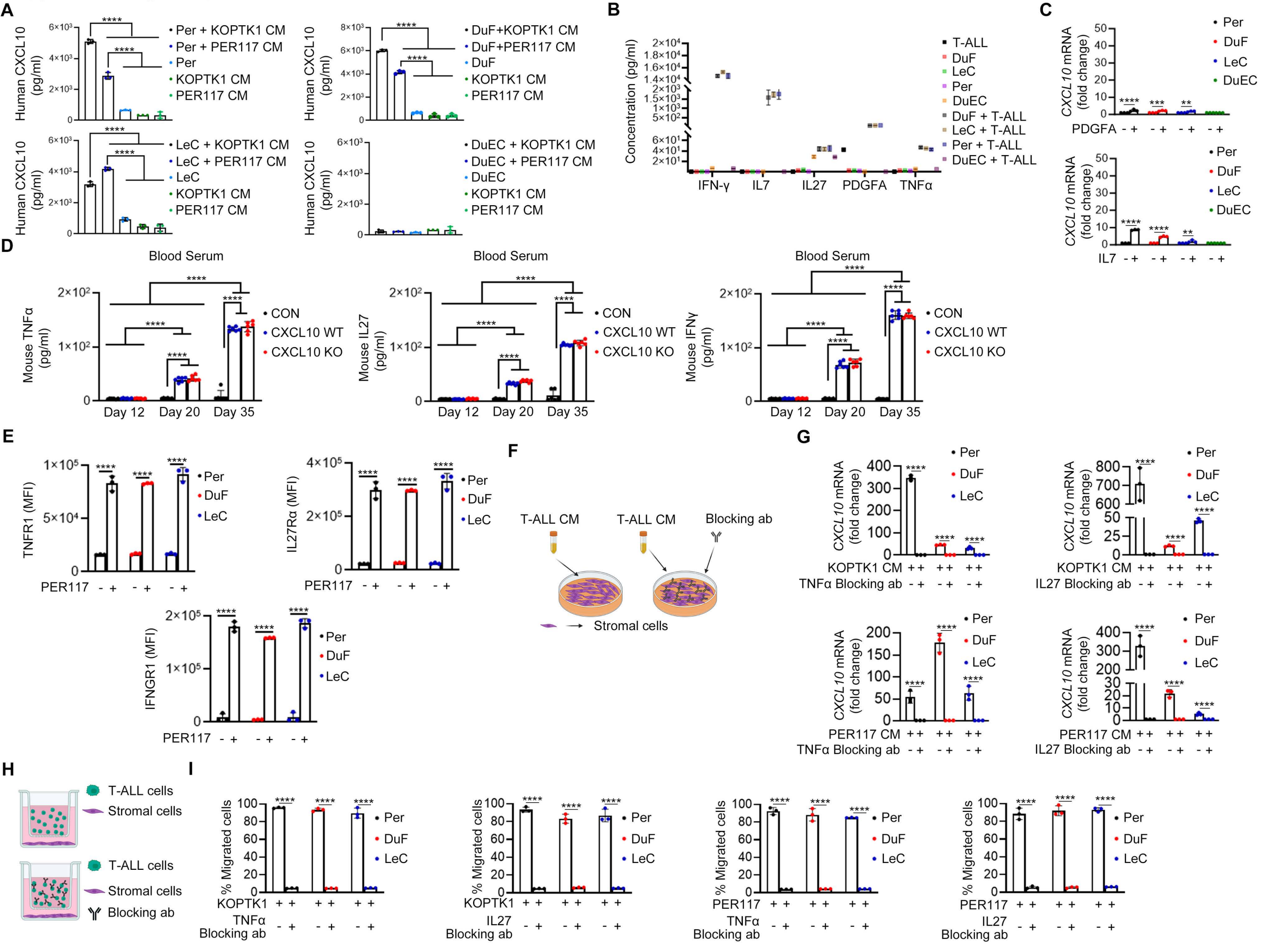

## Supplemental Figure 8 part 2

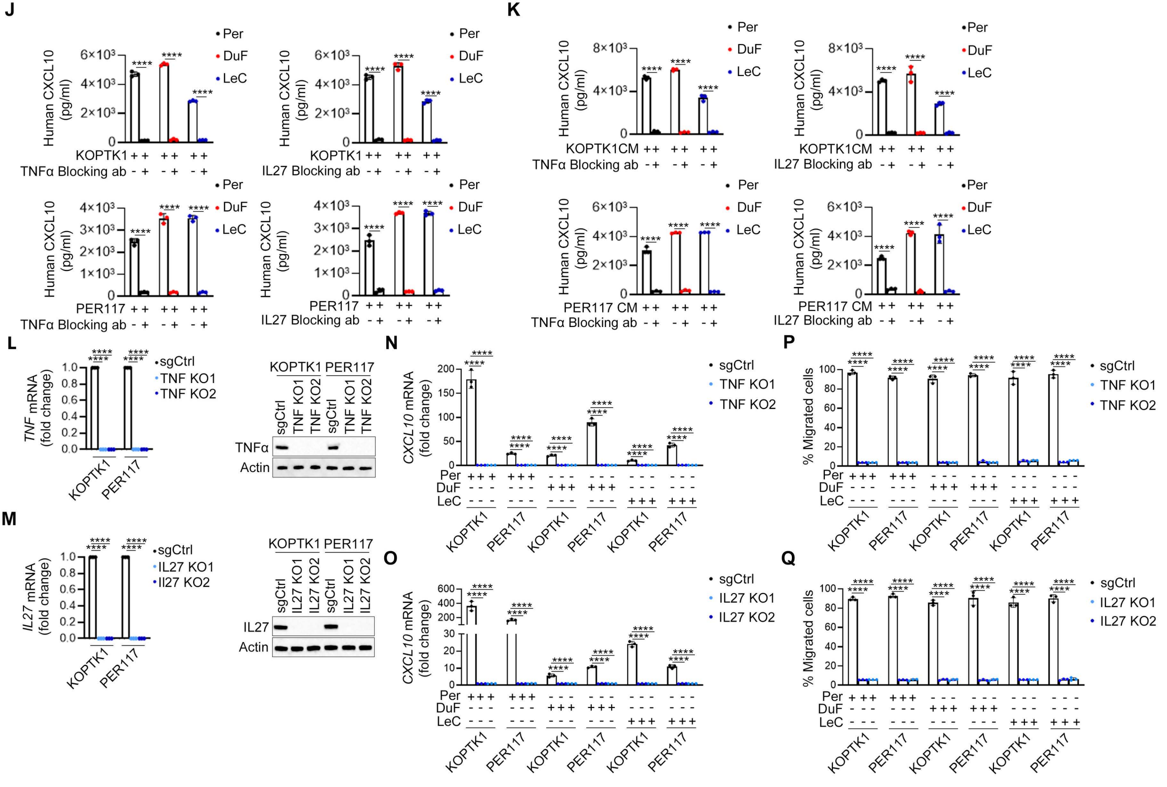

## Supplemental Figure 9

**A**

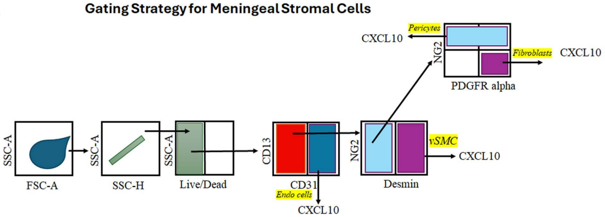


**B**

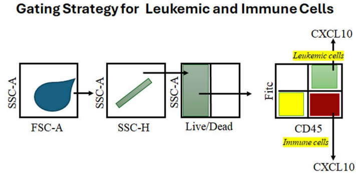

## Supplementary Figure Legends

**Supplementary Figure 1. CXCR3 is induced during T-ALL progression.** (**A**-**D**) Generation of oncogenic ΔE-NOTCH1-driven T-ALL. Bone marrow lineage-negative ($Lin^-$) progenitor cells from donor $CD45.1^+$ mice were transduced with ΔE-NOTCH1-GFP or control-GFP (CON) vectors and transplanted into irradiated $CD45.2^+$ recipients (n = 5/group). **(A)** Distribution of donor-derived CD45.1 cells in the BM, blood and peripheral organs of recipient mice. **(B)** Levels of $GFP^+$ cells (% $GFP^+$) in control (CON) and T-ALL (ΔE-NOTCH1) mice. Data were obtained from moribund T-ALL (ΔE-NOTCH1) mice and age-matched controls (CON). **(C** and **D)** Levels of $CD45.1^+$ donor cells in the peripheral blood of ΔE-NOTCH1 and CON mice at indicated time points post-transplantation. (**E**-**H**). T-ALL cells were treated with DBZ, a γ-secretase inhibitor (0.1 μM) for 24 hours. CXCR3 levels in DBZ- and vehicle-treated (**E**) KOPTK1 and PER117 cell lines, (**F**) primary T-ALL sample, (**G**) *ΔE-NOTCH1*-transformed murine hematopoietic progenitors. Left panels show representative histograms; right panels show MFI ± SD from 3 independent experiments. (**H**) *CXCR3* mRNA expression human KOPTK1, PER117 cells lines, PASSPP primary T-ALL cells, and murine (ΔE-NOTCH1) T-ALL cells following treatment with DBZ or vehicle control (0.1 μM, 24 h). (**I**) T-ALL cells (GFP+) isolated from the BM, blood and other organs of moribund ΔE-NOTCH1 mice (n = 5). Data represents the percentage of GFP+ positive cells in hematopoietic (CD45+) cell population. (**J**) CXCR3 expression (MFI) in normal human $CD8^+$ and $CD4^+$ T-cell subsets in peripheral blood (n = 5 donors). (**K**) Percentage of normal human thymic T-cell subsets in the thymus relative to the total number of cells analyzed (n = 4 donors). (**E**–**H**) Data are presented as mean ± SD from 3 separate experiments. Unpaired t-test; ****P < 0.0001.

**Supplemental Figure 2. CXCR3 knockout impairs T-ALL cell growth and disease dissemination.** (**A**-**C**) KOPTK1 and PER117 T-ALL cell lines were transduced with lentivirus expressing sgRNAs targeting *CXCR3* (CXCR3 KO1 and CXCR3 KO2) and a negative control sgRNA (sgCtrl). Loss of CXCR3 (**A**) transcript and (**B**) protein followed by CRISPR/Cas9-

mediated knockout of *CXCR3* in KOPTK1 and PER117 cells. Means ± SD of three independent experiments. Representative blots from at least 3 separate experiments. (**C**) CXCR3 cell surface levels in T-ALL cell lines (KOPTK1, PER117) carrying CRISPR/Cas9-mediated knockout of *CXCR3* (CXCR3 KO1, CXCR3 KO2, sgRNAs targeting CXCR3*;* SgCtrl, negative control). Representative histograms (left); MFI ± SD, 3 independent experiments (right). (**D**) Total number of leukemic cells isolated from the BM (femurs), meninges, blood, spleen, liver, lungs, and testis of NSG mice (n = 5/group) inoculated intrafemorally with transduced KOPTK1 cells (3 x $10^5$ cells/mouse). All mice were sacrificed at day 45 time point. (**E**) Cell cycle analysis and (**F**) Annexin V and 7-AAD staining by flow cytometry (48 hours). Data are representative of 1 of 3 independent experiments. (**G**) Cytoplasmic and membrane-associated CXCR3 fractions in KOPTK1 T-ALL cell line stimulated with CXCL10 (100 ng/ml). GAPDH and Na,K-ATPase α1 represent cytoplasmic and membrane controls, respectively. (**H**) KOPTK1 cells were co-transduced with sgRNAs targeting CXCR3 (CXCR3 KO1) and/or plasmids expressing CXCR3 variants (CXCR3 TV1, CXCR3 TV2, or CXCR3 TV1 + TV2) and relative negative controls. (**I**) Rescue of cell growth in CXCR3-depleted KOPTK1 cells upon co-transduction with a plasmid expressing CXCR3 (CXCR3 TV1). (**J**) KOPTK1 cells were co-transduced with shRNA targeting CXCR3 (CXCR3 KD 1, CXCR3 KD 2) and/or a plasmid expressing non-phosphorylated, active β-catenin (ser33/37/Thr41). (**K**) Cell growth rescue in KOPTK1 cells with shRNA mediated CXCR3 silencing (CXCR3 KD1, CXCR3 KD2) and/or expression of active β-catenin. (**L**) Cytoplasmic, nuclear and membrane fractions in KOPTK1 cells co-transduced with sgRNA targeting CXCR3 (CXCR3 KO1) and/or a plasmid expressing CXCR3 variant (CXCR3 TV1) and relative negative controls. (**I** and **K**). Mean ± SD for 1 of 3 independent experiments performed in triplicate. Repeated measure ANOVA with Tukey's multiple comparisons test. (**G**, **H**, **J** and **L**) Western blot analysis of the indicated proteins. Representative blot from 3 independent experiments. (**A**, **C** and **D**) one-way ANOVA with Tukey's multiple comparison test, ****P < 0.0001.

**Supplemental Figure 3. CXCR3 regulates T-ALL cell migration.** Migration of T-ALL cell lines upon CXCR3 knockout (6 h) through 3 µm porous membrane towards (**A**) ± CXCL10 (50 ng/ml), (**B**) ± CXCL9 (50 and 100 ng/ml), (**C**) ± CXCL11 (50 and 100 ng/ml). Data are shown as mean ± SD, 3 separate experiments performed in duplicate. (**D**–**F**) KOPTK1 and PER117 cells were treated with the CXCR3 antagonist AMG487 at concentrations ranging from 0 to 10 µM for 24, 48, and 72 hours, respectively. (**D** and **E**) Cell proliferation was measured by MTS assay. Representative of one of three independent experiments, each performed in triplicate. (**F**) Apoptosis was assessed by Annexin V and 7-AAD staining followed by flow cytometry. Data are representative of one of three independent experiments. (**G**) Migration of murine *ΔE-NOTCH1*-transformed T-ALL cells (6 h) through 3 µm porous membrane toward ± CXCL10 (100 ng/ml). Data are shown as mean ± SD, 3 separate experiments performed in duplicate. (**H**) Kinetics of CXCL10-induced (100 ng/ml) internalization of CXCR3 in T-ALL cell lines measured by flow cytometry. Data are representative of 1 of 3 independent experiments. (**I**) Expression of CXCL9, CXCL10 and CXCL11 in a published dataset of 264 primary T-ALL patient samples (Liu *et al.,* 2017, Nat Genet). (**J**) CXCL9, CXCL10 and CXCL11 protein levels in KOPTK1 and PER117 cell lines. Human dural fibroblasts stimulated with IL27 cytokine (100 ng/ml, 1 hour) were used as a positive control (**K**) Proliferation of KOPTK1 cells in the presence or absence of CXCL10 (100 ng/ml). Data are shown as mean ± SD for 1 of 3 independent experiments performed in triplicate. Migration of (**L**) T-ALL cell lines and (**M**) primary cells through HUVEC upon CXCR3 knockout ± CXCL10 (100 ng/µl, 6h). (**N** and **O**). T-ALL cell lines were treated with CXCL10 (100 ng/ml), followed by Western blot analysis of the indicated proteins. Representative blot from 3 independent experiments. (**P**-**T**) DND41 cells were transduced with plasmids expressing transcript variants of CXCR3 (CXCR3 TV1, CXCR3 TV2, CXCR3 TV1 + TV2) or a negative control plasmid (CON). (**P**) *CXCR3* mRNA, (**Q**) CXCR3 protein, and cell surface levels of (**R**) CXCR3, (**S**) CCR7, and (**T**) CXCR4 following *CXCR3* overexpression in DND41cells. Data represents the mean ± SD of 3 independent experiments. Representative blots from at least 3 separate

experiments. Representative histograms (left); MFI ± SD, 3 independent experiments (right). (**U**) Cell growth (**V**) cell cycle and (**W**) apoptosis in of DND41 cells transduced with plasmids expressing *CXCR3* (CXCR3 TV1, CXCR3 TV2, CXCR3 TV1 + TV2) and a negative control (CON). (**U**) Data are shown as mean ± SD for 1 of 3 independent experiments performed in triplicate; repeated measure ANOVA with Tukey's multiple comparisons test. (**R**-**T**). Representative histograms from 1 of 3 independent experiments. Migration of DND41 cells with forced expression of CXCR3 through a 3 μm porous membrane towards (**X**) CXCL9 (100 ng/ml) and (**Y**) CXCL11 (100 ng/ml). Data are shown as mean ± SD, 3 separate experiments performed in duplicate. (**Z**) Total number of T-ALL cells (human $CD45^+$) isolated from organs of NSG mice (n = 6/group) engrafted with transduced DND41 cells ($10^6$ cells/mouse). Mice were euthanized when moribund. (**H, K** and **U**) Repeated measure ANOVA with Tukey's multiple comparisons test. (**A-C, L, M, X** and **Y**) Two-way ANOVA with Tukey's multiple comparison correction, (**G**, **I**, **P**, **R**-**T** and, **Z**) One way ANOVA with Tukey's multiple comparison test ****$P$ < 0.0001.

**Supplemental Figure 4. USP7 regulates CXCR3 expression and protein interactions.** (**A**) qRT-PCR of *CXCR3* expression in KOPTK1 and PER117 cells transduced with shRNAs targeting USP7 (shUSP7 1 and shUSP7 2) and scrambled control (shNC). Data means ± SD for three independent experiments. One-way ANOVA with Tukey's multiple comparisons test; ****$P$ < 0.0001. (**B**) Immunoprecipitation analyses performed on HEK293 cells expressing Flag-wild-type (USP7 WT) and Flag-catalytically inactive $USP7^{C233S}$ mutant (USP7 CS). Representative blots from 3 independent experiments.

**Supplemental Figure 5. T-ALL induces CXCL10 expression in the meningeal microenvironment.** (**A**) CXCL9 levels were determined by ELISA in the blood serum, BM serum and CSF of T-ALL (ΔE-NOTCH1) and control (CON) mice (n = 7/group) (**B**) Confocal imaging of whole mount meninges from T-ALL (ΔE-NOTCH1) and control (CON) mice. Meningeal tissue was fixed and immunolabeled with an anti-CXCL9 (red) antibody. Leukemic cells expressed GFP

marker (green) (n = 3/group). ELISA for (**C**) CXCL10 and (**D**) CXCL9 in the blood serum, BM serum and CSF of NSG mice xenografted with KOPTK1 and PER117 cells ($10^6$ cells/mouse). Samples were collected at Day 1 and from moribund mice, respectively. (**E**) ELISA analysis of CXCL10 and (**F**) CXCL9 levels in the CSF of terminally ill NSG mice inoculated with DND41 cells ($10^6$ cells/mouse) transduced to express CXCR3 (CXCR3 TV1, CXCR3 TV2, CXCR3 TV1 + TV2) or a negative control (CON) (n = 8/group). (**G**) Flow cytometric quantification of T-ALL cells (GFP+/CD45+) in different organs of leukemic CXCL10 WT and CXCL10 KO mice (n = 10/group). The mice were euthanized when moribund. (**H**) Homing of T-ALL cells in the BM and meninges at 24 h. *Cxcl10* knockout mice (CXCL10 KO) and control B6 mice (CXCL10 WT) (n = 5/group) received intravenously $10^7$ $GFP^+$ Δ*E-NOTCH1*-transformed cells. (**I**) Apoptosis (Annexin $V^+$) in T-ALL ($GFP^+$) cells isolated from the meninges and BM of leukemia bearing CXCL10 KO and CXCL10 WT mice. (**J**) Total number of T-ALL cells ($GFP^+$/$CD45^+$) in the blood, liver, spleen, thymus, lung and testis in leukemic CXCL10 KO and CXCL10 WT mice, and non-leukemic control animals (CON) (n = 6/group). Samples were collected at three time points (Day 12, 20 and 35). (**K**) CXCL10 in the blood serum of leukemia-bearing CXCL10 KO and CXCL10 WT mice, and non-leukemic controls (CON) at three time points (n = 6/group). (**L**) *Cxcl9* by qRT-PCR in stromal ($CD45^-$) and hematopoietic ($CD45^+$) cells isolated from the bone marrow (BM), meninges (Mn) and thymus (Thy) of *ΔE-NOTCH1* mice (n = 4/group). (**M**) Gating strategy for identification of dural meningeal stromal and hematopoietic cell subsets. Hematopoietic cells were separated from stromal cells using $CD45^+$ magnetic beads, followed by downstream flow cytometry analyses. (**N** and **O**) CXCL10 expression in endothelial cells and vSMCs from various organs of ΔE-NOTCH1 T-ALL mice (MFI ± SD; n = 4/group). CXCL10 levels by ELISA in cell culture medium upon co-culture of (**P**-**U**) T-ALL cell lines (KOPTK1 and PER117) or primary T-ALL cells (Pt #2, Pt #4) with leptomeningeal cells (LeC), HUVEC and human primary dural microvascular endothelial cells (DuEC). Means ± SD for one of three independent experiments performed in triplicate. *CXCL10 mRNA* expression upon co-culture of (**V**-**X**) T-ALL cell lines and (**Y**-**AA**) primary T-ALL cells with

human primary pericytes (Per), dural fibroblasts (DuF) and leptomeningeal cells (LeC). After co-culturing, the cells were separated by FACS prior to RNA extraction and qRT-PCR. Data show means ± SD for one of three independent experiments performed in triplicate. (**A, J** and **K**) Two-way ANOVA with Tukey's multiple comparison correction. (**G, H** and **I**) unpaired t test with Holm-Sidak correction for multiple testing. (**C-F, L, N-AA**) One-way ANOVA with Tukey's correction for multiple comparison ***$P < 0.0005$; ****$P < 0.0001$.

**Supplemental Figure 6. CXCL10–CXCR3 signaling promotes T-ALL cell migration to meningeal stromal cells.** (**A**) T-ALL cell lines (KOPTK1, PER117) and (**B**) primary T-ALL cells (Pt #2, Pt #4) were pretreated with a CXCR3 antagonist (AMG-487; 1.5 µg, 30 minutes), followed by cell migration through the 3 µm porous membrane towards human primary meningeal stromal cells: Per, pericytes; DuF, dural fibroblasts; LeC, leptomeningeal cells, DuEC, dural endothelial cells (6 h). Data are shown as mean ± SD, 3 separate experiments performed in duplicate. Migration of (**C**) T-ALL cell lines and (**D**) primary T-ALL cells (6 h, 3µm) to meningeal stromal cells. Stromal cells were pretreated with/without CXCL10 blocking antibody (1.5 µg, 30 min). Data are shown as mean ± SD, 3 separate experiments performed in duplicate. (**E**) Migration of T-ALL cell lines, KOPTK1 and PER117, (6 h) towards conditioned medium (CM) from meningeal stromal cells in the presence or absence of CXCL10 blocking antibody (1.5 µg). Data are shown as mean ± SD, 3 separate experiments performed in duplicate. (**F**) *CXCL10* mRNA (qRT-PCR) and (**G**) protein levels (Western blot) in human primary meningeal stromal cells transduced with sgRNAs targeting CXCL10 (CXCL10 KO1 or CXCL10 KO2) compared to nonspecific control sgRNA (sgCtrl). Data are means ± SD for three independent experiments. Representative blots from at least 3 separate experiments. (**H**) Migration of T-ALL cell lines towards CM from meningeal stromal cells carrying CRISPR/Cas9-mediated knockout of *CXCL10* (CXCL10 KO1 or CXCL10 KO2) or control cells (sgCtrl) (6 h, 3 µm). Data are shown as mean ± SD, 3 separate experiments performed in duplicate. (**I**) Schematic of the tertiary co-culture system used to assess T-ALL cell

migration to the meningeal stroma through an endothelial monolayer grown on 5 μm porous membrane inserts. Migration of T-ALL cells (KOPTK1) toward human primary (**J**) dural fibroblasts (DuF) and (**K**) leptomeningeal cells (LeC) through a monolayer of dural endothelial cells (DuEC) (6 h). (**L**) Schematic of the quaternary co-culture system used to assess T-ALL cell migration to the meningeal stroma through a bilayer of pericytes and endothelial cells. (**M** and **N**) Migration of KOPTK1 cells to (**M**) dural fibroblasts (DuF) and (**N**) leptomeningeal cells (LeC) through a bilayer of pericytes (Per) and dural endothelial cells (DuEC) (6 h). (**A**-**E** and **H**) unpaired t test with Holm-Sidak correction for multiple testing. (**F**, **J**, **K**, **M** and **N**) One-way ANOVA with Tukey's correction for multiple comparison. ****$P$ < 0 .0001.

**Supplemental Figure 7. CXCR3–CXCL10 signaling enhances T-ALL cell adhesion to the meningeal stroma.** (**A**) Adhesion of T-ALL cell lines (KOPTK1, PER117) to human primary meningeal stromal cells (Per, pericytes; DuF, dural fibroblasts; LeC, leptomeningeal cells, DuEC, dural endothelial cells) and poly-L-lysine negative control after 6 h incubation. Data are shown as mean ± SD, 3 independent experiments. (**B**) Schematic of the quaternary co-culture system used to assess T-ALL cell adhesion to the meningeal stroma following migration through a bilayer of pericytes and endothelial cells. Adhesion of KOPTK1 cells to human primary (**C**) dural fibroblasts (DuF) and (**D**) leptomeningeal cells (LeC) after migration through a pericyte (Per)/dural endothelial cell (DuEC) bilayer (6 h). (**E**) Leukemic cells were pretreated with CXCR3 antagonist (AMG-487; 1.5 μg, 30 min), followed by co-incubation with meningeal stromal cells for 6 h. Non-attached cells were washed out and adherent T-ALL cells were sorted and enumerated. Data are shown as mean ± SD, 3 separate experiments. (**F**) Meningeal stromal cells were pretreated with CXCL10 blocking antibody (1.5 μg, 30 min) and co-incubated with T-ALL KOPTK1 and PER117 cells. Adherent T-ALL cells were enumerated after 6 h co-incubation. Data are shown as mean ± SD, 3 separate experiments. (**G**) VLA-4 in PER117 cells with/without *CXCR3* knockout (CXCR3 KO1, CXCR3 KO2, sgRNAs targeting CXCR3; SgCtrl, negative control) co-cultured in the presence or

absence of meningeal stromal cells. Representative histograms (top); MFI ± SD, 3 separate experiments (bottom). (**H**) VLA-4 levels in T-ALL cell lines co-cultured with meningeal stromal cells ± CXCR3 antagonist (AMG AMG-487; 1.5 µg, 30 min pretreatment of T-ALL cells). Representative histograms (left); MFI ± SD, 3 separate experiments (right). (**I**-**K**) *VCAM1* mRNA levels in meningeal stromal cells co-cultured with PER117 cells carrying CXCR3 knockout (CXCR3 KO1, CXCR3 KO2, sgRNAs targeting CXCR3; SgCtrl, negative control) (6 h). After incubation the cells were sorted by FASC followed by qRT-PCR for the specified cells (red font). Data are shown as mean ± SD, 3 separate experiments. (**L** and **M**) T-ALL cells were pretreated with AMG AMG-487 (1.5 µg, 30 min) followed by co-culture with meningeal stromal cells (6 h). *VCAM1* mRNA was measured by qRT-PCR in the indicated cells (red font). Data are shown as mean ± SD, 3 separate experiments. (**N**) Expression of VLA-4 in PER117 cells co-cultured with meningeal stromal cells (6h) with/without CXCL10 knockout (CXCL10 KO1, CXCL10 KO2, sgRNAs targeting CXCL10*;* SgCtrl, negative control). Representative histograms (top) and MFI ± SD, 3 separate experiments (bottom). (**O**) VLA-4 expression in T-ALL cell lines co-cultured with meningeal stromal cells pretreated with CXCL10 blocking antibody (1.5 µg, 30 min). Representative histograms (left); MFI ± SD, 3 independent experiments (right). (**P**-**R**) *VCAM1* mRNA in meningeal stromal cells upon CXCL10 knockout (CXCL10 KO1, CXCL10 KO2, sgRNAs targeting CXCL10*;* SgCtrl, negative control), and cultured alone or co-cultured (red) with T-ALL cell lines. Data are shown as mean ± SD, 3 separate experiments. (**S** and **T**) *VCAM1* mRNA in co-cultured T-ALL cells with meningeal stromal cells, which were pretreated with CXCL10 blocking antibody (1.5 µg, 30 min). Gene expression was evaluated for the cells indicated in red font. Data are shown as mean ± SD, 3 separate experiments. (**A**, **C, D, H**-**N**, **P**-**R**) One-way ANOVA with Tukey's multiple comparison test. (**E**-**G**) Two-way ANOVA with Tukey's multiple comparison test. (**H** and **O**) unpaired t test with Holm-Sidak correction for multiple testing; $****P < 0.0001$.

**Supplemental Figure 8. Cytokine-mediated induction of CXCL10 in meningeal stromal cells in T-ALL**: (**A**) CXCL10 in culture medium by ELISA after culturing human primary meningeal cells for 6 h (Per, pericytes; DuF, dural fibroblasts; LeC, leptomeningeal cells, DuEC, dural endothelial cells) in conditioned medium (CM, 48 h) from T-ALL cell lines (KOPTK1, PER117). CM and stomal cell culture medium were used as controls. Mean ±SD, 3 separate experiments performed in duplicate. (**B**) human cytokine array (Eve Technologies) using culture medium from T-ALL cells (KOPTK1) co-cultured with meningeal stromal cells for 6 h. Each sample was tested in duplicate. Shown top 5 significant cytokines. (**C**) *CXCL10* mRNA levels in meningeal stromal cells stimulated with PDGFA (10 ng/ml) and IL7 (10 ng/ml) for 1 h. Data are shown as mean ± SD, 3 separate experiments. (**D**) ELISA measurements of TNFα, IL27 and IFNγ, in the blood serum of T-ALL mice (ΔE-NOTCH1), ΔE-NOTCH1 T-ALL mice with CXCL10 KO (CXCL10 KO), and non-leukemic control mice (CON) at three time points (Day 12. 20 and 35) (n = 6/group). (**E**) Cell surface expression of TNFR1, IL27Rα, IFNGR1 on primary human meningeal stromal cells (Per, pericytes; DuF, dural fibroblasts; LeC, leptomeningeal cells) co-cultured with PER117 cells. MFI ± SD, 3 separate experiments. (**F**) A schematic illustration of experiments involving stromal cells incubated with T-ALL CM (48h) ± blocking antibody. (**G**) *CXCL10* mRNA in human meningeal stromal cells incubated with T-ALL CM for 6h in the presence or absence of blocking antibody (TNFα, 0.5 µg/ml or IL27, 0.5 µg/ml; 1h). (**H**) A scheme: T-ALL transwell migration to stroma ± blocking antibody. (**I**) Migration of T-ALL cells pretreated with blocking antibody (TNFα, 0.5 µg or IL27, 0.5 µg; 1h) towards meningeal stromal cells (6h, 3 µm). (**J**) CXCL10 levels by ELISA in culture medium collected after 6 h of co-culture of meningeal stromal cells with T-ALL cells pretreated with TNFα or IL27 blocking antibody for 1 h. Mean ± SD, 3 separate experiments. (**K**) ELISA for CXCL10 in culture medium collected after co-culturing (6 h) meningeal stromal cells with T-ALL CM (48 h) in the presence or absence of TNFα or IL27 blocking antibody for 1 h. Mean ± SD, (n = 3). qRT-PCR and immunoblotting upon CRISPR/Cas9-mediated knockout of (**L**) *TNF* and (**M**) *IL27* in T-ALL cell lines (sgRNAs targeting *TNF*, TNFα KO1 and TNFα KO2; sgRNAs

targeting *IL27*, IL27 KO1 and IL27 KO2; control sgRNA, sgCtrl). Mean ± SD for three independent experiments. Representative blots from at least 3 independent experiments. (**N** and **O**) *CXCL10* mRNA in meningeal stromal cells co-cultured with T-ALL cells with/without *TNF* or *IL27* knockout. Migration of T-ALL cell lines harboring deletion of (**P**) *TNF* or (**Q**) *IL27* and control cells towards meningeal stromal cells (6 h, 3 µm). (**A**, **L** and **M**) One-way ANOVA with Tukey's multiple comparison correction. (**C**, **E**, **G**, **I**, **J** and **K**) unpaired t test with Holm-Sidak correction for multiple testing. (**D**, **N**-**Q**) Two-way ANOVA with Tukey's multiple comparison test $^*P < 0.05$; $^{**}P < 0.005$; $^{***}P < 0.0005$; $^{****}P < 0.0001$.

**Supplementary Figure 9. Testing stromal and hematopoietic cell populations in murine dural meninges by flow cytometry.** Schematic representation of the gating strategy for identification of (**A**) dural meningeal stromal cells and (**B**) dural T-ALL and normal immune cell subsets. Hematopoietic cells were separated from stromal cells using $CD45^+$ magnetic beads, followed by downstream flow cytometry analyses.
