# Supplementary material for: T cell acute lymphoblastic leukemia exploits a neural proinflammatory pathway to colonize the meninges
Source: J Clin Invest. 2025 Oct 23;136(2):e188888. doi: 10.1172/JCI188888 (PMC12807478; doi:10.1172/JCI188888)

Figure 1K

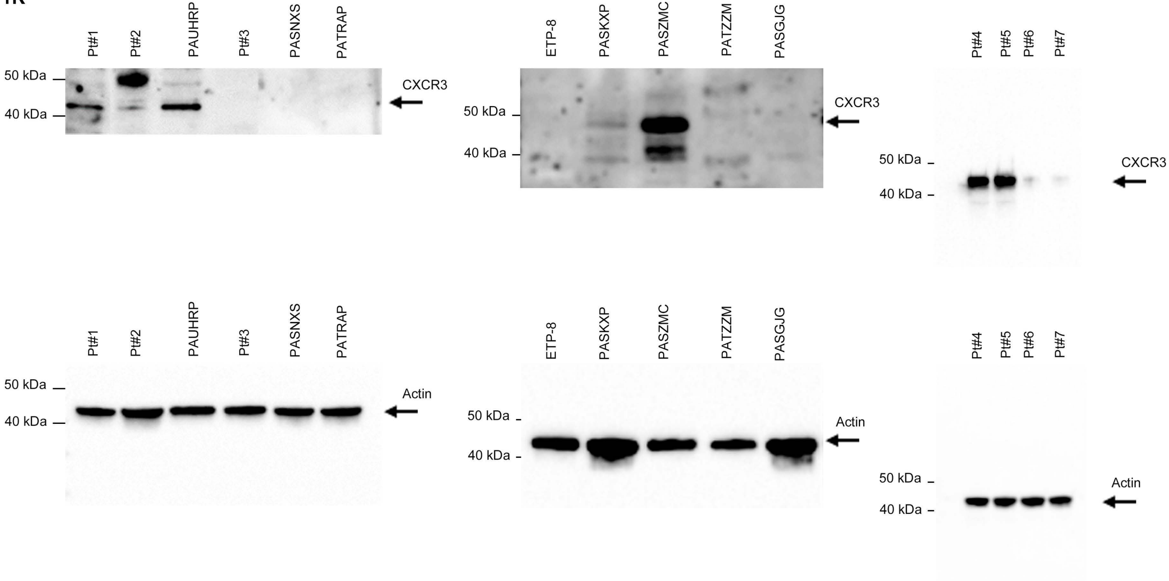

Pt#1
Pt#2
PAUHRP
Pt#3
PASNXS
PATRAP
50 kDa
40 kDa
CXCR3
ETP-8
PASKXP
PASZMC
PATZZM
PASGJG
50 kDa
40 kDa
CXCR3
Pt#4
Pt#5
Pt#6
Pt#7
50 kDa
40 kDa
CXCR3
Pt#1
Pt#2
PAUHRP
Pt#3
PASNXS
PATRAP
50 kDa
40 kDa
Actin
ETP-8
PASKXP
PASZMC
PATZZM
PASGJG
50 kDa
40 kDa
Actin
Pt#4
Pt#5
Pt#6
Pt#7
50 kDa
40 kDa
Actin

Figure 2G

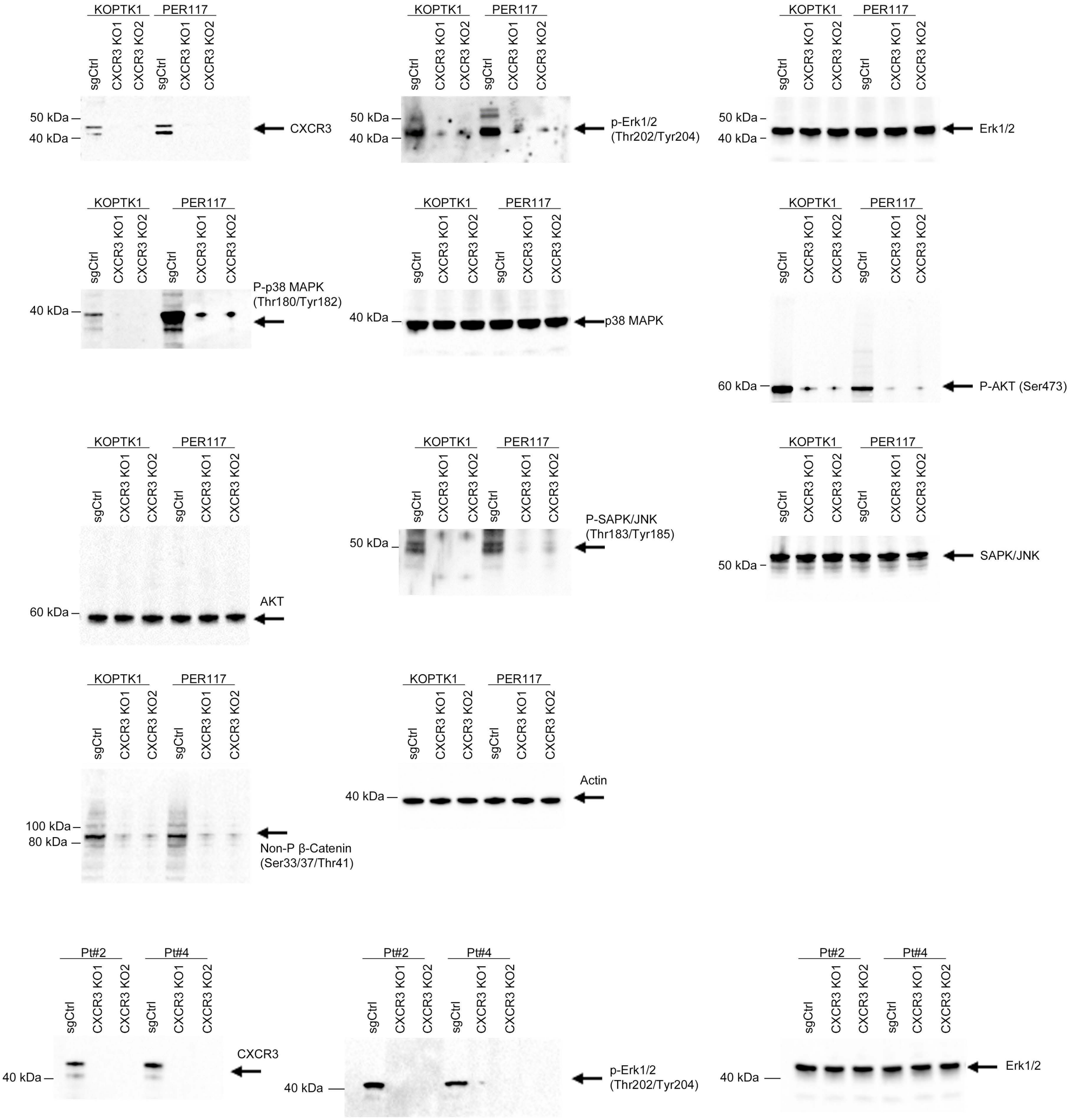

KOPTK1
PER117
sgCtrl
CXCR3 KO1
CXCR3 KO2
50 kDa
40 kDa
CXCR3
p-Erk1/2
(Thr202/Tyr204)
Erk1/2
P-p38 MAPK
(Thr180/Tyr182)
p38 MAPK
60 kDa
P-AKT (Ser473)
AKT
P-SAPK/JNK
(Thr183/Tyr185)
SAPK/JNK
100 kDa
80 kDa
Non-P β-Catenin
(Ser33/37/Thr41)
Actin
Pt#2
Pt#4

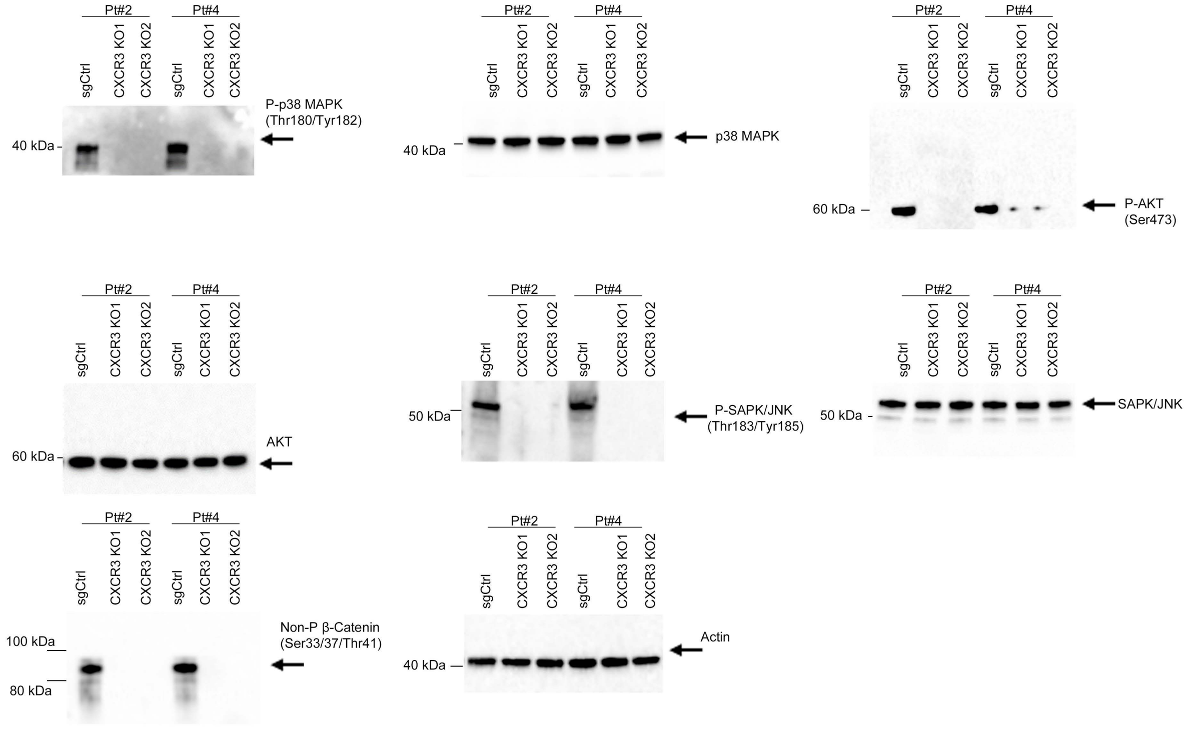
Pt#2
Pt#4
sgCtrl
CXCR3 KO1
CXCR3 KO2
P-p38 MAPK (Thr180/Tyr182)
40 kDa
p38 MAPK
60 kDa
P-AKT (Ser473)
AKT
50 kDa
P-SAPK/JNK (Thr183/Tyr185)
SAPK/JNK
100 kDa
80 kDa
Non-P β-Catenin (Ser33/37/Thr41)
Actin

Figure 2H

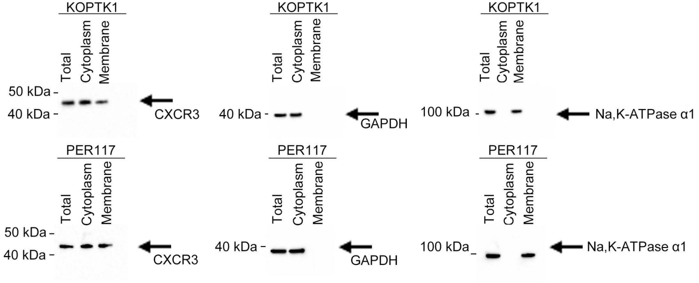
KOPTK1
PER117
Total
Cytoplasm
Membrane
50 kDa
40 kDa
100 kDa
CXCR3
GAPDH
Na,K-ATPase α1

Figure 2I

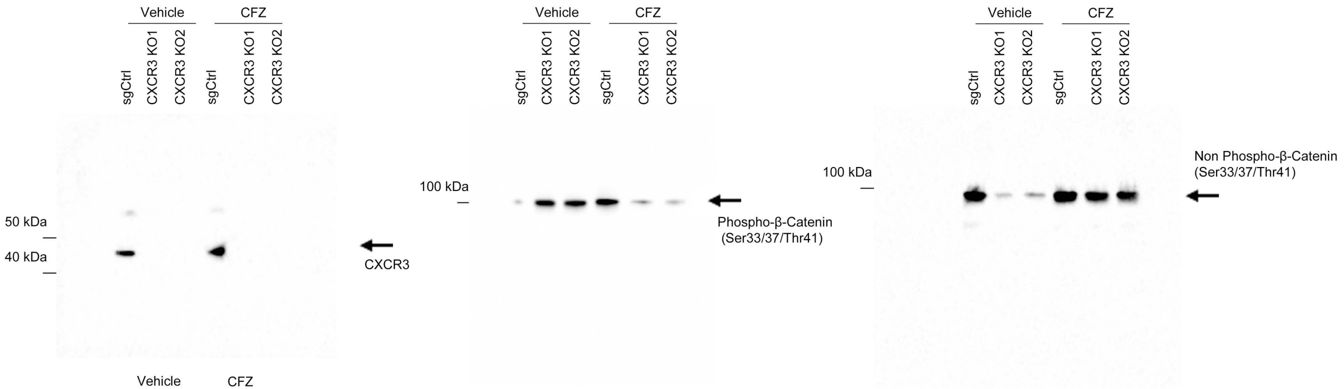

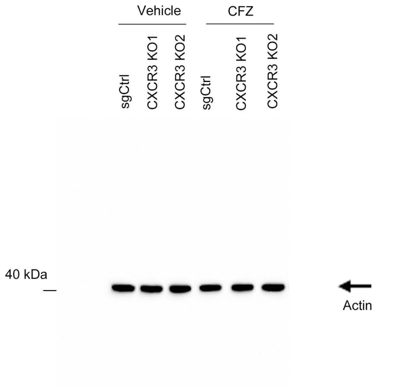

Supplemental Figure 2B

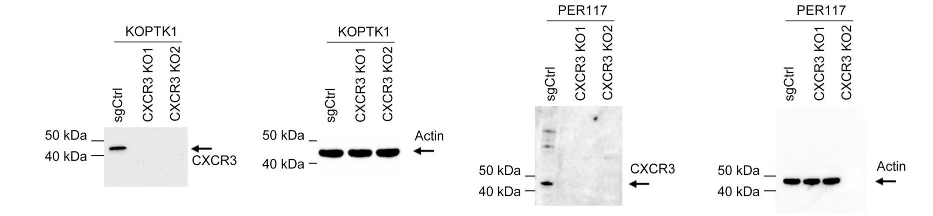

Supplemental Figure 2G

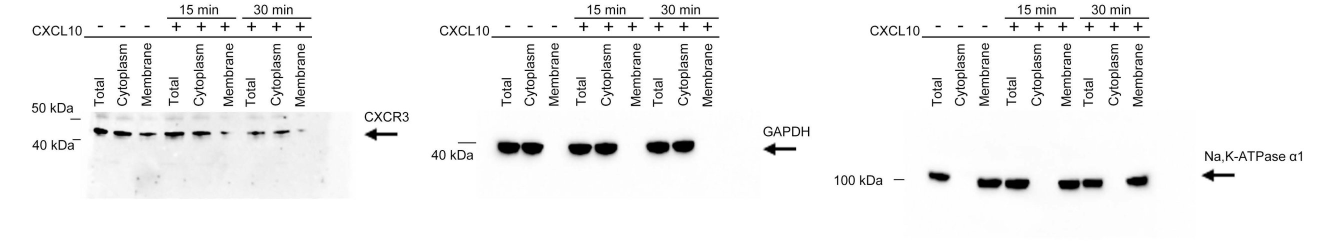

Supplemental Figure 2H

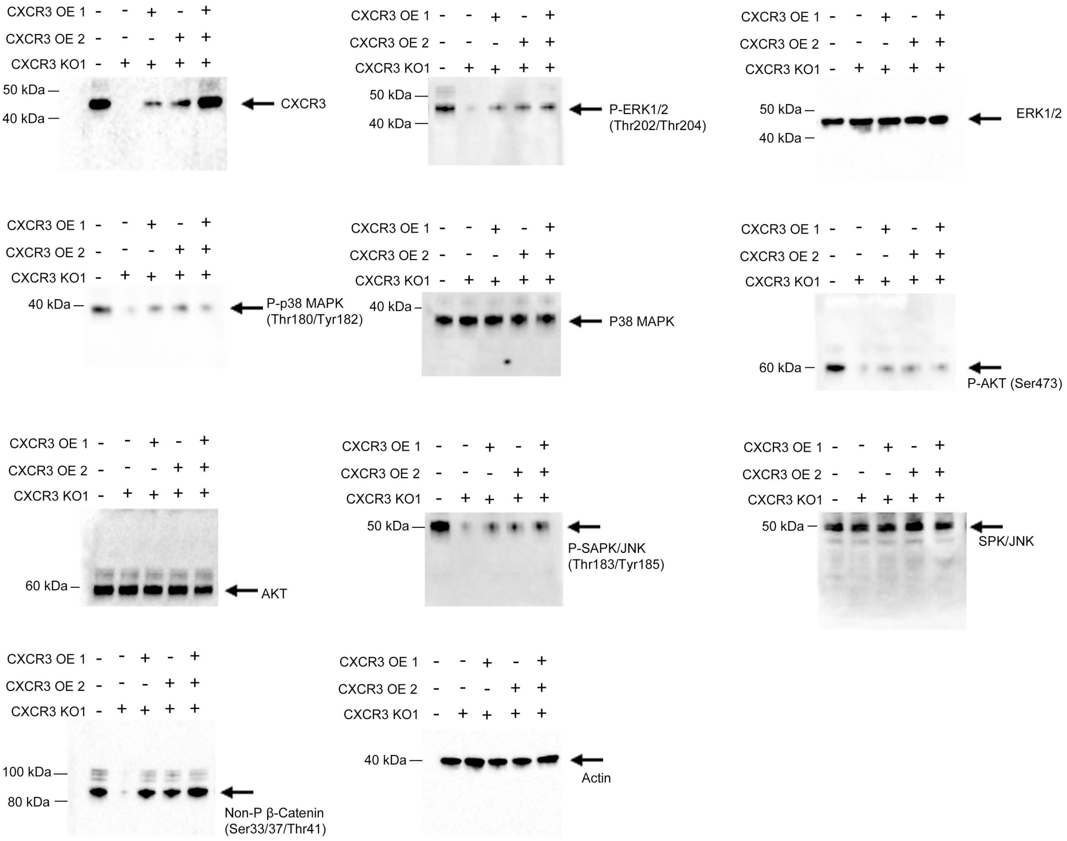

Supplemental Figure 2J

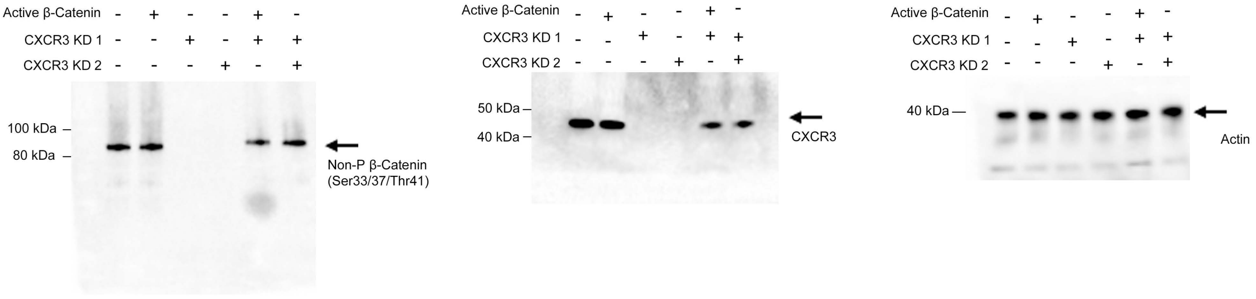

Supplemental Figure 2 L

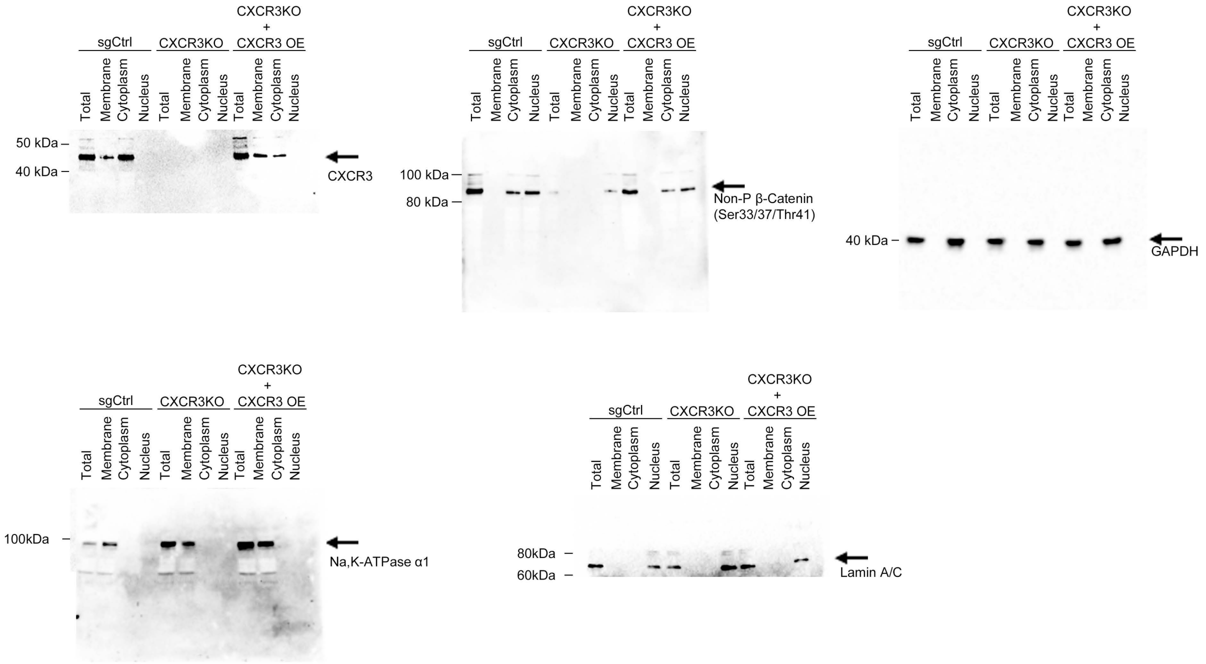

Figure 3F

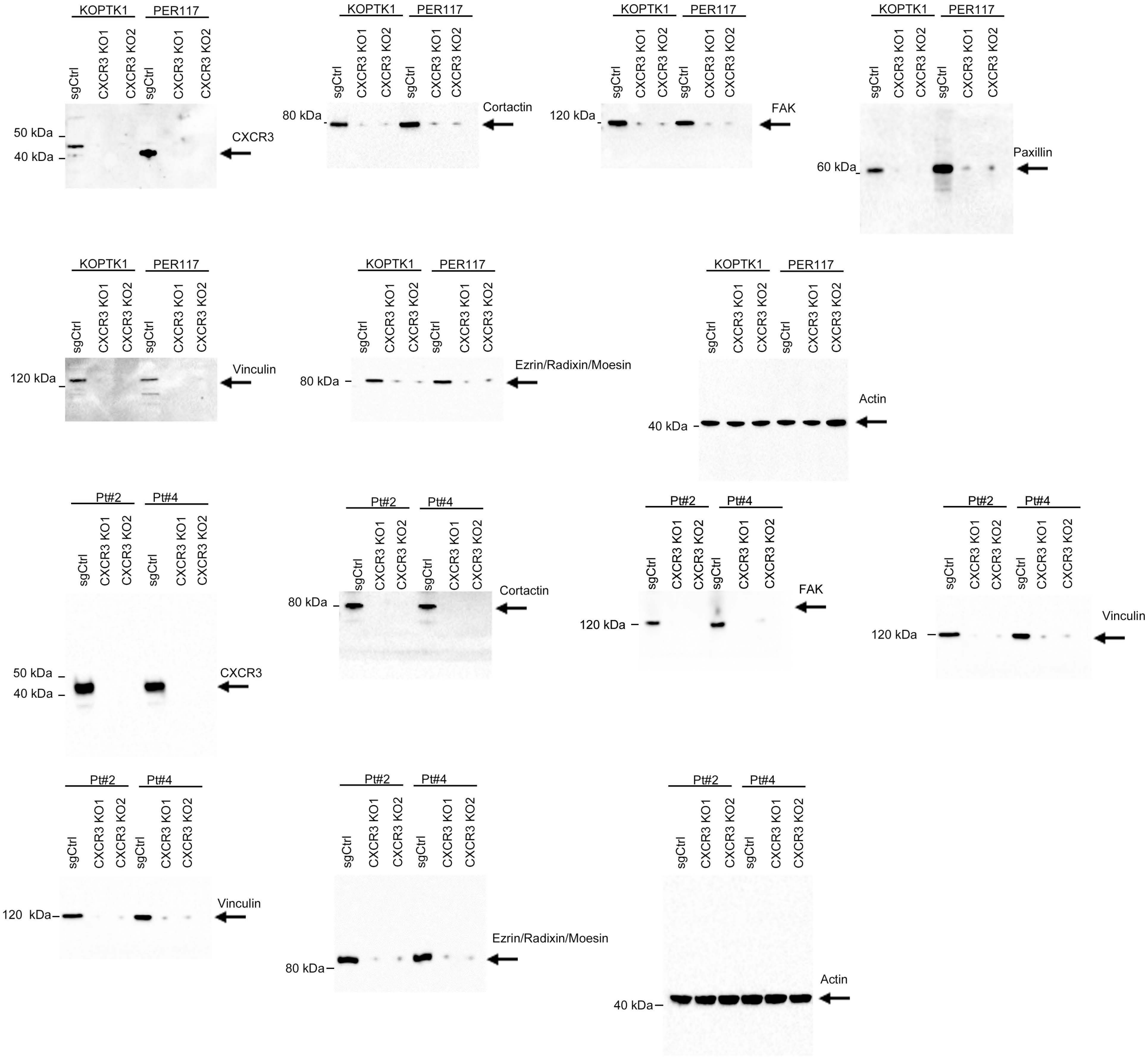
KOPTK1
PER117
sgCtrl
CXCR3 KO1
CXCR3 KO2
50 kDa
40 kDa
CXCR3
80 kDa
Cortactin
120 kDa
FAK
60 kDa
Paxillin
Vinculin
Ezrin/Radixin/Moesin
Actin
Pt#2
Pt#4

Figure 3G

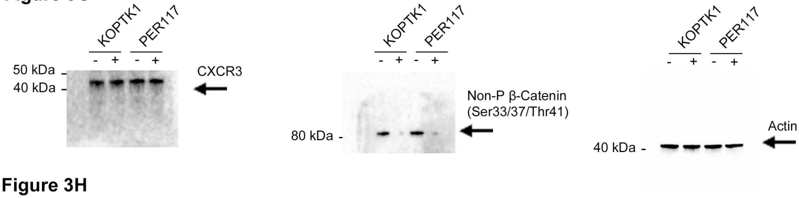

Figure 3H

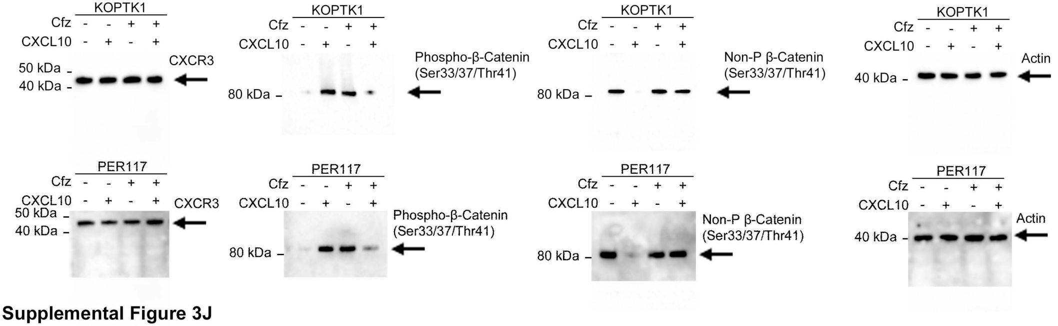

Supplemental Figure 3J

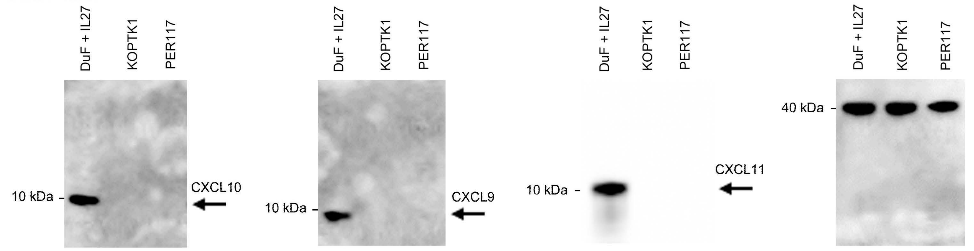

Supplemental Figure 3N

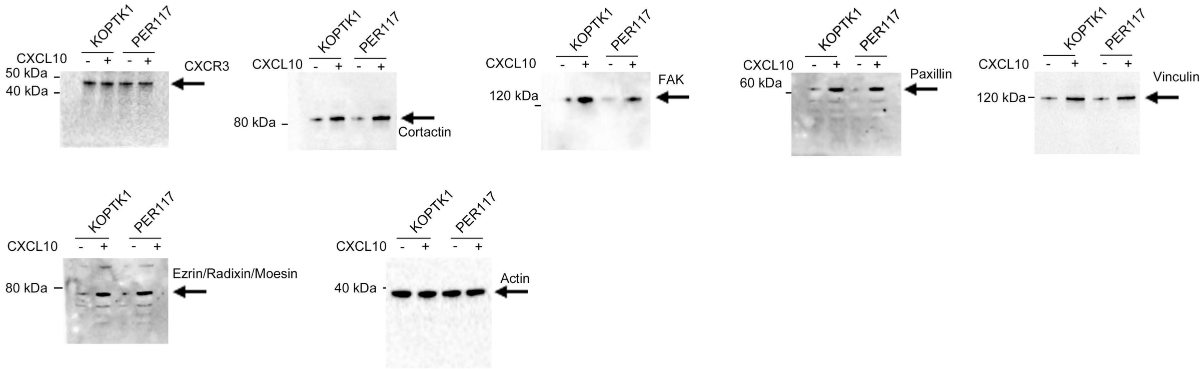

Supplemental Figure 3O

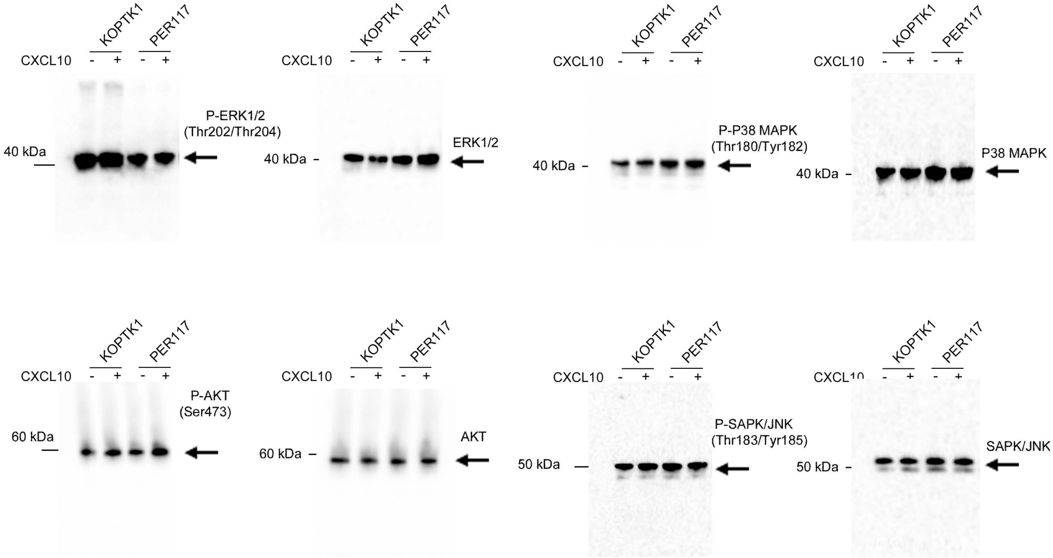

Figure 4B

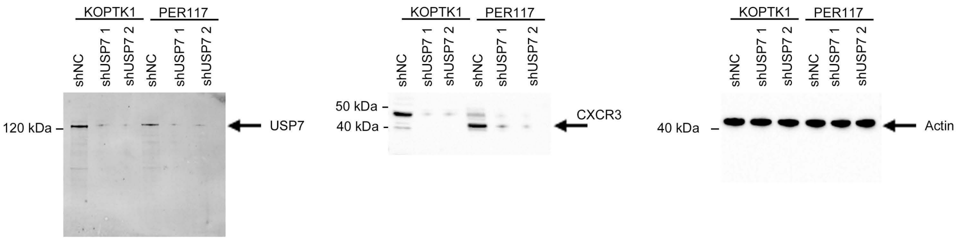

Figure 4D

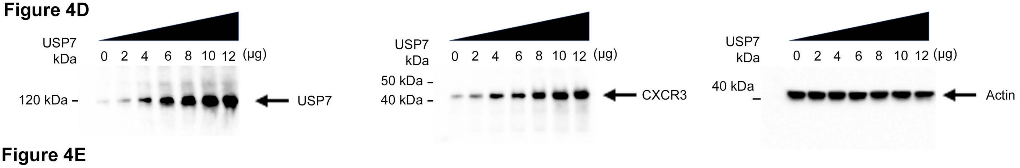

Figure 4E

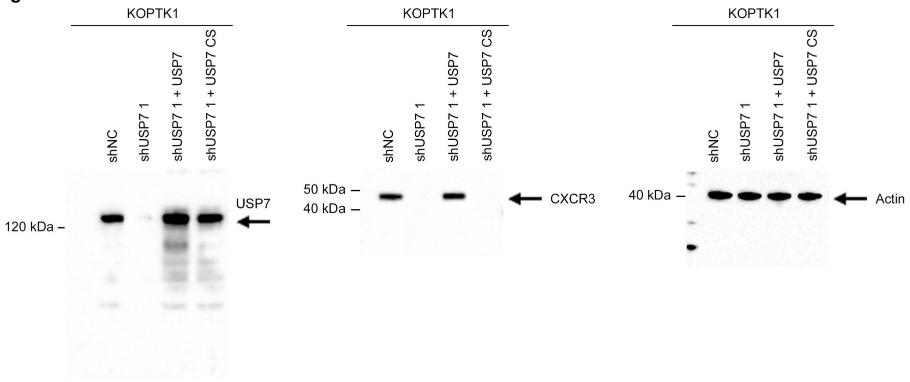

Figure 4F

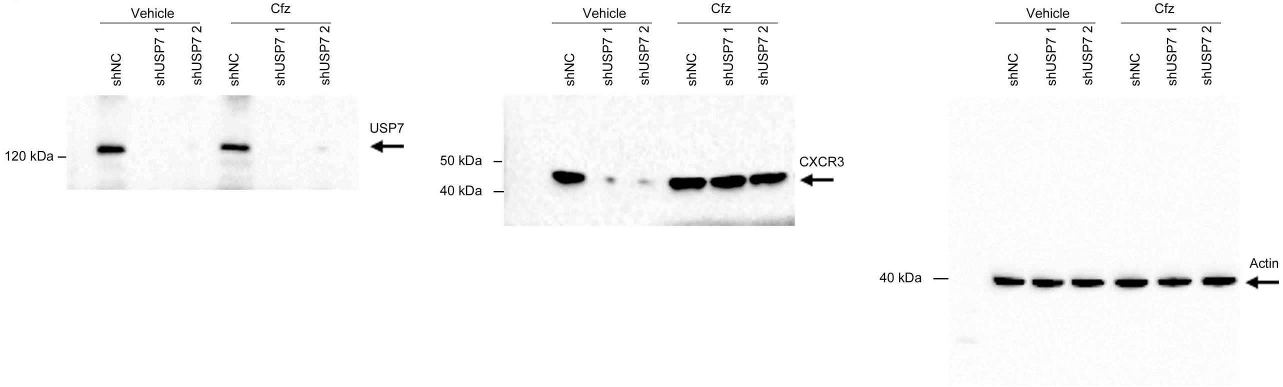

Figure 4G

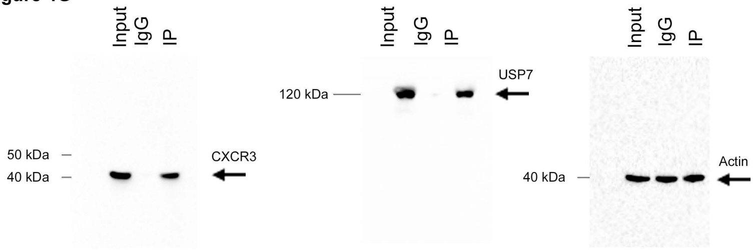

Figure 4H

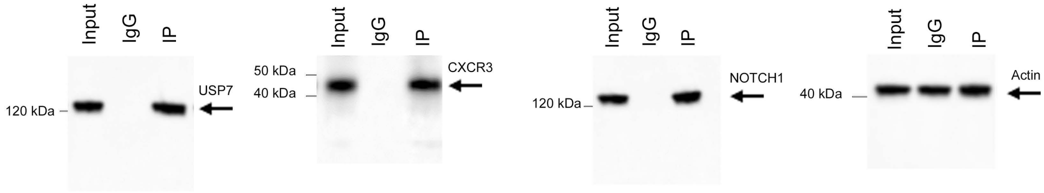

Figure 4I

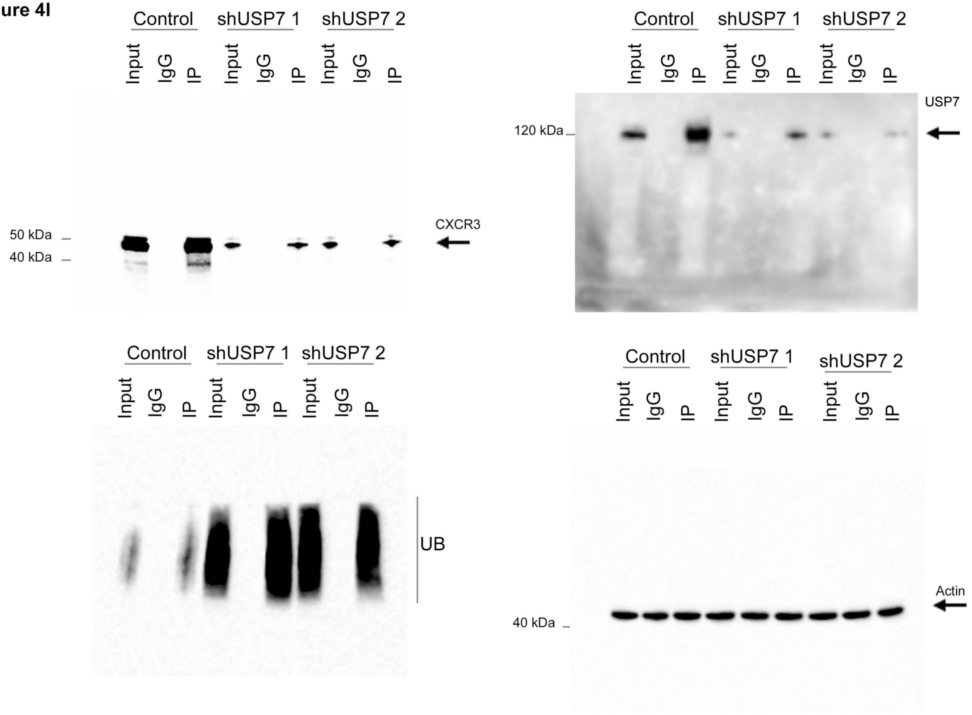

Figure 4J

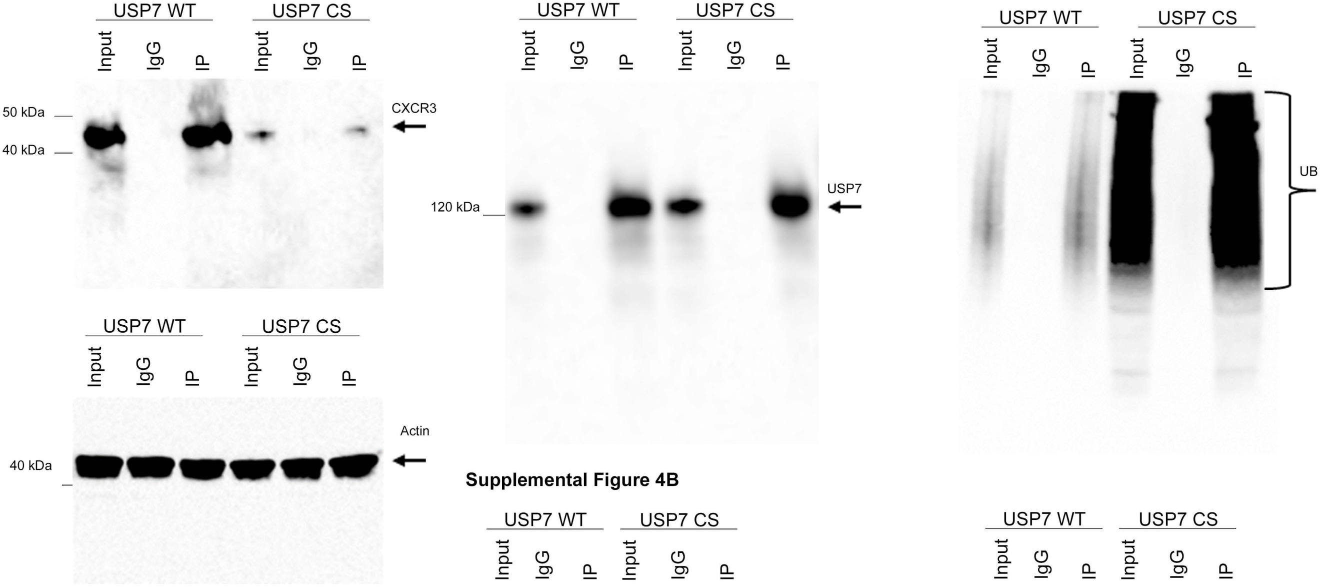

Supplemental Figure 4B

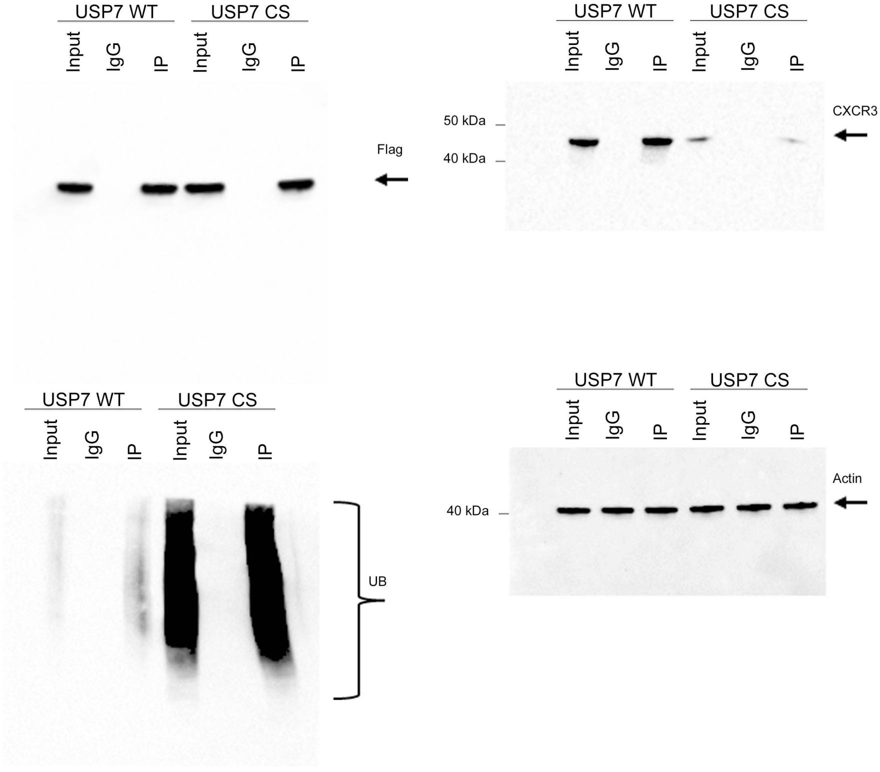

Figure 4K

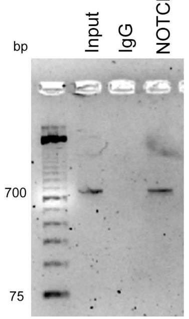

Supplemental Figure 6G

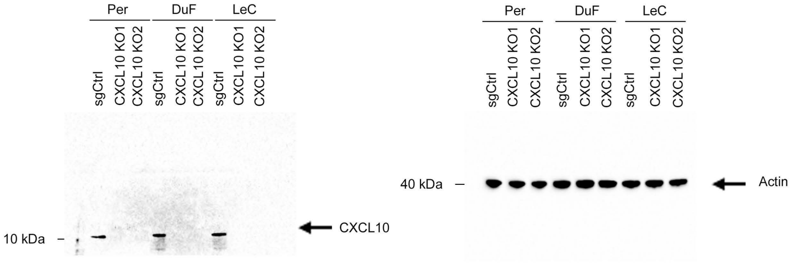

Supplemental Figure 8L

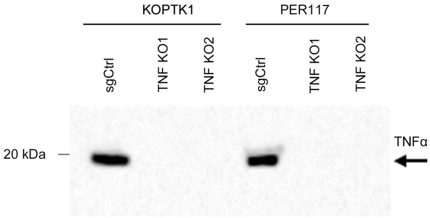

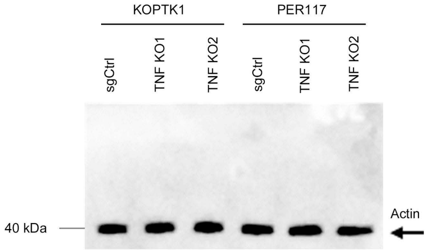

Supplemental Figure 8M

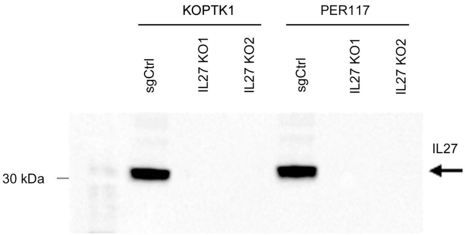

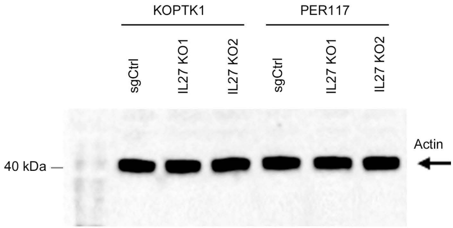

Supplement: Unedited blot and gel images [file jci-136-188888-s232.pdf]
